# A Bridge too Far? Comparison of Transition Metal Complexes of Dibenzyltetraazamacrocycles with and without Ethylene Cross-Bridges: X-ray Crystal Structures, Kinetic Stability, and Electronic Properties

**DOI:** 10.3390/molecules28020895

**Published:** 2023-01-16

**Authors:** Ashlie N. Walker, Megan A. Ayala, Somrita Mondal, Mackenzie C. Bergagnini, Phuong John D. Bui, Stephanie N. Chidester, Chad I. Doeden, Louise Esjornson, Brian R. Sweany, Leslie Garcia, Jeanette A. Krause, Allen G. Oliver, Timothy J. Prior, Timothy J. Hubin

**Affiliations:** 1 Department of Chemistry and Physics, Southwestern Oklahoma State University, Weatherford, OK 73096, USA; 2 Department of Chemistry, University of Cincinnati, Cincinnati, OH 45220, USA; 3 Department of Chemistry and Biochemistry, University of Notre Dame, Notre Dame, IN 46556, USA; 4 Department of Chemistry, School of Natural Sciences, University of Hull, Kingston Upon Hull HU6 7RX, UK

**Keywords:** tetraazamacrocycle, transition metal complex, cross-bridged tetraazamacrocycle, X-ray crystal structure, kinetic stability

## Abstract

Tetraazamacrocycles, cyclic molecules with four nitrogen atoms, have long been known to produce highly stable transition metal complexes. Cross-bridging such molecules with two-carbon chains has been shown to enhance the stability of these complexes even further. This provides enough stability to use the resulting compounds in applications as diverse and demanding as aqueous, green oxidation catalysis all the way to drug molecules injected into humans. Although the stability of these compounds is believed to result from the increased rigidity and topological complexity imparted by the cross-bridge, there is insufficient experimental data to exclude other causes. In this study, standard organic and inorganic synthetic methods were used to produce unbridged dibenzyl tetraazamacrocycle complexes of Co, Ni, Cu, and Zn that are analogues of known cross-bridged tetraazamacrocycles and their transition metal complexes to allow direct comparison of molecules that are identical except for the cross-bridge. The syntheses of the known tetraazamacrocycles and the new transition metal complexes were successful with high yields and purity. Initial chemical characterization of the complexes was conducted by UV-Visible spectroscopy, while cyclic voltammetry showed more marked differences in electronic properties from bridged versions. Direct comparison studies of the unbridged and bridged compounds’ kinetic stabilities, as demonstrated by decomposition using high acid concentration and elevated temperature, showed that the cyclen-based complex stability did not benefit from cross-bridging. This is likely due to poor complementarity with the Cu^2+^ ion while cyclam-based complexes benefited greatly. We conclude that ligand–metal complementarity must be maintained in order for the topological and rigidity constraints imparted by the cross-bridge to contribute significantly to complex robustness.

## 1. Introduction

**Figure 1:**
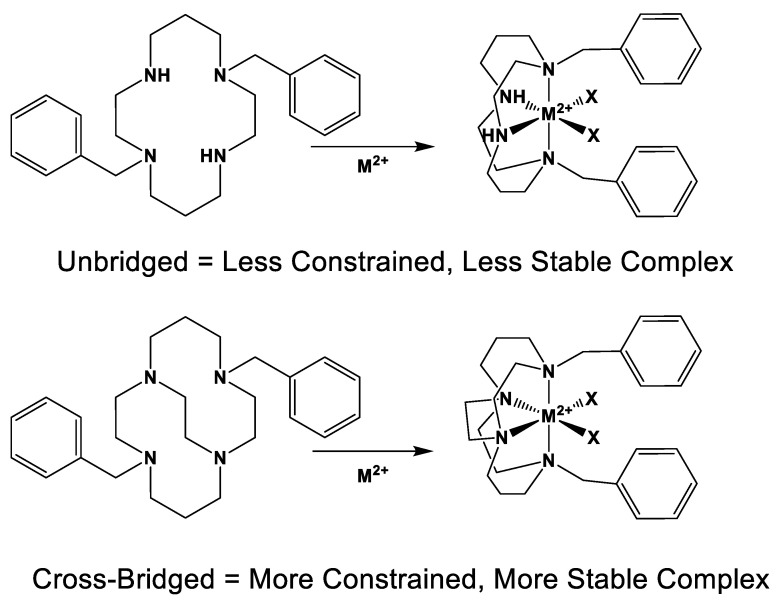
Unbridged vs. cross-bridged tetraazamacrocycles and complexes.

*Tetraazamacrocycles* are ubiquitous ligands for transition metal ions that contain four nitrogen atoms tied together in a ring by carbon chains. The stability of transition metal complexes can be characterized by their kinetic stability (how long it takes to decompose the complex under harsh conditions) and/or their thermodynamic stability (binding constants which can often be determined by potentiometric titrations for metal complexes). Unfortunately, potentiometric titrations for some of the most stable transition metal complexes, those with cross-bridged tetraazamacrocycles, are often not possible because the proton sponge nature [1] of these ligands means the last proton(s) are never removed from the ligand to allow metal ion binding in aqueous solutions. Instead, kinetic stabilities under harsh aqueous conditions of these cross-bridged tetraazamacrocycle transition metal complexes may be the main technique available to compare complex stabilization by new cross-bridged ligands [2]. Inorganic chemists have learned that the kinetic stability of metal complexes can be increased by many orders of magnitude by increasing the *topological complexity* (number of links between the nitrogen atoms) and rigidity of the ligand as long as ligand–metal complementarity (size, geometry, and electronics match between metal ion and ligand) is maintained [3]. In general, complex kinetic stability decreases in the following series: bridged azamacrocycle ligand > azamacrocyclic ligand > linear ligand with more than one nitrogen > single nitrogen ligand (see Figure 1) [3].

*Cross-bridged* tetraazamacrocycles that have an additional two-carbon bridge between non-adjacent nitrogen atoms of a tetraazamacrocycle, which are particularly rigid and lead to very kinetically stable metal complexes, have been extensively studied by Weisman [1,2,4,5,6,7,8,9,10,11,12], Springborg [13,14,15], Hubin [16,17,18,19,20,21,22,23], Archibald [24,25,26,27,28,29,30], Tripier [31,32,33,34,35,36], and others [37,38,39]. This stability conferred on these transition metal complexes shows great promise in such applications as homogeneous catalysis [40,41,42,43], inorganic drug candidates [20,24,26,27,44,45,46], and biomedical imaging agents [2,7,9,11,30] where complex stability is required for success. However, specific studies where “control” metal complexes, identical in all ways except lacking the ligand cross-bridge, have not been prepared and characterized with respect to complex kinetic stability or other properties.

Electronic properties (specifically of the metal d-electrons) of tetraazamacrocycle transition metal complexes are influenced by their geometric structure and the pattern of the nitrogen atom substituents [47,48]. If these properties are very similar between bridged and unbridged complexes of the same metal ion, that indicates that the bridge has little effect on the d-electron configuration. The d-electron configuration would be most closely associated with thermodynamic stability, which would therefore be assumed to be approximately the same for the bridge/unbridged pair. However, if the kinetic stability of the bridged complex is much greater than its unbridged analogue, then these results would be consistent with the hypothesis that the topological complexity and additional rigidity of the cross-bridge is responsible for the additional kinetic stability rather than thermodynamic stabilization.

**Figure 2:**
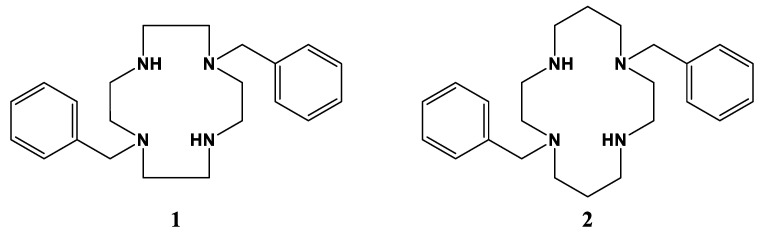
1,7-dibenzylcyclen (**1**) and 1,8-dibenzylcyclam (**2**).

We report here known dibenzyl cyclen (12-membered ring) and cyclam (14-membered ring) tetraazamacrocycle ligands 1,7-dibenzylcyclen (**1**) and 1,8-dibenzylcyclam (**2**) (see Figure 2) and their Co, Ni, Cu, and Zn transition metal complexes. The cyclam ligand **2** has been complexed to a number of metal ions previously [49,50,51,52,53,54,55], but the characterization of its complexes has been limited. The cyclen analogue **1** has a long history [56] but only a few published complexes, most of which are large second- and third-row transition metal ions [54,57,58,59,60]. Therefore, we aimed to synthesize and characterize the complexes of these two analogous tetraazamacrocycle ligands for comparison. In particular, we aimed to contrast the properties of these unbridged complexes with the known cross-bridged complexes.

## 2. Results and Discussion

### 2.1. Complex Synthesis

Both ligands are known in the literature, and our syntheses yielded pure compounds (as assessed by comparison of NMR spectra) in high yield (57% yield for three steps for **1**; 75% yield for three steps for **2**). Complexation occurred as expected in methanol for both ligands with all four divalent metal ions (Co, Ni, Cu, and Zn) from their acetate salts. Macrocyclic complexes with acetate counter anions are typically hygroscopic oils, so we did not try to isolate them. Instead, we performed an anion metathesis reaction with ammonium hexafluorophosphate to give the [M(ligand)(acetate)]PF_6_ complexes, which precipitate out of methanol and are non-hygroscopic powders. Formulas, yields, electrospray mass spec peaks, and elemental analysis data for all eight complexes are given in Table 1 and Table 2.

All the complexes were formed, as evidenced by the expected color changes and dissolution of the ligand and metal salt during the reactions. Additional evidence of complexation was shown by the multiple peaks in the electrospray mass spectrum for each complex containing both the metal and the ligand and sometimes other species as well (acetate, hexafluorophosphate, and water; see Table 1). Yields were typically 50–75%, which are acceptable. These yields were likely lowered for several of the complexes by considerable solubility in the methanol solution they were precipitated from. Indeed, [Ni(**2**)(OAc)]PF_6_ never did precipitate from methanol. Instead, it was obtained by removing the methanol and stirring the residue in water to produce the pale blue powder product.

**Figure 3:**
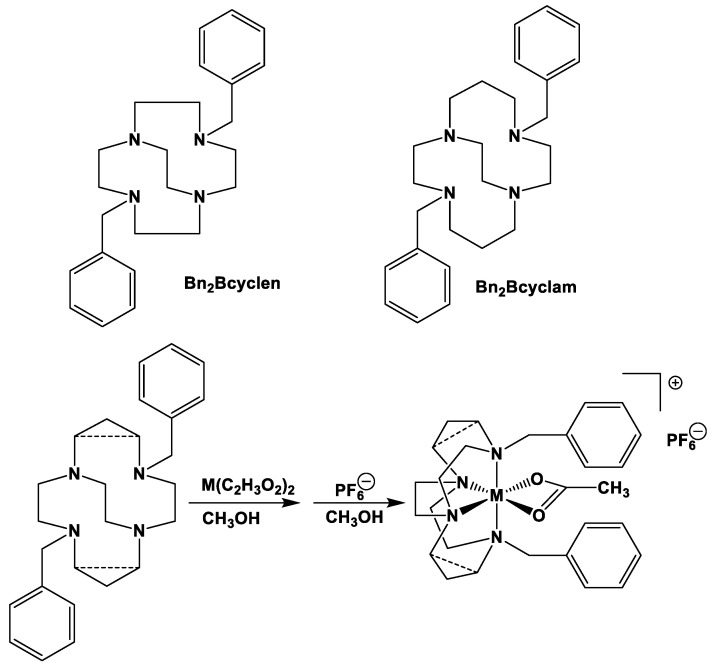
Cross-bridged ligands and complexes for comparison **[28]**.

Comparison to Cross-Bridged Complexes: The motivation of this work was to make complexes that differed from the known cross-bridged analogues by only the lack of the cross-bridge itself. Ligands **Bn_2_Bcyclen** and **Bn_2_Bcyclam** in Figure 3 have yielded Co, Ni, Cu, and Zn complexes in previous work in the Hubin labs [28]. First, the X-ray crystal structures of the unbridged ligand **1** and **2** complexes are presented and compared to the cross-bridged analogues. Next, UV-Vis, magnetic moment, and cyclic voltammetry experiments serve as points of comparison between the cross-bridged and unbridged complexes’ electronic properties, which probe their d-electron configurations. Differences in d-electron configurations should result in differences in thermodynamic stabilities between the bridged and unbridged analogues. Kinetic stability experiments are then presented that examine the effect of the topological and rigidity constraints associated with the cross-bridge.

### 2.2. X-ray Crystal Structures

The crystal structures of each metal complex are now described. There is further information and additional figures contained within the Supplementary Materials. In a small number of the structures, there is disorder. Where this is the case, the orientation and bond lengths described are for the major disorder component. One structure, [Cu(**2**)(OCHNMe_2_)_2_](PF_6_)_2_ · 2(OCHNMe_2_), has been previously published [54], but the complex was not completely characterized. It is included in the structural discussion for completeness, and its complete characterization (UV-Vis, cyclic voltammetry, and kinetic stability) appears for the first time here. [Ni(**1**)(OAc)]PF_6_ was reported in a similar acetone solvated crystal structure previously [54], again without complete characterization. The structure presented below for this complex is novel and free of solvate, so it has been included and used for other characterization studies.

#### 2.2.1. Complexes of **1**, Dibenzylcyclen

Three different nickel complexes with **1** were obtained, but the coordination about the Ni^2+^ ion in each of these was rather similar. The nickel was six coordinate and resided with distorted octahedral geometry and featured **1** forming a V-shape to coordinate in four positions on one side of the metal with two further cis coordination sites at the metal were occupied by other ligands.

**Figure 4:**
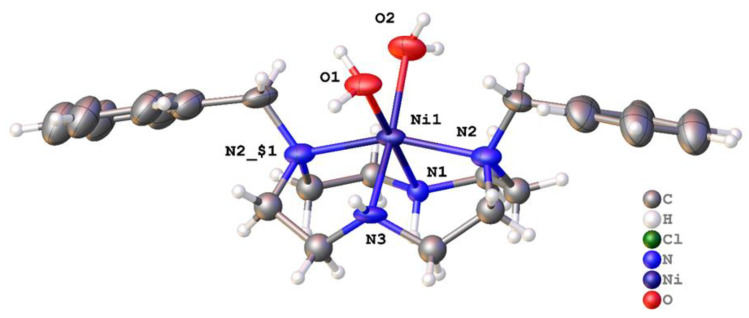
Molecular structure of [Ni(**1**)(OH_2_)_2_]Cl_2_. The major disorder component is shown with atoms drawn as 30% probability ellipsoids. For clarity, unbound chloride is not represented. Symmetry operation used for generating equivalent atoms: $1 = *y*, *x*, *z*.

The compound [Ni(**1**)(OH_2_)_2_]Cl_2_ (see experimental for why this complex is included) crystallized in the tetragonal space group *P*4_2_/*ncm* (origin choice 2) with the nickel ion sited on the mirror plane (Wyckoff position 8i). There was pronounced disorder in the orientation of the ligand that was related to the high symmetry. The distorted octahedral coordination geometry about the nickel center is shown in Figure 4. The four ligand atoms N1, N3, O1, and O2 lie strictly in the same plane. Ni-O bond lengths were 2.103(5) and 2.109(5) Å, and the Ni-N1 and Ni-N3 bond lengths were 2.070(11) and 2.050(6) Å, respectively. However, the N2-Ni-N2^i^ bond angle was significantly distorted from linear (158.3(2)°), and the Ni-N2/N2^i^ bond lengths were noticeably longer than the others (2.157(5) Å). Presumably this distortion from an octahedral set of ligand atoms was a consequence of the rigidity of the ligand and ethylene bridges of the cyclen. The two unbound chloride ions were disordered over three positions. These ions formed N-H∙∙∙Cl and O-H∙∙∙Cl hydrogen bonds in the solid. It is notable that Ni^2+^ prefers to bind H_2_O rather than Cl^−^, but this phenomenon has been observed for some cross-bridged tetraazamacrocycle Ni^2+^ chloride complexes as well [17]. 

**Figure 5:**
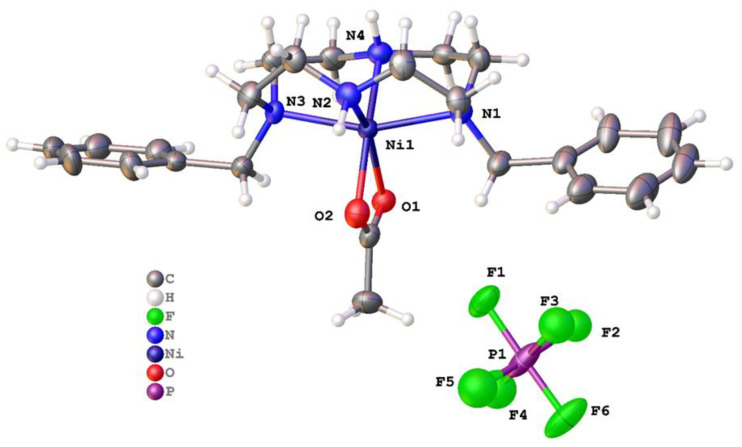
Molecular structure of [Ni(**1**)(μ-OOCCH_3_)]PF_6_. Atoms are drawn as 50% probability ellipsoids. For clarity, disorder is not represented.

Very similar coordination was observed in [Ni(**1**)(μ-OOCCH_3_)]PF_6_ [54], where ligand **1** bound to the metal adopting a V-shape, and the two remaining coordination sites were filled by chelating acetate (Figure 5). The C-O and Ni-O bond lengths suggest that this is very close to a symmetric chelate. The acetate O-Ni-O bite angle was 62.95(17)°, but even with this small bidentate ligand, the Ni ion still resided above the plane of the four nitrogen atoms of **1**, with the N1-Ni1-N3 bond angle being 160.0(5)°. The bond lengths were comparable to [Ni(**1**)(OH_2_)_2_]Cl_2_; Ni-N1 and Ni-N3 were much longer than the other bonds at Ni1 and had lengths of 2.162(12) and 2.154(13) Å, respectively. Additionally, there were unbound PF_6_^−^ anions in the structure. These resided between the metal complexes and formed N-H∙∙∙F and C-H∙∙∙F interactions.

**Figure 6:**
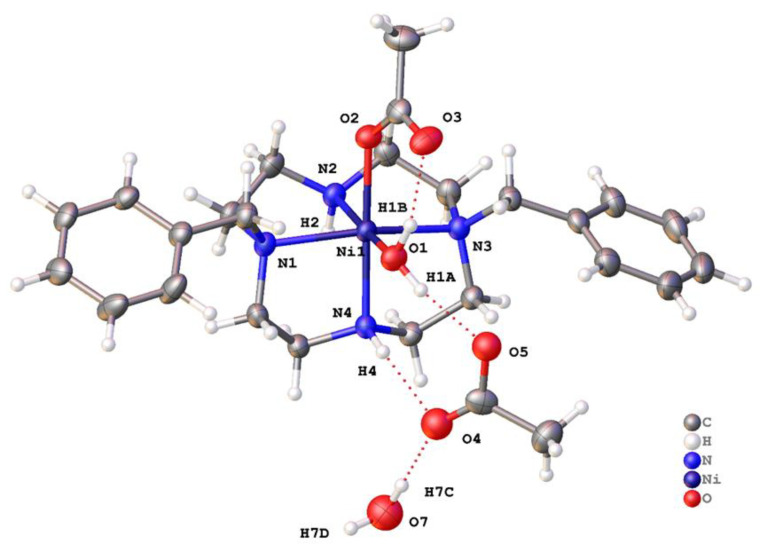
Asymmetric unit of [Ni(**1**)(OOCCH_3_)(OH_2_)](OOCCH_3_)∙H_2_O with atoms drawn as 50% probability ellipsoids. Dashed lines show classical hydrogen bonds.

[Ni(**1**)(OOCCH_3_)(OH_2_)](OOCCH_3_)∙H_2_O crystallized in the centric space group *P*2_1_/*n* with a single complex in the asymmetric unit (Figure 6). The coordination of the Ni^2+^ ion in [Ni(**1**)(OOCCH_3_)(OH_2_)](OOCCH_3_)∙H_2_O was very similar to the other Ni examples. Ligand **1** was in the same V-shape coordination mode; the two remaining coordination sites were occupied by monodentate acetate and water. As in the other examples, Ni-N1 and Ni-N3 bonds were 0.1 Å longer than each of the other coordination bonds to nickel. The N1-Ni1-N3 angle was 160.26(7)°. The bound water formed an intramolecular hydrogen bond to the acetate ligand, and a second hydrogen bound to the unbound acetate. N-H∙∙∙O and HO-H∙∙∙O hydrogen bonds were also formed to acetate. There was further hydrogen bonding between the N-H groups of **1** and acetate. The classical hydrogen bonds formed tapes of complexes parallel to the crystallographic b axis. Between these tapes were C-H∙∙∙*π* and C-H∙∙∙O interactions.

**Figure 7:**
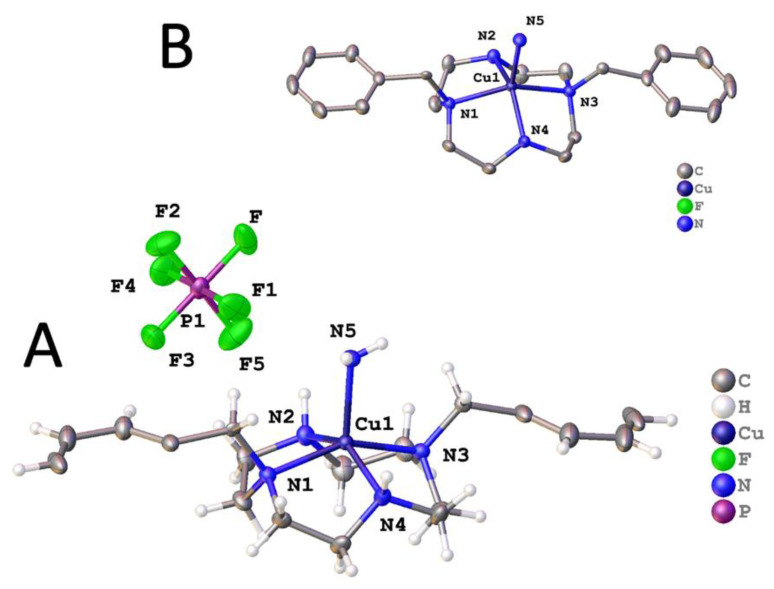
(**A**) Asymmetric unit of [Cu(**1**)(NH_3_)](PF_6_)_2_ with atoms drawn as 50% probability ellipsoids. Other parts of the molecule are generated by the mirror plane. (**B**) Coordination about Cu(**1**) within the whole molecule. For clarity, hydrogen atoms and disorder are not shown.

The coordination geometry in the copper complex [Cu(**1**)(NH_3_)](PF_6_)_2_ was understandably different from the nickel examples. This complex crystallized in the centric space group *P*2_1_/*m* with the molecule residing on the mirror plane. There was disorder over this mirror plane in the cyclen part of the molecule only; the two benzyl groups lie such that the action of the mirror plane completed these groups rather than replicating them. The two disorder components were related by rotation of 13.3°. There are further figures to illustrate this in the SI. The asymmetric unit of [Cu(**1**)(NH_3_)]PF_6_ is shown in Figure 7.

In this copper complex, ligand **1** adopted a flatter geometry than for the nickel examples, and the spatial orientation of the ligand donor atoms was different. The four nitrogen atoms lied in a square plane, and the copper ion was displaced above this plane; the fifth coordination site was occupied by ammonia to form a distorted square pyramidal coordination geometry about the metal. In the nickel complexes above, one N_ax_-M-N_ax_ bond angle was clearly much larger than the others, defining the axial positions of a distorted octahedron. It was always the benzylated nitrogens in these axial positions. Uniquely, in [Cu(**1**)(NH_3_)](PF_6_)_2_, the benzylated N2-Cu-N4 angle, as dictated by the much more square pyramidal geometry, and N1-Cu-N3 angles were nearly the same; they were 145.81(8)° and 154.74(7)°, respectively. This structural difference will be noted below in the kinetic stability section. The Cu-N bond lengths to the ligand did not show the same deviation as for nickel, but Cu-N1 and Cu-N3 were still longer (2.0799(18) and 2.0724(18) Å, respectively) than Cu-N2 and Cu-N4 (2.037(5) and 2.029(5) Å, respectively). The ammonia molecule formed N-H∙∙∙F interactions, and this is perhaps a contributing factor in the binding of ammonia rather than water. It is worth stating that it is clear from the diffraction data that ammonia was present and not water; the fit with water is appreciably worse.

**Figure 8:**
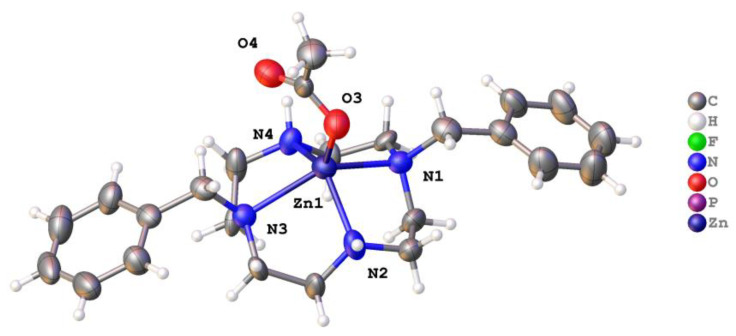
Molecular structure of [Zn(**1**)(OOCCH_3_)]PF_6_. Atoms are drawn as 50% probability ellipsoids. For clarity, disorder is not represented.

The zinc complex of **1**, [Zn(**1**)(OOCCH_3_)]PF_6_, also featured a five-coordinate metal in square pyramidal geometry crystallized in space group *P*2_1_ with one unique complex in the asymmetric unit. There was no evidence for a centre of symmetry. There was small-scale disorder in ligand **1,** but a satisfactory final refinement was possible (wR_2_ (all data) = 0.1112). The disorder was treated conservatively using standard techniques. The complex is shown in Figure 8.

The zinc ion is five coordinate in a coordination geometry most easily described as square pyramidal, as was the copper ligand **1** complex just described. The disorder present in the ligand set makes detailed calculations involving tau-5 of little value. The Zn lied around 0.825 Å above the plane of the four nitrogen atoms and bound a strictly monodentate acetate ion. The benzyl arms were angled downwards away from the bound acetate. There were N–H∙∙∙O(acetate) hydrogen bonds between adjacent complexes, and these formed infinite chains that ran parallel to the c-axis. There were further N–H∙∙∙F interactions between the complexes and PF_6_^−^ anions, and these were reinforced by C–H∙∙∙F interactions made with the aromatic ring. 

#### 2.2.2. Complexes of **2**, Dibenzylcyclam

**Figure 9:**
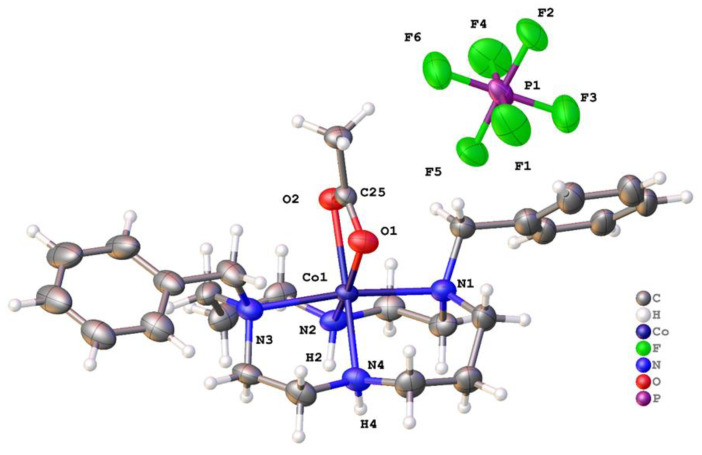
Molecular structure of [Co(**2**)(OOCCH_3_)]PF_6_. Atoms are drawn as 50% probability ellipsoids. For clarity, minor disorder is not represented.

[Co(**2**)(μ-OOCCH_3_)]PF_6_ crystallized in the centrosymmetric space group *C*2/*c* with a single complex in the asymmetric unit (Figure 9). There was small-scale disorder in the position of one of the phenyl rings, and this was treated using standard techniques. The cobalt ion was in a six-coordinate octahedral geometry; the ligand **2** adopted a cis-V configuration, and the final two coordination sites at the Co^2+^ ion were occupied by the chelating acetate. The Co-O (2.141(2) and 2.127(2) Å) and C-O (1.256(4) and 1.273(4)Å) bond lengths suggest the acetate acted as a symmetric bidentate ligand, and it had a bite angle at the metal of 61.53(9)°. The two Co-N bonds in the same plane as the acetate (Co1–N2 and Co1–N4 were 2.099(3) and 2.115(3) Å, respectively) were shorter than the two other Co-N bonds, which were 2.252(3) Å (Co1–N1) and 2.256(3) Å (Co1–N3). As expected, the N-Co-N angles for the ethylene bridges (~82°) were smaller than those of the propylene bridges (~92°). The two phenyl groups were arranged so that the two CH_2_–C_5_H_6_ bonds were almost co-linear, but the phenyl groups were tilted so that their mean planes were inclined at 66.6°.

Between the cobalt complexes and the PF_6_^−^ anions, there were N–H···F hydrogen bonds and further subsidiary C–H···F interactions, which may be crucial in determining the crystal packing. However, there were also fairly close C–H approaches between CH_2_ groups in adjacent complexes, and these may be destabilising. In the crystal structure, the complexes were arranged in sheets parallel to the xy-plane, and the anions lied between these planes. 

**Figure 10:**
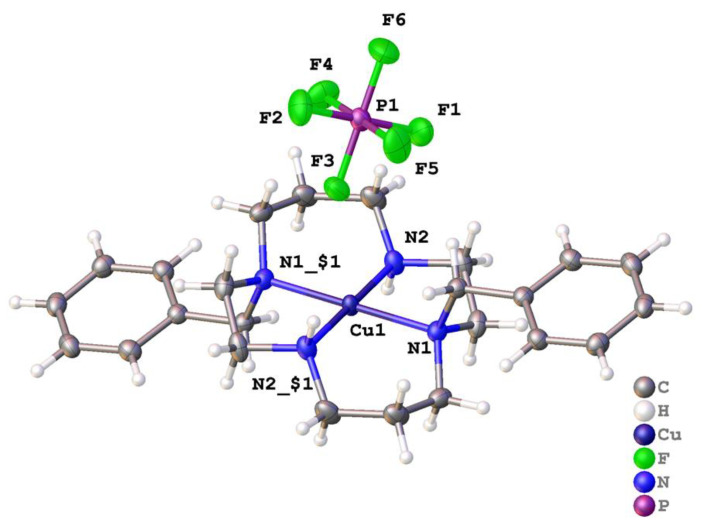
Coordination about the metal ion in [Cu(**2**)](PF_6_)_2_. Atoms are drawn as 50% probability ellipsoids. Symmetry operation for generating equivalent atoms: $1 = 1 − *x*, 1 − *y*, 1 − *z*.

[Cu(**2**)](PF_6_)_2_ crystallized in the space group *P*-1 with the copper on the inversion center. Here, the copper coordination is best described as a regular square plane; Cu–N bond lengths were 2.0129(12) and 2.0129(12) Å, and the N–Cu–N bond angles were 86.58(5)° and 93.42(5)°. The structure featured two additional unbound PF_6_^−^ anions for each copper, located above and below the square plane, but each was oriented within the structure so that the Cu1···F3 distance was 3.18721(14) Å. This suggests that there was some sort of very weak interaction between the d^9^ Cu^2+^ ion and the two axial fluorine atoms, which is consistent with a rather extreme Jahn–Teller distortion (Figure 10). Each N–H formed a hydrogen bond to PF_6_^−^, and there were many additional C–H···F interactions between the ligand and fluorine atoms.

**Figure 11:**
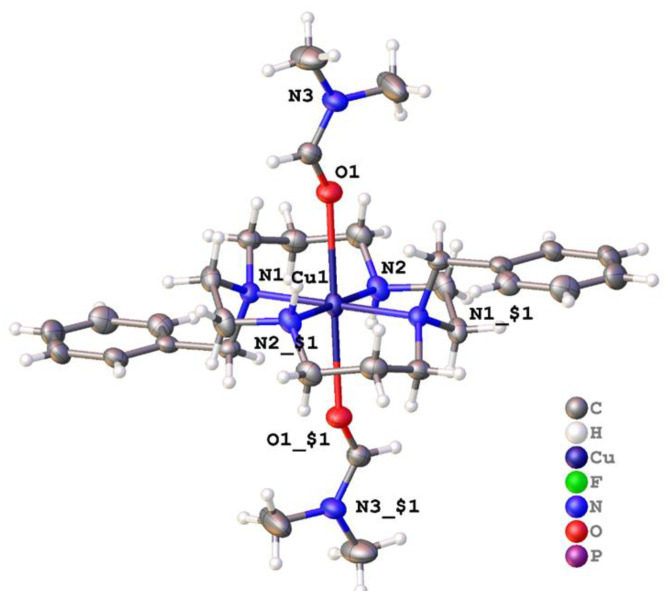
Coordination about the metal ion in [Cu(**2**)(OCHNMe_2_)_2_](PF_6_)_2_ · 2(OCHNMe_2_) [57]. Atoms are drawn as 50% probability ellipsoids. For clarity, unbound solvent and anions are not shown. Symmetry operation for generating equivalent atoms: $1 = 1 − *x*, −*y*, −*z*.

Alternative conditions yielded [Cu(**2**)(OCHNMe_2_)_2_](PF_6_)_2_ · 2(OCHNMe_2_) which crystallized in the centrosymmetric space group *P*2_1_/*n* with one half a complex in the asymmetric unit and the six-coordinate copper ion located on the inversion center such that the copper and four nitrogen atoms of **2** were strictly coplanar [54]. The Cu–N bond lengths were 2.004(2) Å and 2.0883(18) Å, but in line with the Jahn-Teller distortion expected for the d^9^ Cu^2+^ ion, the Cu–O bond lengths were much longer at 2.5783(16) Å. The coordination about the metal is shown in Figure 11. For each complex, there were two PF_6_^−^ anions that did not interact with the metal but instead formed C–H···F hydrogen bonds to surrounding complexes. The unbound dimethylformamide acted as a hydrogen bond acceptor to the N–H of the ligand and also formed one C–H(sp^2^)···F hydrogen bond to PF_6_^−^ to form a dense 3-D network of interactions extending in the solid. Adjacent complexes were arranged in tapes that ran parallel to the [011] direction; between adjacent molecules there were pi-pi interactions characterised by strictly planar phenyl rings at a separation of around 3.25 Å with slightly displaced centroids. 

The final structure is based on **2,** but this ligand underwent a reaction to generate **2′** as the ligand bound to Ni^2+^. The crystals were grown from [Ni(**2**)(OAc)](PF_6_), where no protection from air was provided. Two separate crystallization conditions, both containing diethyl ether, resulted in **2′** being formed. No other evidence of this modification of **2** was seen in other characterization techniques, and this appears to be a known phenomenon that can result from copper tetraazamacrocycle complexes usually modifying their own ligands to form the N(macrocycle)-CH_2_OCH_3_ pendant arm [55,61,62,63]. According to reference (Jeong et al.) [61] and reference (Alves et al.) [55], when aminal (N-CH_2_-N) derivatives of tetraazamacrocycles are reacted with MeOH in the presence of M^2+^ cations, the N-CH_2_OCH_3_ pendant arm can be formed. However, they were unsuccessful at intentionally isolating the ligand, as it appears to be easily hydrolyzed back to the amine. We saw no evidence of aminal formation, which generally requires formaldehyde and an uncoordinated macrocycle to take place. Perhaps in our case, two MeOH molecules were able to condense at the coordinated secondary amine and form the N-CH_2_OCH_3_ pendant arm while the secondary amine was coordinated to the activating Ni^2+^ cation. We did not investigate this reactivity any further. The hydrogen atom positions of the dimethyl ether appendage were clear from the difference Fourier maps and bond lengths were consistent with the atom type assignments.

**Figure 12:**
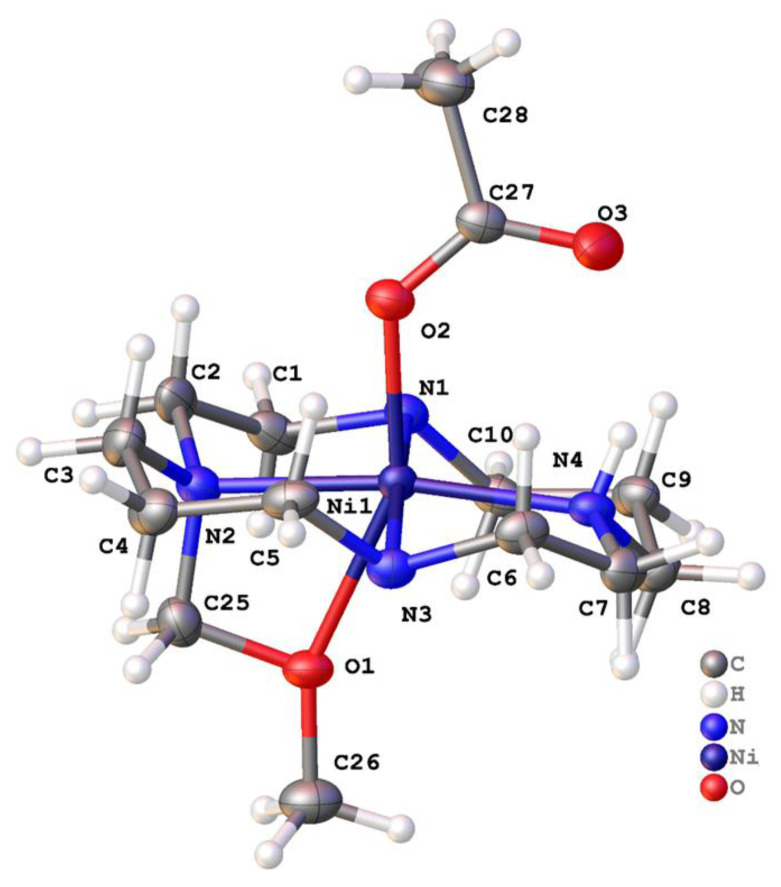
Structure of **2′**, the -CH_2_OCH_3_ modified version of **2**, which occurred spontaneously during the recrystallization of [Ni(**2**)(OAc)]PF_6_ from MeCN. Coordination about the metal in [Ni(**2′**)(OAc)]^+^ cation. For clarity, the benzyl groups attached to N1 and N3 are not shown.

**Figure 13:**
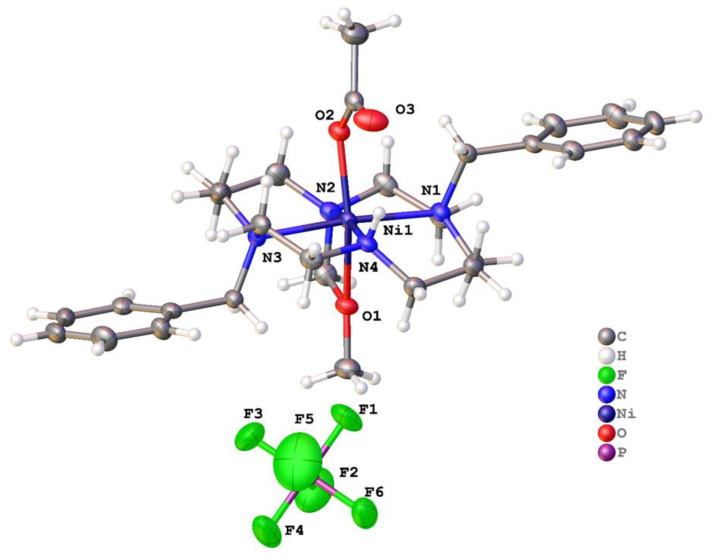
Asymmetric unit of [Ni(**2′**)(OAc)](PF_6_) · MeCN with atoms drawn as 50% probability ellipsoids.

[Ni(**2′**)(OAc)](PF_6_) · MeCN crystallized in the centrosymmetric space group *P*2_1_/*n* with a single Ni^2+^ ion in the asymmetric unit (Figure 12 and Figure 13). The nickel adopted a rather distorted octahedral coordination geometry, with the four nitrogen atoms of the ligand and the oxygen of monodentate acetate forming the vertices of an undistorted square pyramid. However, the -CH_2_OCH_3_ pendant was not sufficiently long for the oxygen to occupy the sixth coordination site. Instead, it was pushed toward the plane of the four nitrogen atoms. The bite angle subtended at the metal by the pendant ether and the attached nitrogen was 62.30(9)°, and the Ni1-O1 bond length was unusually long (2.306(2) Å), indicating that this was a rather unusual coordination mode. Five similar structures (REFCODES CAHNUW, CAHPAE, CIYBAP, QEYMOX, and SUSTEI) featuring a pendant ether arm with a methylene link are found in the Cambridge Structural Database (CSD). Each features a copper cation and a macrocyclic ligand on which the pendant arm(s) are sited. The Cu–N bond lengths were similar to those for [Ni(**2**′)(OAc)](PF_6_).MeCN, but each of the Cu–O bonds was markedly longer, with a median length of 2.49 Å. Aside from the unusual pendant, the ligand coordination was similar to that in the [Cu(**2**)(OCHNMe_2_)_2_]^2+^ ion. Disordered solvent was modelled using a solvent mask within Olex2, and the electron density count was consistent with one acetonitrile molecule per metal ion.

There was a single classical hydrogen bond: between N4-H4 and O3. Between adjacent species, there were C–H···O and C–H···F hydrogen bonds. In the solid state, the complexes were arranged in double columns parallel to the crystallographic a direction. These pairs of columns adopted a checkerboard arrangement in the yz plane. Between them lied the PF_6_^−^ anions and small channels (parallel to a) in which the disordered solvent was located.

Table 2 combines relevant structural parameters for the X-ray crystal structures of the transition metal complexes of **1** and **2** described above with previously published matching parameters for ethylene-cross-bridged cyclam and cyclen ligands for comparison. Where possible, comparisons were made with **Bn_2_Bcyclen** and **Bn_2_Bcyclam** as the closest structural analogues of **1** and **2**, but in some cases additional **Me_2_Bcyclen** and **Me_2_Bcyclam** complexes were included for comparison.

**Table 2:** N_ax_-M-N_ax_ and N_eq_-M-N_eq_ bond angles (**°**) for bridged and unbridged cyclen and cyclam complexes for comparison.

Complex	N_ax_-M-N_ax_ Angle	N_e__q_-M-N_e__q_ Angle	X-M-X Angle	X Ligands	Ref
[Ni(**1**)(OH_2_)_2_]^2+^	158.3(2)	92.7(4) 110.4(6)	83.6(2)	H_2_O, H_2_O	This work
[Ni(**1**)(OAc)]^+^	160.0(5)	102.6(4)	62.95(17)	O—C—O (acetate)	[54] This work
[Ni(**1**)(OAc)(H_2_O)]^+^	160.26(7)	97.04(7)	88.03(6)	H_2_O, O (acetate)	This work
[Ni(**Bn_2_Bcyclen**)(OAc)(H_2_O)]^+^	163.52(6)	85.59(7)	87.61(6)	H_2_O, O (acetate)	[29]
[Ni(**Bn_2_Bcyclen**)Cl_2_]	158.40(7)	83.37(9)	90.18(3)	Cl, Cl	[29]

[Cu(**1**)(NH_3_)]^2+^	150.74(7)	145.81(8)	n/a (5-coord)	NH_3_	This work
[(Cu(**Bn_2_Bcyclen**))_2_(μ-CO_3_)]	155.65	86.50	66.37	O—C—O	[64]
[Cu(**Me_2_Bcyclen**)(CO_3_)]	154.88(7)	85.17(7)	66.94(6)	O—C—O (carbonate)	[28]
[Cu(**Me_2_Bcyclen**)(OAc)]^+^	164.04(8)	85.00(8)	n/a (5-coord)	O (acetate)	[28]

[Zn(**1**)(OAc)]^+^	138.4(3)	132.4(4) 126.2(12)	n/a (5-coord)	O (acetate)	This work Note: only one of the two similar independent molecules in the unit cell is presented here
[Zn(**2**)(Cl)]^+^	159.6(1)	120.9(1)	n/a (5-coord)	Cl	[55]
[Zn(**Bn_2_Bcyclen**)Cl_2_]	152.61	77.62	95.50	Cl, Cl	[65]
[Zn(**Me_2_Bcyclen**)(OAc)(H_2_O)]^+^	157.59(10)	81.80(10)	90.83(8)	H_2_O, O (acetate)	[28]

[Co(**Me_2_Cyclen**)(CO_3_)]^+^	167.4(6)	101.7(3)	68.6(2)	O—C—O (carbonate)	[66]
[Co(**2**)(OOCCH_3_)]^+^	171.54(10)	97.15(11)	61.53(9)	O—C—O (acetate)	This work
[Co(**Bn_2_Bcyclam**)Cl_2_]	167.7(4)	82.5(4)	93.27(13)	Cl, Cl	[29]
[Co(**Me_2_Bcyclam**)Cl_2_]	172.4(2)	81.11(13)	97.37(4)	Cl, Cl	[67]

[Cu(**2**)]^2+^	180.00(10)	180	n/a (4-coord)	None	This work
[Cu(**2**)(DMF)_2_]^2+^	180.00	180	180.00(9) *trans*	DMF, DMF	[54]
[Cu(**Bn_2_Bcyclam**)(OAc)][OAc]	176.11(8)	86.05(9)	n/a (5-coord)	O (acetate)	[28]
[Cu(**Bn_2_Bcyclam**)(OAc)][PF_6_]	176.74(8)	85.93(8)	n/a (5-coord	O (acetate)	[28]

[Ni(**2′**)(OAc)]^+^	174.83(9)	170.21(10)	153.90(8) *trans*	O (acetate) -CH_2_OCH_3_	This work
[Ni(**Bn_2_Bcyclam**)Cl_2_]	170.75(15)	84.56(16)	92.00(5)	Cl, Cl	[29]
[Ni(**Bn_2_Bcyclam**)(OAc)(H_2_O)]^+^	163.52(6)	85.59(7)	87.61(6)	H_2_O, O (acetate)	[29]

Examination of the cyclen-based structures made evident that a major difference in structures between the bridged and unbridged ligands can be identified by examining the N_eq_-M-N_eq_ bond angles. This is the N-M-N bond angle containing two non-adjacent nitrogen atoms that would be considered on the equator of an octahedral structure. These are unsubstituted nitrogen atoms in the unbridged ligands and are the two nitrogens bridged by the ethylene cross-bridge in the bridged ligands. In ethylene-cross-bridged cases, this angle was restricted to 77.62–86.50°. This less than 90° restriction requires the bridged ligands to be tightly folded and bound *cis* on one side of the metal atom and is in great contrast to the unbridged ligand **1**, which displayed angles from 92.7(4) to 145.81(8)°. In the Cu(**1**) and Zn(**1**) structures, the complexes are most accurately described as square pyramidal, with the four ligand nitrogen atoms being nearly coplanar with the metal ion slightly displaced above the plane. However, cyclen-based **1** was too small to achieve a square-planar coordination geometry. The small size was most evident in the N_ax_-M-N_ax_ bond angles, which ranged only from 138.4(3) to 164.04(8)° for all cyclen ligands and no particular contrast is seen between bridged and unbridged ligands. We predicted that the flexibility and small size of **1** would lead to less kinetic stability than the cross-bridged ligand (see kinetic stability results below).

A similar trend was evident for the cyclam-based ligands, with the cross-bridged N_eq_-M-N_eq_ bond angles being restricted to 81.11(13)–86.05(9)°. However, the unbridged ligand **2** exhibited much larger N_eq_-M-N_eq_ bond angles, from 97.15(11) to 180°, and could support true square-planar coordination geometries. In square-planar as well as trans-octahedral cases, this larger ligand must also have 180° N_ax_-M-N_ax_ bonds, something the cross-bridged ligands cannot quite achieve, as they are restricted to 163.52(6)–176.74(8)°. The larger size of the cyclam-based cross-bridged ligands allows *cis* octahedral geometries that are not nearly as distorted as the smaller cyclen cross-bridged ligands, a fact that has been used to explain their much greater kinetic stability under harsh conditions [68,69]. 

Below, we use these geometric comparisons to explain the kinetic stability of ligand **1** and **2** in comparison with their cross-bridged analogues.

We considered the effect of functionalising these macrocycles on the M-N bond lengths. Given the diversity of metals here and the range of different coordination preferences, it is difficult to draw widely applicable conclusions. Given the preference for octahedral coordination and consistent oxidation state, we focused on cyclen complexes of Ni^2+^. It is clear that in complexes of **1** with Ni^2+^, M-NH coordination bonds were consistently around 5% shorter than M-N-benzyl bonds, as described earlier. We compared various similar complexes of nickel in the CCDC to examine the effect on the M-N bond length of an ethylene bridge and other substituents on the nitrogen. The raw data are presented in Table 3.

From 31 six-coordinate complexes, the mean M-N bond length for cyclen was 2.066 Å (σ = 0.025). There were eight further pertinent complexes in the CCDC that are identified by their CCDC number in the Table 3.

From examination of the eight examples in Table 3, it is clear that M-N bonds for the nitrogen atoms of an ethylene bridge are always the shortest M-N bonds present in the given structure. Presumably, this is a consequence of the strained five-membered chelate. In only one example was this not the case. The “bridge” M-N bonds were shorter than those of benzyl-substituted nitrogen, shorter than those of nitrogen atoms with methyl or other similar substituents, and they appeared in general to be shorter than M-NH bonds. Further analysis with a much broader range of metals is possible, but it is beyond the scope of the present work.

### 2.3. Kinetic Stability

Kinetic stability studies of copper complexes demonstrated their stability in harsh acidic media. Weisman et al. have standardized the conditions for acid decomplexation studies of metal complexes, which is usually performed for copper complexes. Such complexes have a single, moderately high extinction coefficient d-d band near 600 nm, meaning their decomplexation is readily observed by UV-Vis spectroscopy. Holding the metal ion (Cu^2+^) constant allows a comparison of the effects of various ligand architectural changes and amendments, such as macrocycle size and pendant arm identity, on kinetic stability. In this work, we determined the half-lives of copper complexes in highly acidic environments following pseudo-first-order kinetics. Table 4 lists the half-life values of the copper complexes at different temperatures and acid environments.

The unsubstituted bridged cyclen **H_2_Bcyclen** complex is not stable in acid; it has a half-life of <1 min at 30 °C and 1 M HCl. Substituting by two methyl groups enhanced its stability significantly, as the **Me_2_Bcyclen** complex had a half-life of 36 min under similar conditions. However, the **Me_2_Bcyclen** complex decomposed within a minute at stronger acidic conditions of 5 M HCl at 50 °C. Substitution of the **Me_2_Bcyclen** complex with two benzyl groups augmented its stability to about eight times as long a half-life; the **Bn_2_Bcyclen** complex had a half-life of 4.2 h. In general, cross-bridged ligands provide extra stability to the metal complex compared to their unbridged analogue due to enhanced topological and rigidity constraints [2,11,68,69,70]. Surprisingly, the unbridged cyclen complex with two benzyl substituents was even more stable than its cross-bridged analogue. The complex of **1** had a half-life almost double (8.47 h) that of the **Bn_2_Bcyclen** complex at 30 °C and 1 M HCl. To explain this surprising result, we invoke likely enhanced complementarity [3] of **1** for Cu^2+^ compared with **Bn_2_Bcyclen**. Complementarity is defined as a size, geometry, and electronics match between metal and ligand [3]. As previously noted, Cu(**1**) has a near-square pyramidal geometry which must make it more difficult for protons to react with the nitrogen donor atoms in order to remove the ligand entirely. In contrast, Cu(**Bn_2_Bcyclen**) is folded into a cis geometry to one side of the Cu^2+^ ion, which, regardless of the enhanced rigidity and topological complexity provided by the ethylene cross-bridge, clearly does not prevent protons from decomplexing the ligand as efficiently as **1**. In fact, the accepted mechanism of acid-caused ligand dissociation of tetraazamacrocycles requires folding to a cis geometry in order for the initial nitrogen donor to dissociate and become protonated [3]. In the case of Cu(**1**), the folding has not yet occurred, which slows down the dissociation, while Cu(**Bn_2_Bcyclen**) is already folded. This is a good lesson to learn, particularly in the case of smaller cyclens; the ethylene cross-bridge may actually be a detriment to kinetic stability by making the ligand a poor fit for Cu^2+^, especially if a more complementary unbridged analogue such as **1** is used.

The **H_2_Bcyclam** complex had better stability than the **H_2_Bcyclen** complex, as the larger ligand could now more easily and complementarily accommodate the Cu^2+^ ion in a less-strained square-pyramidal coordination geometry. The **H_2_Bcyclam** complex had a half-life of 11.8 min at 90 °C in 5 M HCl. Substitution with methyl groups improved its stability exceptionally, as **Me_2_Bcyclam** had a half-life of 7.3 days and 79 min at 5 M HCl at 50 °C and 90 °C, respectively. The Me substituents took the place of N-H’s which are known to be exchangeable with protons, thus improving the complex stability. However, substitution with two benzyl groups did not contribute to enhanced kinetic stability. The **Bn_2_Bcyclam** complex had a half-life of only 2.38 h and 24 min at 5 M HCl at 50 °C and 70 °C, respectively, which was considerably less than the **Me_2_Bcyclam** ligand complex. Perhaps the steric bulk of the two benzyl arms contributed to the ease of ligand decomplexation in this case, even as the Bn groups slightly enhanced the stability of **Bn_2_Bcyclen** compared to **Me_2_Bcyclen**. Clearly, the prediction of Cu^2+^ complex stability is not simply based on pendant arm and cross-bridge presence. We plan to study additional examples of steric bulk as a parameter in future work. The unbridged analogue **2** complex exhibited a half-life of only 33 min at 50 °C with 5 M HCl. Thus, as expected, the unbridged **Bn_2_cyclam** was considerably less stable than its cross-bridged analogue. We believe that in this case, where ligand size was much more complementary to Cu^2+^ in both the bridged and unbridged cases, the topological and rigidity constraints present in **Bn_2_Bcyclam** provided the expected additional kinetic stability, which did not occur in the case of the smaller, less complementary cyclen.

### 2.4. UV-Visible Spectroscopy

UV-Visible spectroscopic values for the 12 UV-Visible active complexes of ligands **1**, **2**, **Bn_2_Bcyclen**, and **Bn_2_Bcyclam** are given in Table 5. Zinc complexes are not UV-Visible active due to their d^10^ electron configurations, and they were therefore not included. “λ_max_” indicates peak locations (wavelength) and “ε” (extinction coefficient) indicates intensity of light absorption. All spectra were recorded in acetonitrile at similar concentrations.

**Figure 14:**
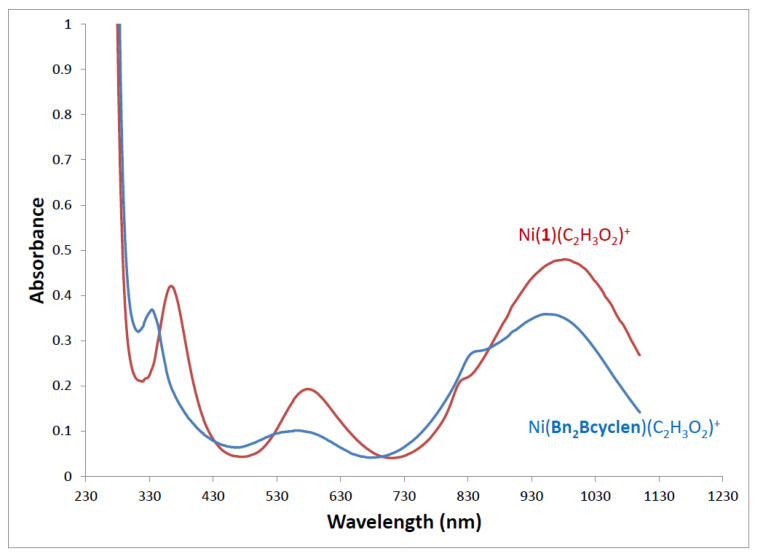
UV-Visible spectra of Ni(**Bn_2_Bcyclen**) and Ni(**1**) in acetonitrile at 0.010 M.

It is striking how similar the absorbance wavelengths and intensities are when comparing complexes that differ only in the presence or absence of the cross-bridge. In most cases, wavelengths were within 10–30 nm, and extinction coefficients were within 10–50 M^−1^ cm^−1^ of each other. Figure 14 illustrates what a typical UV-Visible spectrum looks like and shows, as an example, how similar spectra for Ni(**Bn_2_Bcyclen**) and Ni(**1**) were. There were four absorbances at nearly the same wavelength and with nearly the same intensity for both complexes. This observation argues that the cross-bridge does not make a large difference in the electronic properties of the Ni^2+^ ion bound to the two ligands.

Two significantly different pairs were found in the copper complexes. In these cases, the cross-bridge was not the only difference in the structure. In cross-bridged Cu(**Bn_2_Bcyclen**) and Cu(**Bn_2_Bcyclam**), there was an additional acetate ligand (according to elemental analysis), and the ligand was necessarily folded (cis) as required by the cross-bridge, giving square-pyramidal coordination geometries. In the unbridged Cu(**1**), the acetate was present, but the crystal structure showed a much different ligand arrangement of nearly coplanar nitrogen atoms. Additionally, in Cu(**2**), as demonstrated in its crystal structures (Figure 13 and Figure 14) the ligand was square planar, with or without axial fifth and sixth donors, depending on the exact crystal. In the case of cyclen ligands, the d-d band was shifted from 728 nm for Cu(**Bn_2_Bcyclen**) to 607 nm for Cu(**1**). This large shift matched the large difference in structure. In the case of cyclam ligands, the d-d band was shifted from 728 nm for Cu(**Bn_2_Bcyclam**) to 528 nm for Cu(**2**), which again matched the large difference in structure.

Another difference was observed in the Co(**Bn_2_Bcyclen**) vs. Co(**1**) pair. While the wavelengths were similar, the extinction coefficients were quite different. The Co(**Bn_2_Bcyclen**) complex was determined to have oxidized upon workup in air to the Co^3+^ cation, likely to generate a better complementarity (size) match of the smaller Co^3+^ with the small cavity available within the **Bn_2_Bcyclen** ligand. The same aerobic workup of Co(**1**) did not oxidize its Co^2+^ ion, as the unbridged ligand (as suggested by the copper kinetics experiments) would likely be more complementary to the larger Co^2+^ ion. In comparison with other cobalt complexes [47,67] of similar azamacrocycle ligands, the wavelengths and extinction coefficients were consistent with these observations. Again, this made direct comparison of the effect of just the bridge impossible since other factors changed. The difference in ease of oxidation was likely present due to the distortion of the preferred octahedral geometry by the short cross-bridge. It forced the macrocyclic ligand to be folded tightly and likely reduced the size of the cavity for metal binding. Oxidation to Co^3+^ results in a smaller metal ion than Co^2+^, and it was therefore apparently favored by the bridged ligand in Co(**1**) over the more flexible unbridged ligand in the Co(**Bn_2_Bcyclen**) analogue. Interestingly, the Co(**Bn_2_Bcyclam**) and Co(**2**) pair both remained in the Co^2+^ oxidation state. These ligands are both 14-membered rings, which is two carbons larger than the 12-membered **Bn_2_Bcyclen** and **1** ligands. Both larger rings, bridged or not, appeared to accommodate larger Co^2+^ under the aerobic workup conditions in a complementary way, as suggested by the copper kinetic stability experiments above.

### 2.5. Cyclic Voltammetry

Exploring the oxidation/reduction chemistry of these complexes can be done directly through cyclic voltammetry experiments. In these experiments, the complexes in acetonitrile solution are subjected to a sweeping change in electrochemical potential, which can result in oxidation and reduction of the original metal ion. E_1/2_ values (electrochemical potentials where oxidation/reduction occurs) and E_a_-E_c_ values (reflecting how reversible the oxidation/reduction pair is) for these complexes are given in Table 6. E_1/2_ values indicate coupled oxidation/reduction pairs that are assigned to the complex which appears to undergo little change (such as gain/loss of ligands) other than the gain/loss of electrons. In these cases, a small E_a_-E_c_ value indicates essentially no structural rearrangement upon oxidation/reduction, with 59 mV being the theoretical smallest value. Larger E_a_-E_c_ values indicate some structural changes that shift the partner event further away than the theoretical value. E_red_ or E_ox_ are used when single oxidation or reduction processes are observed but with no identifiable return process. 

**Figure 15:**
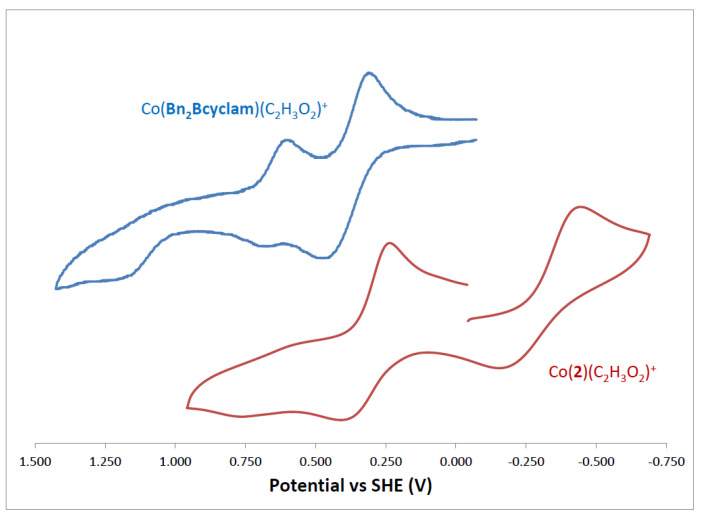
Cyclic Voltammograms of Co(**Bn_2_Bcyclam**) and Co(**2**) in acetonitrile at 0.001 M.

Although certain patterns of the quantity and types of redox processes for each metal were observed, many differences were seen between bridged and unbridged analogues. No bridged/unbridged pair gave nearly as similar a behavior as in the UV-Visible spectra discussed above. Figure 15 illustrates a typical cyclic voltammogram and also shows, as an example, differences in the voltammograms for Co(**Bn_2_Bcyclam**) and Co(**2**). Co(**Bn_2_Bcyclam**) had only oxidations, with two reversible processes around +0.500 V, both assigned as Co^2+/3+^ couples of possible coordination isomers (perhaps monodentate vs bidentate acetate), and an additional unassigned irreversible process near +1.200 V. Co(**2**). Moreover, it gave the same number of peaks, had one quasi-reversible reduction to Co^+^ in the negative region (near −0.300 V), and had only one reversible oxidation. Although the curves look similar in shape, the potential shifts were large, and the types of processes each complex undergoes were significantly different.

While it is possible to discuss each bridged/unbridged pair in turn, we did not derive any recognizable pattern since there were rather large changes in many cases that did not necessarily correlate between different bridged/unbridged pairs. Instead, we will speculate on why the electrochemical behavior was so different when the bridge was removed, while the UV-Visible spectra changed so little. A primary reason is likely the static nature of the UV-Visible spectrum versus the dynamic reactivity inherent to cyclic voltammetry. The UV-Visible spectrum is obtained on a complex without causing it to change in any way; thus, there is only information on the unreacted complex as it exists in a single structure in solution. According to the highly congruent UV-Visible data, the initial bridged and unbridged complexes had electronic structures that are very similar to one another. The presence or absence of the bridge made little difference except in the preference for Co^2+^/Co^3+^ in the case of Co(**Bn_2_Bcyclen**)/Co(**1**) and for the Cu complexes, where X-ray crystal structures showed very different cis-folded ligands for the cross-bridged ligands vs. square-planar ligand geometries for the unbridged ligands **1** and **2**. All of the cyclen complexes had very similar cis-folded ligand coordination, whether cross-bridged or not, and were thus similar in their UV-Vis spectra. 

However, the cyclic voltammetry experiment is a dynamic one, where complexes gain and lose d-electrons in response to the electrical potential they are subjected to. Once oxidation or reduction takes place, complexes may structurally rearrange in response to the new d-electron configuration or even gain or lose ligands due to the preference of the new metal ion oxidation state. It should not be surprising that the presence/absence of the two-carbon cross-bridge led to quite different structural rearrangements and/or ligand gains/losses, as demonstrated by the significant differences in electrochemical behavior between complexes differing only by the bridge. For example, the crystal structure of Co(**2**)(OAc)^+^ was determined (Figure 9) and consisted of a bidentate acetate and a cis-folded ligand giving an octahedral coordination geometry. The Co(**Bn_2_Bcyclam**)(OAc)^+^ structure was not determined. However, the Co(**Me_2_Bcyclam**)(OAc)^+^ structure was determined [29], and it is very similar in coordination geometry to Co(**2**)(OAc)^+^, suggesting that the dibenzyl complex would be similar. The differences in redox processes are not easily rationalized based on these highly similar static crystal structures. However, if dynamic changes occur following a redox process, particularly if that change involves the N_eq_-M-N_eq_ bond angle, which is highly constrained in the ligand **Bn_2_Bcyclam** but very flexible in the case of ligand **2**, then large differences in the dynamic electrochemical experiments can be (if not explained precisely) at least understood in terms of why they may be quite different from one another.

Behavior of the unbridged ligand complexes of **1** and **2** with the same metal ion was generally similar, as might be expected given that the ligands differ by only ring size. The Co(**2**)(C_2_H_3_O_2_)^+^ complex had one quasi-reversible oxidation at +0.322 V, likely a Co^2+/3+^ couple, and one quasi-reversible reduction at −0.301 V, likely a Co^2+/+^ couple. Interestingly, the cyclen analogue Co(**1**)(C_2_H_3_O_2_)^+^ had only an irreversible oxidation at +0.705 V, likely Co^2+/3+^, and an irreversible reduction at +0.043 V, likely Co^2+/+^. The difference in reversibility may be due to the ability of the larger cyclam ligand **2** to adjust to larger and smaller metal ions without large structural changes. The smaller, more rigid cyclen ligand **1** appears to require irreversible structural changes as the size of the metal ion changes.

A similar phenomenon was observed for the Ni complexes. The Ni(**2**)(C_2_H_3_O_2_)^+^ complex exhibited a reversible oxidation couple, likely Ni^2+/3+^ at +1.290 V. Its reduction at −1.320 V, likely to Ni^+^, was irreversible, however. The cyclen analogue Ni(**1**)(C_2_H_3_O_2_)^+^ had only an irreversible oxidation at +1.230 V, likely Ni^2+/3+^, and an irreversible reduction at −1.220 V, likely Ni^2+/+^. Again, the large cyclam ligand **2** provided a more reversible result than the smaller, more rigid cyclen ligand **1**.

Finally, the Cu complexes seem to reverse this trend. The only feature observed for the larger cyclam complex Cu(**2**)(CH_3_CN)^2+^ was a nearly irreversible reduction to Cu+ at −0.484 V. However the cyclen complex Cu(**1**)(CH_3_CN)^2+^ was more reversibly reduced at −0.470 V and even displayed an irreversible oxidation to Cu^3+^ at +1.280 V. Perhaps the small size of the cyclen ligand **1** favored the smaller Cu^3+^ ion enough for this oxidation to happen within the observable redox window of acetonitrile, while the large cyclam ligand **2** favored the larger Cu^2+^ ion and thus prevented the oxidation to Cu^3+^ in acetonitrile.

## 3. Materials and Methods

### 3.1. General

*N,N*′-bis(aminopropyl)ethylenediamine (98%) was purchased from Acros Organics. Glyoxal (40% wt in water), benzyl bromide (99%), and sodium borohydride (98%) were purchased from Aldrich Chemical Co. (St. Louis, MO, USA). Cyclen was purchased from Strem Chemical Co. (Newburyport, MA, USA). All solvents were of reagent grade and were dried, when necessary, by accepted procedures. Cyclam was prepared according to a modified literature method from *N,N’*-bis(aminopropyl)ethylenediamine [72]. Elemental analyses were performed by Quantitative Technologies Inc. Electrospray (Whitehouse, NJ, USA). Mass spectra were collected on a Shimadzu LCMS-2020 instrument (Shimadzu, Kyoto, Japan). NMR spectra were obtained on a Varian Bruker AVANCE II 300 MHz NMR spectrometer instrument (Varian Bruker, Billerica, MA, USA). Electronic spectra were recorded using a Beckman Coulter DU800 UV-Vis Spectrometer (Beckman Coulter, Brea, CA, USA). Electrochemical experiments were performed on a BAS Epsilon EC-USB Electrochemical Analyzer (BASi, West Lafayette, IN, USA). A button Pt electrode was used as the working electrode with a Pt-wire counter electrode and a Ag-wire pseudo-reference electrode. Scans were taken at 200 mV/s. Acetonitrile solutions of the complexes (1 mM) with tetrabutylammonium hexafluorophosphate (0.1 M) as a supporting electrolyte were used. The measured potentials were referenced to SHE using ferrocene (+0.400 V versus SHE) as an internal standard. All electrochemical measurements were carried out under N_2_.

### 3.2. Acid Decomplexation Studies

This experiment has become a standard method for determining kinetic stability to compare new cross-bridged ligands. In order to determine their kinetic stability, the d-d absorption band (generally near 600 nm) of the copper complexes (1 mM) in highly concentrated acidic solutions (generally 5 M HCl) [8] was followed on a Shimadzu UV-3600 UV–vis–near-IR spectrophotometer (Shimadzu, Kyoto, Japan) at increasing temperatures until conditions were found that decomposed the complex in a matter of hours/days. The reaction rate patterns followed pseudo-first-order kinetics as expected. Half-lives of the metal complexes were calculated from the slope of ln absorbance vs. time plots.

### 3.3. X-ray Crystallography Studies

X-ray scattering data from crystals of the samples were collected using synchrotron radiation (λ = 0.7288 Å) coupled with a Bruker APEX-II diffractometer (Bruker AXS Inc., Madison, WI, USA) with a PHOTON-II detector. For three samples, the structures were determined using Mo Kα radiation. For a single twinned sample, data were collected using Cu Kα radiation. Data were scaled and merged, and a multi-scan absorption correction was applied. Structures were solved using dual-space methods in SHELXT. Structures were refined using SHELXL-2018 [73,74] implemented within Olex2 [75]. All non-hydrogen atoms were refined using anisotropic displacement parameters. Hydrogen atoms were placed at calculated positions using a riding model. CCDC 1555223, 2223999-2224003, and 2224005-2224007 contain the supplementary crystallographic data for this paper. These data can be obtained free of charge from The Cambridge Crystallographic Data Centre via www.ccdc.cam.ac.uk/data_request/cif.

### 3.4. Synthesis

**Figure 16:**
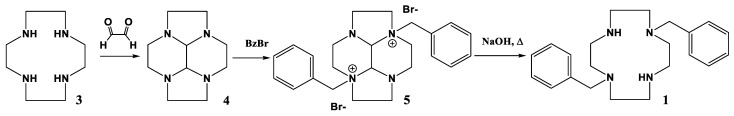
Synthetic scheme for 1,7-dibenzylcyclen (**1**).

Tetracyclen (**4**) [76] (Figure 16): 26.3 g (0.153 mol) of cyclen (**3**) and 105 mL of acetonitrile were added to a 500 mL roundbottom flask, which was then flushed for 15 min with N_2_ gas. Next, 22 mL (8.88 g or 0.153 mol) of 40% by mass glyoxal solution was added, and the reaction was stirred under N_2_ at 50–65 °C for 2 h. The solvent was removed, and the brown residue was extracted with 5 × 50 mL portions of chloroform. Following filtration, the chloroform solution was evaporated to give the product as an oil. The product was purified by column chromatography using neutral Brockman I alumina with 1% MeOH in CH_2_Cl_2_ as the eluent. The yield was 22.327 g (75%). Electrospray mass spec: *m*/*z* at 195 = LH^+^.

Dibenzyltetracyclen (**5**): 10.53 g (0.0543 mol) of **4** was dissolved in 300 mL of dry acetonitrile and added to a 500 mL roundbottom flask. Next, 97 mL (0.8145 mol, 15 eq) of benzyl bromide was added, the flask stoppered, and it was then stirred at room temperature for four days. [CAUTION: benzyl bromide is an extreme lachrymator; use only in a chemical fume hood.] The white solid product was filtered on a fine glass frit and then washed with acetonitrile and then ethyl acetate to remove excess benzyl bromide. The solid was vacuum dried to give 25.7 g of pure product (88% yield). Electrospray mass spec: *m*/*z* = 455 (L − Br)^+^. Elemental analysis calcd for C_24_H_32_N_4_Br_2_: C 53.73, H 5.97, N 10.45; found C 53.52, H 6.00, N 10.30.

1,7-Dibenzylcyclen (**1**): 36.115 g (0.0673 mol) of **5** and 360 mL of 3 M aqueous NaOH were added to a 500 mL roundbottom flask. The flask was stirred and heated in an oil bath at 80 °C for three days under nitrogen. A yellow solution with an orange oil floating on top resulted, and this was cooled and extracted with five portions of 80 mL of CH_2_Cl_2_. The organic layers were combined, dried over MgSO_4_, filtered, and evaporated to give an orange foamy solid product (20.656 g, 87% yield). Electrospray mass spectrum: *m*/*z* = 353 (LH^+^). Elemental analysis calcd for C_22_H_32_N_4_ · 0.5H_2_O · 0.65CH_2_Cl_2_: C 65.28, H 8.30, N 13.44; found C 65.61, H 8.61, N 13.45.

**Figure 17:**
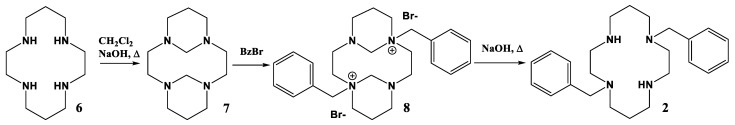
Synthetic scheme for 1,8-dibenzylcyclam (**2**).

Bis-methylene-bridged cyclam (BMBcyclam) (**7**) [77] (Figure 17): 12.0 g (0.060 mol) of cyclam (**6**) was added to a 2 L roundbottom flask and stirred with 600 mL of CH_2_Cl_2_ and 600 mL of 30% NaOH. This solution was then refluxed under a N_2_ atmosphere for 36 h. The biphasic solution was extracted four times with 100 mL CH_2_Cl_2_. The combined organic layer was dried over MgSO_4_ for one hour and was then filtered, evaporated, and dried under vacuum to obtain **7**. The yield was 12.25 g (91%). Electrospray mass spectrum: *m*/*z* = 225 (LH^+^). NMR (^1^H and ^13^C) gave peak regions of 2.17–3.10 ppm for macrocycle hydrogens and peaks at 19.4, 48.4, and 68.0 ppm for unique carbons, matching the literature reference [66].

DibenzylBMBcyclam (**8**): 12.0 g of **7** was dissolved in 250 mL of acetonitrile in a 500 mL roundbottom flask. Three equivalents of benzyl bromide were added and stoppered. This solution was stirred for one week at room temperature. The white solid produced was collected on a glass frit, washed with 50 mL of ethyl acetate to ensure all benzyl bromide was removed, and then dried under vacuum. The yield was 27.3 g (90%) Electrospray mass spectrum: *m*/*z* = 203 *m*/*z* (L − 2Br)^2+^, *m*/*z* = 407 (L − 2Br)^+^, and *m*/*z* = 487 (L − Br)^+^. NMR gave peak regions for macrocycle hydrogens at 1.76–3.60 ppm and benzyl hydrogens at 4.31–4.65 ppm. Unique carbons were seen at 19.4, 47.6, 51.3, 59.6, 62.9, and 76.8 ppm, matching the literature reference [66].

1,8-Dibenzylcyclam (**2**): 22.0 g of **8** was dissolved in 500 mL of 3 M NaOH in a 1 L Erlenmeyer flask. This solution was stirred for 3 h at room temperature. The solution was then extracted with five times 150 mL of CHCl_3_. All organic layers were collected and dried over MgSO_4_, after which they were filtered. The solution was evaporated and dried under vacuum to obtain **2**. The yield was 13.6 g (92%). Electrospray mass spec: *m*/*z* = 381 (LH). NMR (^1^H and ^13^C) analysis gave peak regions of 1.85, 2.51–2.74, and 3.71 ppm for macrocycle hydrogens and 4.72 ppm for benzyl hydrogens. Six unique carbon peaks were found at 26.0, 47.7, 50.2, 52.0, 54.2, and 58.2 ppm, matching the literature reference [66].

**Figure 18:**
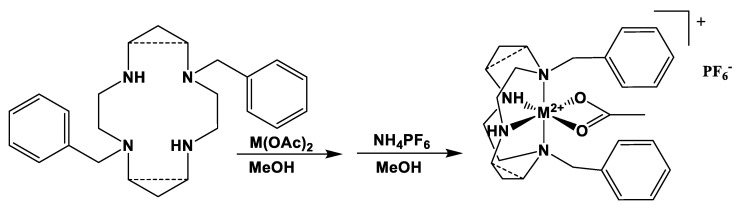
Metal complexation reactions.

Metal Complexation (Figure 18, Table 1 and Table 7): All complexation reactions were performed in an inert atmosphere glovebox. All complexations used one equivalent of anhydrous metal acetate (M(C_2_H_3_O_2_)_2_) salts in anhydrous methanol (20 mL) reacted with one equivalent of macrocyclic ligand. Complexations of **1** used 0.705 g (0.0020 mol) of ligand **1**; complexations of **2** used 0.425 g (0.0011 mol) of ligand **2**. The following specific example is typical of all eight complexation reactions.

A total of 0.425 g (0.0011 mol) of 1,8-dibenzylcyclam and 0.195 g (0.0011 mol) of anhydrous cobalt(II) acetate were added to a 20 mL reaction vial, and 15 mL of anhydrous methanol was added. The reaction was stirred at room temperature for seven days. The reaction vial was removed from the glovebox, and the workup was done in air. The reaction solution was filtered through Celite in a Pasteur pipette into a 100 mL roundbottom flask to remove any trace solids. Separately, five equivalents (0.0055 mol, 0.897 g) of NH_4_PF_6_ were dissolved in a minimal amount of methanol (~5 mL). This solution was filtered through a Kimwipe in a pipette and into the stirring metal complex solution. Precipitate of the pink complex as a PF_6_^−^ salt formed immediately. The reaction flask was placed in a freezer (−10 °C) for 1 h to complete the precipitation of the product. The solid pink powder product was collected on a fine glass frit and washed with a minimal amount of cold methanol followed by ether. The product was transferred to a 4-dram vial and dried overnight under vacuum. The yield was 0.506 g (70%).

[Note: one exception to the procedure above was required for the [Ni(Bn_2_Cyclen)(OAc)]PF_6_ complex. It did not precipitate from methanol. Therefore, it was evaporated to dryness and ~50 mL of water was added. The pale blue product was not water soluble and was filtered from the water solution.]

One chloride complex, [Ni(**1**)(OH_2_)_2_]Cl_2_ was initially synthesized, but poor solubility of the NiCl_2_ salt required refluxing DMF for complexation, which led us to abandon it in favor of the acetate salt for characterization studies where solubility was required. This complex did not give an entirely satisfactory elemental analysis, so its full characterization was not pursued. However, it did crystallize, so its X-ray crystal structure is included below for completeness. Calculated for [Ni(C_22_H_32_N_4_)(H_2_O)_2_]Cl_2_ · H_2_O: C 49.28, H 7.14, N 10.45; found: C 49.29, H 7.79, N 10.60.

## 4. Conclusions

Major findings from this study include that the crystal structures of the ligand **1** and **2** complexes exhibited much larger N_eq_-M-N_eq_ bond angles (up to 145.81°) as compared with the rigidly small N_eq_-M-N_eq_ bond angles enforced by the ethylene cross-bridge (77.62–86.05°). The N_ax_-M-N_ax_ bond angle differences between bridged and unbridged ligands were much smaller (only a ~26° range for cyclen complexes and ~13° range for cyclam complexes). The N_eq_-M-N_eq_ bond angle flexibility of the cyclen ligand **1** allowed vastly different coordination geometries than for **Bn_2_Bcyclen**, resulting in an enhanced complementarity for Cu^2+^ and a surprisingly more robust kinetic stability for the unbridged complex compared with the bridged version. When complementarity can be maintained in the bridged complex, such as in the larger **Bn_2_Bcyclam**, the topological and rigidity constraints brought to bear by the ethylene cross-bridge resulted in the expected increase in complex kinetic stability. Electronic characterization demonstrated that only when there are large differences in coordination geometry, as in *cis* vs *trans* ligand coordination of the macrocycle for **Bn_2_Bcyclam** vs. **2**, that a large difference in UV-Visible absorbance was observed. However, likely due to the dynamic process present in cyclic voltammetry experiments, larger differences in behavior were observed when comparing bridged versus unbridged ligand complexes.

## Figures and Tables

**Table 1:** Yields and selected peaks in the electrospray mass spectra of ligand **1** and **2** complexes.

Expected Complex	Color	Yield (g)	Yield (%)	*m*/*z*	*m*/*z*
[Co(**1**)(OAc)]PF_6_	pink-purple	0.506	71%	499 Co(**1**)(OAc)^+^	439 Co(**1**)^+^
[Ni(**1**)(OAc)]PF_6_	pale sky blue	0.324	46%	498 Ni(**1**)(OAc)^+^	219 Ni(**1**)^2+^
[Cu(**1**)](PF_6_)_2_	bright blue	0.291	37%	524 Cu(**1**)(OAc)(H_2_O)^+^	222 Cu(**1**)^2+^
[Zn(**1**)(OAc)]PF_6_	light tan	0.400	56%	505 Zn(**1**)(OAc)^+^	464 Zn(**1**)(H_2_O)^+^
[Co(**2**)(OAc)]PF_6_	pale pink	0.680	54%	470 Co(**2**)(OAc)^+^	410 Co(**2**)^+^
[Ni(**2**)(OAc)]PF_6_	pale sky blue	0.927	75%	469 Ni(**2**)(OAc)^+^	205 Ni(**2**)^2+^
[Cu(**2**)](PF_6_)_2_	brick red	1.055	72%	560 Cu(**2**)(PF_6_)^+^	208 Cu(**2**)^2+^
[Zn(**2**)(OAc)]PF_6_	off-white	0.945	76%	475 Zn(**2**)(OAc)^+^	436 Zn(**2**)(H_2_O)^+^

**Table 3:** N-M bond lengths (Å) for unbridged cyclen Ni^2+^ complexes for comparison. Identification of the complexes is given either by formula for complexes from this work or CCDC identifier.

	M-N Bond Length/Å			
Compound Identifier or CCDC Number	N-benzyl	N-CH_2_CH_2_-N Bridge	N-R	NH
[Ni(**1**)(OH_2_)_2_]Cl_2_	2.157(5)			2.070(11) 2.050(6)
[Ni(**1**)(μ-OOCCH_3_)]PF_6_	2.162(12) 2.154(13)			2.003(8) 2.060(8)
[Ni(**1**)(OOCCH_3_)(OH_2_)] (OOCCH_3_)∙H_2_O	2.1443(17) 2.1786(17)			2.0832(18) 2.0489(17)
1566343	2.227(3)	2.122(3)		
891843	2.206(3) 2.184(3)	2.100(3) 2.089(3)		
1975750		2.096(5)		2.088(5)
1101686		2.107 2.111		2.187 2.160
1101688		2.0921 2.1035		2.1509 2.1581
891844	2.135(3) 2.128(3)		2.177(3) 2.188(3) R = Me	
1891730		2.068(2), 2.083(2) 2.067(2), 2.079(2)	2.096(2), 2.082(2) 2.086(2), 2.094(2) R = CH_2_COO	
1891731		2.0696(17) 2.0709(17)	2.0841(17) 2.0970(16) R = CH_2_C(O)NH-Ar	

**Table 4:** Pseudo-first-order half-life for acid decomplexation of Cu^2+^ complexes under harsh conditions.

Ligand	30 °C, 1 M HCl	50 °C, 5 M HCl	70 °C, 5 M HCl	90 °C, 5 M HCl	References
* 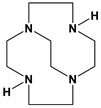 * H_2_Bcyclen	<1 min				[69]
* 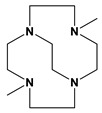 * Me_2_Bcyclen	36 min	<1 min			[70]
* 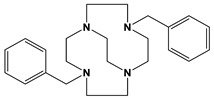 * Bn_2_Bcyclen	4.2 h	<1 min			[this work] [28]
* 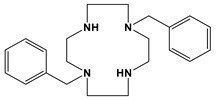 * Bn_2_cyclen = **1**	8.47 h	<1 min			[this work]
* 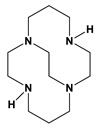 * H_2_Bcyclam				11.8 min	[8]
* 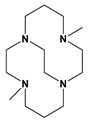 * Me_2_Bcyclam		7.3 day		79 min	[70,71]
* 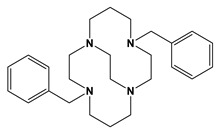 * Bn_2_Bcyclam		2.38 h	24 min		[this work] [28]
* 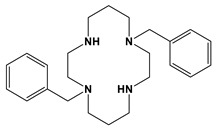 * Bn_2_cyclam = **2**		33 min			[this work]

**Table 5:** Electronic spectra comparison.

Complex	Metal Ion	λ_max_ in nm (ε in M^−1^ cm^−1^) [sh Indicates a Shoulder on Another Peak]				Ref
[Co(**Bn_2_Bcyclen**)(OAc)](PF_6_)_2_	Co^3+^	380 (235)	523 (356)	-----	-----	[29]
[Co(**1**)(OAc)]PF_6_	Co^2+^	372sh (50)	549 (58)	-----	-----	This work
						
[Co(**Bn_2_Bcyclam**)(OAc)]PF_6_	Co^2+^	464sh (17)	510 (20)	547sh (15)	-----	[29]
[Co(**2**)(OAc)]PF_6_	Co^2+^	----	513 (32)	552sh (23)	685 (6)	This work
						
[Ni(**Bn_2_Bcyclen**)(OAc)]PF_6_	Ni^2+^	334 (37)	559 (10)	845sh (28)	951 (36)	[29]
[Ni(**1**)(OAc)]PF_6_	Ni^2+^	364 (42)	587 (19)	820sh (21)	985 (48)	This work
						
[Ni(**Bn_2_Bcyclam**)(OAc)]PF_6_	Ni^2+^	354 (15)	570 (7)	829sh (5)	979 (12)	[25,29]
[Ni(**2**)(OAc)]PF_6_	Ni^2+^	364 (22)	579 (20)	814sh (20)	980 (18)	This work
						
Cu(**Bn_2_Bcyclen**)(OAc)]PF_6_	Cu^2+^	306 (6490)	728 (140)	-----	-----	[28]
[Cu(**1**)(OAc)](PF_6_)	Cu^2+^	301 (7020)	607 (465)	-----	-----	This work
						
[Cu(**Bn_2_Bcyclam**)(OAc)]PF_6_	Cu^2+^	306 (6930)	708 (150)	-----	-----	[28]
Cu(**2**)](PF_6_)_2_	Cu^2+^	282 (8374)	528 (194)	-----	-----	This work

**Table 6:** Redox potentials (vs. SHE) with peak separations.

Complex		E_1/2_ (V) Co^3+^/Co^2+^	(E_a_-E_c_) mV	E_1/2_ (V) Co^2+^/Co^+^	(E_a_-E_c_) mV	Ref
Co(**Bn_2_Bcyclen**)(C_2_H_3_O_2_)^2+^		+0.014	109	−0.640	178	[29]
Co(**1**)(C_2_H_3_O_2_)^+^		+0.705 (ox only)	-----	+0.043 (red only)	-----	This work
						
	**E_ox_ (V) unassigned**	**Co^3+^/Co^2+^**	**(E_a_-E_c_) mV**	**E_1/2_ (V) #2**	**(E_a_-E_c_) mV**	
Co(**Bn_2_Bcyclam**)(C_2_H_3_O_2_)^+^	+1.226	+0.638 +0.392	75 167	-----	-----	[29]
Co(**2**)(C_2_H_3_O_2_)^+^	+0.754	+0.322	156	−0.301	266	This work
						
	**E_ox_ (V) Ni^2+^/Ni^3+^**	**E_1/2_ (V) Ni^2+^/Ni^3+^**	**(E_a_-E_c_) mV**	**E_red_ (V) Ni^2+^/Ni^+^**		
Ni(**Bn_2_Bcyclen**)(C_2_H_3_O_2_)^+^	+1.170	+1.117	106	-----		[29]
Ni(**1**)(C_2_H_3_O_2_)^+^	+1.230			−1.220		This work
						
Ni(**Bn_2_Bcyclam**)(C_2_H_3_O_2_)^+^	+1.255	-----	-----	-----		[29]
Ni(**2**)(C_2_H_3_O_2_)^+^	+1.290	-----	90	−1.320		This work
						
	**E_ox_ (Cu^2+/3+^) [V]**	**E_red_ (Cu^2+/+^) [V]**	**E_ox_ (^Cu+/2+^) [V]**			
Cu(**Bn_2_Bcyclen**)(C_2_H_3_O_2_)^+^	+1.465	−0.637	-----			[28]
Cu(**1**)(CH_3_CN)^2+^	+1.280	−0.470	−0.240			This work
						
Cu(**Bn_2_Bcyclam**)(OAc)^+^	+1.516	−0.641	−0.156			[28]
Cu(**2**)(CH_3_CN)^2+^	-----	−0.484	−0.208			This work

**Table 7:** Formulas and elemental analyses of ligand **1** and **2** complexes.

(M/L) Complex Formulation for Elemental Analysis	Calc C	Calc H	Calc N	Found C	Found H	Found N
(Co/1) [Co(C_22_H_32_N_4_)(C_2_H_3_O_2_)]PF_6_	45.50	5.89	8.84	45.12	5.52	8.65
(Ni/1) [Ni(C_22_H_32_N_4_)(C_2_H_3_O_2_)]PF_6_ · 1.0 H_2_O	45.52	5.89	8.85	45.52	5.89	8.85
(Cu/1) [Cu(C_22_H_32_N_4_)(C_2_H_3_O_2_)]PF_6_ · 0.8 NH_4_PF_6_	38.41	5.13	8.96	38.60	5.01	9.18
(Zn/1) [Zn(C_22_H_32_N_4_)(C_2_H_3_O_2_)]PF_6_ · 0.5 H_2_O	45.69	5.75	8.88	45.71	5.39	8.97
(Co/2) [Co(C_24_H_36_N_4_)(C_2_H_3_O_2_)]PF_6_ · 1.0 H_2_O	47.21	6.25	8.47	47.45	6.07	8.53
(Ni/2) [Ni(C_24_H_36_N_4_)(C_2_H_3_O_2_)]PF_6_ · 1.0 H_2_O	47.22	6.25	8.45	47.54	6.25	8.29
(Cu/2) [Cu(C_24_H_36_N_4_)](PF_6_)_2_ · 1.0 H_2_O	38.33	5.09	7.45	38.69	4.74	7.38
(Zn/2) [Zn(C_24_H_36_N_4_)(C_2_H_3_O_2_)]PF_6_ · 0.1 H_2_O	47.91	6.06	8.60	47.62	5.82	8.44

## Data Availability

Data presented in this study are available in the supplementary material or upon request from the corresponding authors.

## Supplementary Materials

The following supporting information can be downloaded at: https://www.mdpi.com/article/10.3390/molecules28020895/s1, Tables S1–S63 and Figures S1–S8: X-ray crystallography data. Figure S9–S14: Cyclic Voltammograms for Co, Ni and Cu complexes of Ligands **1** and **2**. Figures S15–S20: UV-Vis Spectra of Co, Ni, and Cu complexes of Ligands **1** and **2**. Figures S21 and S22: Example Kinetic Study for Dissociation of [Cu(**1**)]PF_6_ in H_2_O 5M HCl 30 °C at 640 nm.

## Sample Availability

Samples of the compounds are available from the authors.
