# Supplementary material for: A Bridge too Far? Comparison of Transition Metal Complexes of Dibenzyltetraazamacrocycles with and without Ethylene Cross-Bridges: X-ray Crystal Structures, Kinetic Stability, and Electronic Properties
Source: Molecules. 2023 Jan 16;28(2):895. doi: 10.3390/molecules28020895 (PMC9863842; doi:10.3390/molecules28020895)
Supplement: Supplementary file 1 [file molecules-28-00895-s001.zip › molecules-2114179-supplementary.pdf]

A bridge too far? Comparison of transition metal complexes of dibenzyltetraazamacrocycles with and without ethylene cross-bridges: X-ray crystal structures, kinetic stability, and electronic properties.

Ashlie N. Walker[1], Megan A. Ayala[1], Somrita Mondal[1], Mackenzie C. Bergagnini[1], Phuong John D. Bui[1], Stephanie N. Chidester[1], Chad I. Doeden[1], Louise Esjornson[1], Brian R. Sweany[1], Leslie Garcia[1], Jeanette A. Krause[2], Allen G. Oliver[3], Timothy J. Prior[4],* and Timothy J. Hubin[1,*]

Supplementary Information

Table of Contents **Page**

1. Crystallographic Details for all new structures 2
2. Tables S1-S8. Figure S1. X-Ray Crystallography for **[**Ni(**1**)(OH_2)$_2$]Cl_2. 3-8
3. Tables S9-S16. Figure S2. X-Ray Crystallography for [Ni(**1**)(μ-$OOCCH_3$)]PF_6 9-18
4. Tables S17-S24. Figure S3. X-Ray Crystallography for [Ni(**1**)($OOCCH_3$)(OH_2)]($OOCCH_3$)·H_2O 19-25
5. Tables S25-S32. Figure S4. X-Ray Crystallography for [Cu(**1**)(NH_3)](PF_6)$_2$ 26-31
6. Tables S33-S40. Figure S5. X-Ray Crystallography for [Zn(**1**)($OOCCH_3$)]PF_6 32-40
7. Tables S41-S48. Figure S6. X-Ray Crystallography for [Co(**2**)(μ-$OOCCH_3$)]PF_6 41-48
8. Tables S49-S55. Figure S7. X-Ray Crystallography for [Cu(**2**)](PF_6)$_2$ 49-53
9. Tables S56-S63. Figure S8. X-Ray Crystallography for [Ni(**2’**)(OAc)](PF_6).MeCN 54-60
10. Cyclic Voltammograms for Co, Ni and Cu complexes of Ligands **1** and **2**. Figures S9-S14. 61-63
11. UV-Vis Spectra of Co, Ni, and Cu complexes of Ligands **1** and **2**. Figures S15-S20. 64-66
12. Example Kinetic Study for Dissociation of [Cu(**1**)]PF_6 in H_20 5M HCl 30°C at 640nm
Figures S21-S22 67

Crystallographic details

Structures were refined using Olex2 and disorder was treated conservatively using standard methods. The scattering factors for atoms at this wavelength (0.7749 Å) were calculated using the XDISP program, which using the Cromer Liberman method with a Kissel-Pratt correction (see Kissel and Pratt, *Acta Crsyt*. A46, 170 (1990))

[Ni(**1**)(OH_2)$_2$]Cl_2. The molecule resides on a mirror plane but it is also disordered with two different orientations of the macrocycle. Some atoms positions are common to the two orientations. Disorder treated using standard methods.

[Ni(**1**)(μ-$OOCCH_3$)]PF6. The asymmetric unit contains a whole complex, but there is disorder here; there are two different nickel atom positions and hence two different positions for the cyclen part of the ligand, but the benzyl groups have positions common to both groups. This was modelled using bond length restraints. There is disorder in the PF_6^- anion and this appears to rock along one axis.

[Ni(**1**)($OOCCH_3$)(OH_2)]($OOCCH_3$)·H_2O. A routine refinement. There is small-scale disorder in the position the carboxylate group of the unbound acetate.

[Cu(**1**)(NH_3)](PF_6)$_2$**.** The ammonia molecule bound here was identified by comparison of the wR(F^2) values for structural models with bound water or bound ammonia and from examination of the Fourier difference map around this ligand. (Ammonia wR(F^2) = 6.41 %; water 7.26 %) Crucially the Fourier map has three regions of electron density consistent with the three hydrogen atoms of ammonia.
There is beautiful disorder; the cyclen portion of the ligand occupies different orientations but the benzyl groups do not display disorder. This was readily modelled using standard techniques without the need for restraints.

[Zn(**1**)($OOCCH_3$)]PF_6. There is a single complex in the asymmetric unit. In line with the other structures, disorder was limited to the cyclen portion of the ligand. Disorder was treated using standard methods and restraints to ensure reasonable bond lengths.

[Co(**2**)(μ-$OOCCH_3$)]PF_6. The refinement proceeded smoothly. Minor disorder in one of the two benzyl groups was handled using standard methods.

[Cu(**2**)](PF_6)$_2$. Routine structure. No unusual features in the refinement.

[Ni(**2'**)(OAc)](PF_6).MeCN. Routine structure. No unusual features in the refinement.

Table S1 Crystal data and structure refinement for [Ni(**1**)(OH$_2$)$_2$]Cl$_2$. **[TJH421A]**

Identification code	TJH421A
Empirical formula	$C_{22}H_{36}Cl_2N_4NiO_3$
Formula weight	534.16
Temperature/K	150
Crystal system	tetragonal
Space group	P4$_2$/ncm
a/Å	23.0551(15)
b/Å	23.0551(15)
c/Å	9.8183(8)
α/°	90
β/°	90
γ/°	90
Volume/Å^3	5218.8(8)
Z	8
ρ_{calc}g/cm^3	1.360
μ/mm^{-1}	1.232
F(000)	2256.0
Crystal size/mm^3	0.09 × 0.05 × 0.04
Radiation	synchrotron (λ = 0.7749 Å)
2Θ range for data collection/°	5.448 to 54.918
Index ranges	-27 ≤ h ≤ 27, -27 ≤ k ≤ 27, -11 ≤ l ≤ 11
Reflections collected	73497
Independent reflections	2402 [R_{int} = 0.0758, R_{sigma} = 0.0222]
Data/restraints/parameters	2402/188/194
Goodness-of-fit on F^2	1.044
Final R indexes [I>=2σ (I)]	R_1 = 0.0703, wR_2 = 0.1805
Final R indexes [all data]	R_1 = 0.0893, wR_2 = 0.1945
Largest diff. peak/hole / e Å^{-3}	0.77/-0.65

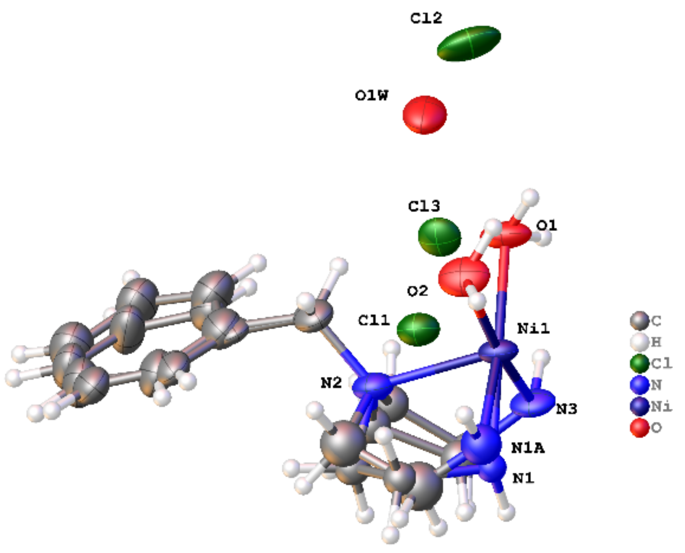

Figure S1: Asymmetric unit of [Ni(**1**)(OH$_2$)$_2$]Cl$_2$ **showing two disorder components with atoms as 50 % probability ellipsoids.**

Table S2 Fractional Atomic Coordinates (×10⁴) and Equivalent Isotropic Displacement Parameters (Å²×10³) for TJH421A. U_{eq} is defined as 1/3 of of the trace of the orthogonalised U_{IJ} tensor.

Atom	*x*	*y*	*z*	U(eq)
Ni1	6041.8(2)	6041.8(2)	7065.4(8)	46.2(3)
O1	5751.4(16)	5751.4(16)	5153(5)	72.5(16)
O2	6583.0(17)	6583.0(17)	5889(6)	80.4(18)
N2	5368(2)	6667.0(19)	7471(4)	59.6(11)
N3	5461(2)	5461(2)	7868(6)	63.6(17)
C5	5229(3)	7025(3)	6266(6)	81.0(18)
N1	6313(4)	6313(4)	8971(12)	41(3)
C1	6158(5)	6957(6)	9068(13)	51(3)
C2	5520(5)	6963(6)	8780(11)	60(3)
C3	4852(5)	6403(6)	8034(15)	66(4)
C4	5041(7)	5835(6)	8677(15)	66(5)
C6	4673(11)	7441(11)	6350(30)	98(8)
C7	4642(9)	7971(11)	7020(30)	111(8)
C8	4121(9)	8273(9)	7060(30)	118(10)
C9	3631(9)	8046(11)	6430(30)	131(10)
C10	3662(11)	7516(12)	5760(30)	125(8)
C11	4183(14)	7213(11)	5720(30)	112(7)
N1A	6475(6)	6475(6)	8608(18)	76(5)
C1A	6108(8)	6783(10)	9200(20)	109(8)
C2A	5658(7)	7100(7)	8406(17)	97(6)
C3A	4855(5)	6246(5)	7711(15)	63(4)
C4A	4974(6)	5715(6)	8529(14)	57(5)
C6A	4716(10)	7395(9)	6410(30)	79(5)
C7A	4821(8)	7938(9)	6970(20)	79(5)
C8A	4367(8)	8328(7)	7140(20)	83(5)
C9A	3808(8)	8175(8)	6750(20)	97(6)
C10A	3703(9)	7632(10)	6190(30)	114(7)
C11A	4157(12)	7242(8)	6020(30)	95(6)
Cl1	7500	7500	6804(3)	67.3(8)
Cl2	5715(3)	5920(3)	2019(3)	122(4)
Cl3	4461.9(14)	5538.1(14)	5000	66.9(11)
O1W	6486(4)	6751(4)	3223(8)	93(3)

Table S3 Anisotropic Displacement Parameters (Å²×10³) for TJH421A. The Anisotropic displacement factor exponent takes the form: $-2\pi^2[h^2a^{*2}U_{11}+2hka^*b^*U_{12}+\ldots]$.

Atom	U_{11}	U_{22}	U_{33}	U_{23}	U_{13}	U_{12}
Ni1	55.8(4)	55.8(4)	27.0(4)	1.7(2)	1.7(2)	-21.0(4)
O1	92(3)	92(3)	33(2)	-0.2(17)	-0.2(17)	-31(3)
O2	85(3)	85(3)	72(4)	10.9(19)	10.9(19)	-43(3)
N2	75(3)	68(3)	36(2)	5.4(19)	4(2)	-10(2)
N3	76(3)	76(3)	39(3)	14.1(19)	14.1(19)	-23(3)
C5	121(5)	70(4)	52(3)	22(3)	18(3)	9(3)
C6	128(11)	87(12)	78(18)	15(10)	8(11)	25(9)

Table S3 Anisotropic Displacement Parameters (Å²×10³) for TJH421A. The Anisotropic displacement factor exponent takes the form: $-2\pi^2[h^2a^{*2}U_{11}+2hka^*b^*U_{12}+\ldots]$.

Atom	U_{11}	U_{22}	U_{33}	U_{23}	U_{13}	U_{12}
C7	152(17)	78(11)	103(15)	17(9)	-5(13)	45(11)
C8	150(20)	89(14)	111(16)	1(11)	-23(15)	50(12)
C9	161(17)	114(17)	119(18)	-4(13)	-29(15)	39(14)
C10	151(12)	116(16)	110(18)	2(12)	-33(13)	42(12)
C11	137(11)	105(15)	94(16)	20(10)	-17(11)	30(10)
C6A	134(9)	67(9)	37(10)	24(7)	14(9)	17(7)
C7A	113(11)	67(9)	58(9)	21(7)	15(8)	22(7)
C8A	113(13)	71(9)	66(10)	16(7)	2(9)	21(8)
C9A	124(12)	71(9)	96(15)	0(9)	-30(12)	35(9)
C10A	155(12)	81(11)	105(17)	-16(11)	-53(13)	44(9)
C11A	144(11)	69(11)	71(12)	-2(9)	-12(10)	32(9)
Cl1	76.0(11)	76.0(11)	49.8(14)	0	0	-9.7(15)
Cl2	132(5)	182(8)	51.3(15)	23(3)	-32(3)	-24(6)
Cl3	66.7(15)	66.7(15)	67(2)	-16.6(13)	-16.6(13)	-20.0(19)
O1W	102(7)	106(7)	70(5)	24(4)	40(4)	19(6)

Table S4 Bond Lengths for TJH421A.

Atom	Atom	Length/Å	Atom	Atom	Length/Å
Ni1	O1	2.103(5)	C1	C2	1.499(11)
Ni1	O2	2.109(5)	C3	C4	1.516(12)
Ni1	N2	2.157(5)	C6	C7	1.3900
Ni1	N2[1]	2.157(5)	C6	C11	1.3900
Ni1	N3	2.050(6)	C7	C8	1.3900
Ni1	N1	2.070(11)	C8	C9	1.3900
Ni1	N1A	2.072(16)	C9	C10	1.3900
N2	C5	1.479(7)	C10	C11	1.3900
N2	C2	1.497(10)	N1A	C1A[1]	1.25(2)
N2	C3	1.445(11)	N1A	C1A	1.25(2)
N2	C2A	1.513(12)	C1A	C2A	1.489(14)
N2	C3A	1.547(11)	C3A	C4A	1.489(12)
N3	C4	1.521(11)	C6A	C7A	1.3900
N3	C4[1]	1.521(11)	C6A	C11A	1.3900
N3	C4A	1.423(10)	C7A	C8A	1.3900
N3	C4A[1]	1.423(10)	C8A	C9A	1.3900
C5	C6	1.60(2)	C9A	C10A	1.3900
C5	C6A	1.464(18)	C10A	C11A	1.3900
N1	C1	1.529(14)	Cl2	Cl2[1]	0.669(19)
N1	C1[1]	1.529(14)			

[1]+Y,+X,+Z

Table S5 Bond Angles for TJH421A.

Atom	Atom	Atom	Angle/°	Atom	Atom	Atom	Angle/°
O1	Ni1	O2	83.6(2)	C4A	N3	C4^1	118.1(6)
O1	Ni1	N2^1	98.53(11)	C4A^1	N3	C4^1	13.0(11)
O1	Ni1	N2	98.53(11)	C4A	N3	C4A^1	116.2(10)
O2	Ni1	N2	97.60(11)	N2	C5	C6	117.9(13)
O2	Ni1	N2^1	97.59(11)	C6A	C5	N2	115.2(11)
N2^1	Ni1	N2	158.3(2)	C1^1	N1	Ni1	106.2(7)
N3	Ni1	O1	85.8(2)	C1	N1	Ni1	106.2(7)
N3	Ni1	O2	169.4(2)	C1	N1	C1^1	116.7(11)
N3	Ni1	N2^1	83.97(12)	C2	C1	N1	103.2(10)
N3	Ni1	N2	83.96(12)	N2	C2	C1	112.8(9)
N3	Ni1	N1	92.7(4)	N2	C3	C4	106.7(10)
N3	Ni1	N1A	110.4(6)	C3	C4	N3	117.0(10)
N1	Ni1	O1	178.6(4)	C7	C6	C5	126.2(17)
N1	Ni1	O2	97.9(4)	C7	C6	C11	120.0
N1	Ni1	N2	81.30(12)	C11	C6	C5	113.7(17)
N1	Ni1	N2^1	81.30(12)	C8	C7	C6	120.0
N1A	Ni1	O1	163.7(6)	C7	C8	C9	120.0
N1A	Ni1	O2	80.2(6)	C10	C9	C8	120.0
N1A	Ni1	N2^1	83.69(14)	C9	C10	C11	120.0
N1A	Ni1	N2	83.69(14)	C10	C11	C6	120.0
C5	N2	Ni1	112.4(4)	C1A	N1A	Ni1	106.7(13)
C5	N2	C2	118.9(7)	C1A^1	N1A	Ni1	106.7(13)
C5	N2	C2A	102.3(8)	C1A^1	N1A	C1A	124(2)
C5	N2	C3A	107.9(6)	N1A	C1A	C2A	120.4(18)
C2	N2	Ni1	107.1(5)	C1A	C2A	N2	107.6(13)
C3	N2	Ni1	112.4(6)	C4A	C3A	N2	117.2(10)
C3	N2	C5	111.3(7)	N3	C4A	C3A	103.7(9)
C3	N2	C2	93.3(9)	C7A	C6A	C5	114.9(15)
C2A	N2	Ni1	103.6(7)	C7A	C6A	C11A	120.0
C2A	N2	C3A	131.3(10)	C11A	C6A	C5	125.1(15)
C3A	N2	Ni1	99.1(6)	C6A	C7A	C8A	120.0
C4	N3	Ni1	104.3(7)	C9A	C8A	C7A	120.0
C4^1	N3	Ni1	104.3(7)	C8A	C9A	C10A	120.0
C4	N3	C4^1	116.7(11)	C9A	C10A	C11A	120.0
C4A^1	N3	Ni1	114.9(7)	C10A	C11A	C6A	120.0
C4A	N3	Ni1	114.9(7)				

^{1}+Y,+X,+Z

Table S6 Torsion Angles for TJH421A.

A	B	C	D	Angle/°	A	B	C	D	Angle/°
Ni1	N2	C5	C6	171.0(13)	C1^1	N1	C1	C2	173.8(8)
Ni1	N2	C5	C6A	171.0(11)	C2	N2	C5	C6	-62.7(15)
Ni1	N2	C2	C1	25.7(12)	C2	N2	C3	C4	-87.2(12)
Ni1	N2	C3	C4	22.9(13)	C3	N2	C5	C6	43.8(16)

Table S6 Torsion Angles for TJH421A.

A	B	C	D	Angle/°	A	B	C	D	Angle/°
Ni1	N2	C2A	C1A	-29.9(16)	C3	N2	C2	C1	140.3(12)
Ni1	N2	C3A	C4A	41.8(12)	C4[1]	N3	C4	C3	155.7(7)
Ni1	N3	C4	C3	41.4(15)	C6	C7	C8	C9	0.0
Ni1	N3	C4A	C3A	34.8(13)	C7	C6	C11	C10	0.0
Ni1	N1	C1	C2	55.6(11)	C7	C8	C9	C10	0.0
Ni1	N1A	C1A	C2A	-40(3)	C8	C9	C10	C11	0.0
N2	C5	C6	C7	78(2)	C9	C10	C11	C6	0.0
N2	C5	C6	C11	-99.5(15)	C11	C6	C7	C8	0.0
N2	C5	C6A	C7A	87.7(14)	N1A	C1A	C2A	N2	52(3)
N2	C5	C6A	C11A	-92.7(14)	C1A[1]	N1A	C1A	C2A	-164.5(14)
N2	C3	C4	N3	-43.9(18)	C2A	N2	C5	C6A	-78.6(14)
N2	C3A	C4A	N3	-52.9(16)	C2A	N2	C3A	C4A	-75.4(16)
C5	N2	C2	C1	-103.1(11)	C3A	N2	C5	C6A	62.7(14)
C5	N2	C3	C4	150.0(10)	C3A	N2	C2A	C1A	85.6(17)
C5	N2	C2A	C1A	-146.9(14)	C4A[1]	N3	C4A	C3A	173.1(7)
C5	N2	C3A	C4A	159.0(11)	C6A	C7A	C8A	C9A	0.0
C5	C6	C7	C8	-177(3)	C7A	C6A	C11A	C10A	0.0
C5	C6	C11	C10	177(2)	C7A	C8A	C9A	C10A	0.0
C5	C6A	C7A	C8A	179.6(18)	C8A	C9A	C10A	C11A	0.0
C5	C6A	C11A	C10A	-180(2)	C9A	C10A	C11A	C6A	0.0
N1	C1	C2	N2	-53.8(14)	C11A	C6A	C7A	C8A	0.0

[1]+Y,+X,+Z

Table S7 Hydrogen Atom Coordinates (Å×10^4) and Isotropic Displacement Parameters (Å^2×10^3) for TJH421A.

Atom	*x*	*y*	*z*	U(eq)
H1A	5617.78	5378.91	5200.85	109
H1B	6045.11	5709.18	4595.85	109
H2A	6731.1	6406.71	5129.15	121
H2B	6916.85	6688.87	6329.76	121
H3	5252.06	5252.06	7122.56	76
H5AA	5174.33	6760.86	5481.19	97
H5AB	5570.75	7269.87	6062.89	97
H5BC	5567.03	7274.5	6059.56	97
H5BD	5169.82	6764.36	5476.53	97
H1	6094.38	6094.37	9684.09	49
H1C	6242.26	7111	9988.36	61
H1D	6374.44	7186.96	8385.24	61
H2C	5313.17	6769.53	9537.48	72
H2D	5384.8	7370.59	8740.79	72
H3A	4563.37	6330.51	7307.5	79
H3B	4675.59	6659.66	8727.14	79
H4A	4688.98	5602.2	8861.84	79
H4B	5222.14	5924.45	9566.85	79

Table S7 Hydrogen Atom Coordinates (Å×10⁴) and Isotropic Displacement Parameters (Å²×10³) for TJH421A.

Atom	*x*	*y*	*z*	U(eq)
H7	4977.2	8125.9	7448.46	133
H8	4099.78	8635.1	7521.72	141
H9	3274.62	8252.36	6464.34	157
H10	3326.89	7360.42	5333.68	151
H11	4204.31	6851.21	5260.4	134
H1AA	6749.18	6749.18	8149.19	91
H1AB	6322.92	7075.32	9741.07	130
H1AC	5901.87	6531.39	9859.22	130
H2AA	5839.49	7414.2	7866.42	117
H2AB	5368.9	7274.99	9028.36	117
H3AA	4540.49	6464.08	8166.75	75
H3AB	4706.64	6122.11	6810.54	75
H4AA	5067.11	5815.85	9484.76	68
H4AB	4637.42	5448.94	8517.72	68
H7A	5203.14	8043.16	7234.67	95
H8A	4438.95	8699.43	7526.61	100
H9A	3497.92	8441.4	6870.77	117
H10A	3321.06	7527.1	5922.96	137
H11A	4085.25	6870.82	5631	114

Table S8 Atomic Occupancy for TJH421A.

Atom	*Occupancy*	Atom	*Occupancy*	Atom	*Occupancy*
H1A	0.5	H1B	0.5	H2A	0.5
H2B	0.5	H5AA	0.5	H5AB	0.5
H5BC	0.5	H5BD	0.5	N1	0.5
H1	0.5	C1	0.5	H1C	0.5
H1D	0.5	C2	0.5	H2C	0.5
H2D	0.5	C3	0.5	H3A	0.5
H3B	0.5	C4	0.5	H4A	0.5
H4B	0.5	C6	0.5	C7	0.5
H7	0.5	C8	0.5	H8	0.5
C9	0.5	H9	0.5	C10	0.5
H10	0.5	C11	0.5	H11	0.5
N1A	0.5	H1AA	0.5	C1A	0.5
H1AB	0.5	H1AC	0.5	C2A	0.5
H2AA	0.5	H2AB	0.5	C3A	0.5
H3AA	0.5	H3AB	0.5	C4A	0.5
H4AA	0.5	H4AB	0.5	C6A	0.5
C7A	0.5	H7A	0.5	C8A	0.5
H8A	0.5	C9A	0.5	H9A	0.5
C10A	0.5	H10A	0.5	C11A	0.5
H11A	0.5	Cl2	0.5	Cl3	0.5
O1W	0.5				

Table S9 Crystal data and structure refinement for [Ni(**1**)(μ-OOCCH$_3$)]PF$_6$ **[MAA07]**

Identification code	MAA07
Empirical formula	$C_{24}H_{35}F_6N_4NiO_2P$
Formula weight	615.24
Temperature/K	150
Crystal system	monoclinic
Space group	C2/c
a/Å	20.0955(16)
b/Å	12.0653(16)
c/Å	22.230(2)
α/°	90
β/°	91.953(3)
γ/°	90
Volume/Å^3	5386.8(10)
Z	8
ρ_{calc}g/cm^3	1.517
μ/mm^{-1}	1.070
F(000)	2560.0
Crystal size/mm^3	0.05 × 0.03 × 0.025
Radiation	synchrotron (λ = 0.7749 Å)
2Θ range for data collection/°	4.294 to 58.074
Index ranges	-25 ≤ h ≤ 25, -15 ≤ k ≤ 15, -27 ≤ l ≤ 27
Reflections collected	28526
Independent reflections	5533 [R_{int} = 0.0543, R_{sigma} = 0.0400]
Data/restraints/parameters	5533/2107/462
Goodness-of-fit on F^2	1.150
Final R indexes [I>=2σ (I)]	R_1 = 0.0927, wR_2 = 0.2045
Final R indexes [all data]	R_1 = 0.1128, wR_2 = 0.2141
Largest diff. peak/hole / e Å^{-3}	1.28/-0.77

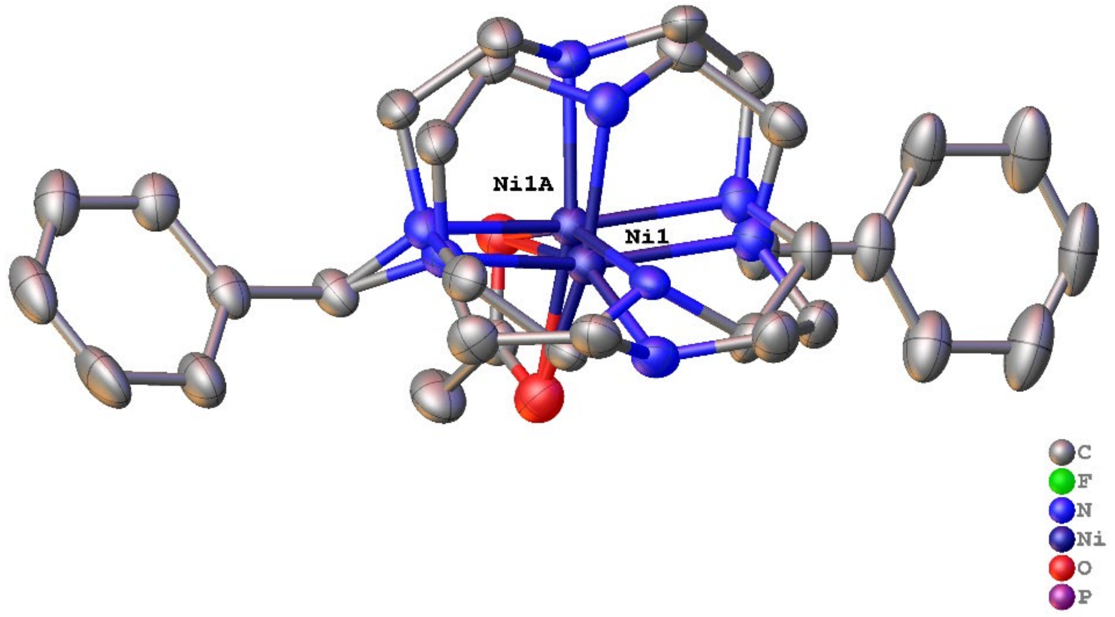

Figure S2: Asymmetric unit of [Ni(**1**)(μ-OOCCH$_3$)]PF$_6$ **showing two disorder components with atoms as 50 % probability ellipsoids. (Hydrogen atoms and anion omitted for clarity)**

Table S10 Fractional Atomic Coordinates (×10⁴) and Equivalent Isotropic Displacement Parameters (Å²×10³) for MAA07. U_{eq} is defined as 1/3 of of the trace of the orthogonalised U_{IJ} tensor.

Atom	*x*	*y*	*z*	U(eq)
P1	3493.2(9)	3922.2(19)	2742.6(7)	56.6(6)
F1	3649(2)	4039(4)	3441.7(16)	66.2(13)
F6	3330(3)	3855(4)	2032.6(17)	79.3(15)
F2	2713(3)	3917(6)	2903(3)	55(2)
F3	3397(5)	5306(7)	2721(4)	69(3)
F4	4231(5)	4070(10)	2599(4)	82(3)
F5	3537(5)	2657(7)	2774(4)	79(3)
F2A	3047(6)	2832(10)	2795(5)	88(4)
F3A	2981(7)	4830(12)	2695(7)	107(5)
F4A	4119(4)	4687(8)	2603(4)	44(2)
F5A	4079(6)	2939(10)	2713(5)	87(4)
O1	4411.3(19)	3529(3)	4852.3(18)	39.1(9)
O2	3640.6(18)	2351(3)	5092.9(16)	31.1(8)
C9	2832(3)	4454(5)	4681(3)	37.3(13)
C10	2319(3)	5181(5)	4356(3)	42.9(14)
C11	1653(3)	5135(5)	4475(3)	53.5(17)
C12	1186(4)	5758(6)	4135(4)	68(2)
C13	1396(5)	6424(6)	3674(4)	68(2)
C14	2054(5)	6459(6)	3541(4)	71(2)
C15	2516(4)	5851(6)	3887(3)	55.1(17)
C16	4958(3)	2081(5)	5975(2)	34.0(12)
C17	5473(3)	1479(5)	6376(2)	35.6(12)
C18	5293(3)	541(5)	6701(3)	41.6(13)
C19	5764(4)	-11(6)	7058(3)	49.6(15)
C20	6407(4)	383(7)	7112(3)	55.2(17)
C21	6580(3)	1277(7)	6793(3)	60.0(18)
C22	6122(3)	1846(6)	6421(3)	47.8(15)
C23	4099(3)	2648(5)	4744(2)	32.3(11)
C24	4261(3)	1934(6)	4223(3)	47.9(16)
Ni1	3916.8(15)	3849(3)	5590.9(14)	23.9(5)
N1	3117(5)	4890(9)	5242(5)	29(2)
N2	4421(4)	5259(7)	5745(4)	33.0(18)
N3	4624(6)	3109(10)	6220(5)	27(2)
N4	3315(4)	3755(8)	6321(3)	31.3(17)
C1	3454(5)	5978(8)	5173(5)	36(2)
C2	3955(7)	6186(11)	5684(7)	41(3)
C3	4795(6)	5139(10)	6314(5)	37(3)
C4	5123(5)	4019(9)	6314(5)	37(2)
C5	4257(5)	2862(10)	6773(4)	34(2)
C6	3529(5)	2712(9)	6619(5)	35(2)
C7	2629(5)	3848(9)	6064(5)	33(2)
C8	2619(5)	4915(9)	5711(4)	36(2)
Ni1A	3777(2)	3569(4)	5696(2)	24.4(8)
N1A	2984(8)	4636(12)	5368(7)	30(3)

Table S10 Fractional Atomic Coordinates (×10^4) and Equivalent Isotropic Displacement Parameters (Å^2×10^3) for MAA07. U_{eq} is defined as 1/3 of of the trace of the orthogonalised U_{IJ} tensor.

Atom	*x*	*y*	*z*	U(eq)
N2A	4150(5)	5021(9)	6041(5)	26(2)
N3A	4533(10)	2835(13)	6302(8)	30(3)
N4A	3127(6)	3111(11)	6330(5)	30(2)
C1A	3207(7)	5779(11)	5495(7)	37(3)
C2A	3962(9)	5847(15)	5579(9)	38(4)
C3A	4867(7)	4801(14)	6153(7)	32(3)
C4A	4908(8)	3785(12)	6556(7)	34(3)
C5A	4133(7)	2266(14)	6753(7)	33(3)
C6A	3513(7)	2894(15)	6886(7)	34(3)
C7A	2627(7)	3982(13)	6346(7)	35(3)
C8A	2405(7)	4312(15)	5730(6)	39(3)

Table S11 Anisotropic Displacement Parameters (Å^2×10^3) for MAA07. The Anisotropic displacement factor exponent takes the form: $-2\pi^2[h^2a^{*2}U_{11}+2hka^*b^*U_{12}+\ldots]$.

Atom	U_{11}	U_{22}	U_{33}	U_{23}	U_{13}	U_{12}
P1	55.0(11)	86.5(15)	27.3(8)	9.2(9)	-10.4(7)	-34.1(10)
F1	53(2)	114(4)	31.3(19)	0(2)	-6.7(17)	-6(2)
F6	103(4)	99(4)	34(2)	12(2)	-18(2)	-47(3)
O1	35(2)	40(2)	41(2)	4.1(17)	-3.1(17)	-1.7(17)
O2	30.8(19)	35(2)	27.2(18)	-0.7(15)	0.7(14)	-0.1(16)
C9	47(3)	31(3)	34(3)	-3(2)	-9(2)	6(2)
C10	55(3)	30(3)	42(3)	-8(2)	-20(3)	7(3)
C11	53(3)	33(3)	72(5)	6(3)	-30(3)	-1(3)
C12	62(4)	42(4)	96(6)	-2(4)	-45(4)	2(3)
C13	93(5)	30(3)	77(5)	-6(3)	-47(4)	17(4)
C14	108(5)	44(4)	58(4)	5(3)	-28(4)	17(4)
C15	76(4)	45(4)	43(4)	1(3)	-12(3)	16(3)
C16	31(3)	46(3)	24(3)	-1(2)	-1(2)	9(2)
C17	37(3)	44(3)	26(3)	-7(2)	-1(2)	14(2)
C18	48(3)	42(3)	35(3)	-5(2)	-4(2)	8(3)
C19	65(4)	45(4)	38(3)	-1(3)	-6(3)	19(3)
C20	54(3)	76(5)	35(3)	-1(3)	-5(3)	36(3)
C21	34(3)	92(5)	54(4)	2(4)	0(3)	29(3)
C22	37(3)	59(4)	47(4)	6(3)	2(3)	17(3)
C23	29(3)	41(3)	27(2)	2(2)	-5.0(19)	4(2)
C24	52(4)	60(4)	32(3)	-4(3)	10(3)	13(3)
Ni1	22.7(11)	27.1(13)	21.8(10)	-1.1(8)	-0.5(7)	0.9(8)
N1	32(4)	29(4)	27(4)	2(3)	-2(3)	2(3)
N2	29(4)	34(4)	37(4)	0(3)	4(3)	-5(3)
N3	20(4)	37(5)	26(4)	2(4)	-2(3)	1(3)
N4	35(4)	30(4)	30(4)	-1(3)	2(3)	1(3)
C1	35(5)	28(4)	43(5)	6(4)	0(4)	1(3)
C2	38(5)	34(6)	50(6)	3(5)	-2(4)	-2(4)

Table S11 Anisotropic Displacement Parameters (Å^2×10^3) for MAA07. The Anisotropic displacement factor exponent takes the form: $-2\pi^2[h^2a^{*2}U_{11}+2hka^*b^*U_{12}+…]$.

Atom	U_{11}	U_{22}	U_{33}	U_{23}	U_{13}	U_{12}
C3	27(5)	43(5)	42(6)	1(5)	2(4)	-4(4)
C4	31(5)	42(5)	38(6)	-2(4)	-6(4)	-6(4)
C5	39(4)	35(5)	27(4)	4(4)	5(3)	8(4)
C6	40(4)	38(5)	27(5)	7(5)	9(4)	2(4)
C7	30(4)	40(5)	29(5)	-6(4)	6(4)	0(4)
C8	27(4)	44(5)	38(5)	1(4)	0(4)	3(4)
Ni1A	24.9(16)	27.3(18)	20.9(15)	-1.5(11)	-0.3(10)	-0.4(11)
N1A	35(6)	30(6)	25(5)	-3(4)	-1(4)	6(4)
N2A	23(4)	34(5)	23(5)	5(4)	6(4)	3(4)
N3A	33(6)	34(6)	24(6)	2(5)	4(4)	5(4)
N4A	31(5)	32(6)	27(5)	5(4)	6(4)	1(4)
C1A	41(6)	30(6)	40(7)	-3(5)	-8(5)	8(5)
C2A	42(6)	32(8)	41(8)	12(6)	-1(6)	3(6)
C3A	24(5)	41(7)	30(7)	8(6)	6(5)	6(5)
C4A	31(6)	43(6)	27(6)	7(5)	-4(5)	4(5)
C5A	34(6)	37(7)	30(6)	12(6)	7(4)	6(5)
C6A	35(6)	40(7)	28(6)	5(6)	8(5)	5(5)
C7A	28(6)	34(7)	42(7)	8(6)	9(6)	-2(5)
C8A	37(6)	44(7)	38(6)	3(6)	4(5)	8(5)

Table S12 Bond Lengths for MAA07.

Atom	Atom	Length/Å	Atom	Atom	Length/Å
P1	F1	1.581(4)	C23	Ni1	2.413(6)
P1	F6	1.603(4)	C23	Ni1A	2.496(7)
P1	F2	1.619(7)	Ni1	N1	2.162(12)
P1	F3	1.681(8)	Ni1	N2	2.003(8)
P1	F4	1.537(9)	Ni1	N3	2.154(13)
P1	F5	1.531(9)	Ni1	N4	2.060(8)
P1	F2A	1.598(11)	N1	C1	1.487(11)
P1	F3A	1.504(12)	N1	C8	1.469(11)
P1	F4A	1.599(8)	N2	C2	1.463(13)
P1	F5A	1.674(11)	N2	C3	1.455(12)
O1	C23	1.253(7)	N3	C4	1.496(12)
O1	Ni1	1.986(5)	N3	C5	1.486(12)
O1	Ni1A	2.305(6)	N4	C6	1.479(12)
O2	C23	1.276(6)	N4	C7	1.478(11)
O2	Ni1	2.181(5)	C1	C2	1.513(13)
O2	Ni1A	2.002(6)	C3	C4	1.504(13)
C9	C10	1.518(8)	C5	C6	1.503(13)
C9	N1	1.454(12)	C7	C8	1.508(13)
C9	N1A	1.563(17)	Ni1A	N1A	2.156(18)
C10	C11	1.373(10)	Ni1A	N2A	2.044(12)
C10	C15	1.388(10)	Ni1A	N3A	2.18(2)
C11	C12	1.403(9)	Ni1A	N4A	2.030(12)

Table S12 Bond Lengths for MAA07.

Atom	Atom	Length/Å	Atom	Atom	Length/Å
C12	C13	1.379(12)	N1A	C1A	1.474(15)
C13	C14	1.367(13)	N1A	C8A	1.490(15)
C14	C15	1.393(10)	N2A	C2A	1.471(16)
C16	C17	1.526(7)	N2A	C3A	1.479(15)
C16	N3	1.520(14)	N3A	C4A	1.474(16)
C16	N3A	1.46(2)	N3A	C5A	1.475(15)
C17	C18	1.397(9)	N4A	C6A	1.461(14)
C17	C22	1.379(9)	N4A	C7A	1.454(14)
C18	C19	1.385(8)	C1A	C2A	1.525(17)
C19	C20	1.377(10)	C3A	C4A	1.520(16)
C20	C21	1.343(11)	C5A	C6A	1.496(15)
C21	C22	1.397(9)	C7A	C8A	1.480(16)
C23	C24	1.487(8)			

Table S13 Bond Angles for MAA07.

Atom	Atom	Atom	Angle/°	Atom	Atom	Atom	Angle/°
F1	P1	F6	177.7(3)	N3	Ni1	N1	160.0(5)
F1	P1	F2	86.8(3)	N4	Ni1	O2	102.0(3)
F1	P1	F3	87.6(4)	N4	Ni1	C23	133.7(3)
F1	P1	F2A	95.5(5)	N4	Ni1	N1	82.3(3)
F1	P1	F4A	90.5(3)	N4	Ni1	N3	81.8(4)
F1	P1	F5A	89.2(5)	C9	N1	Ni1	111.4(7)
F6	P1	F2	92.9(3)	C9	N1	C1	113.4(8)
F6	P1	F3	90.1(4)	C9	N1	C8	111.0(9)
F6	P1	F5A	92.6(5)	C1	N1	Ni1	102.4(7)
F2	P1	F3	84.2(4)	C8	N1	Ni1	105.9(7)
F4	P1	F1	91.9(4)	C8	N1	C1	112.2(9)
F4	P1	F6	88.1(4)	C2	N2	Ni1	108.4(7)
F4	P1	F2	173.5(5)	C3	N2	Ni1	107.9(7)
F4	P1	F3	89.4(5)	C3	N2	C2	117.8(10)
F5	P1	F1	92.0(4)	C16	N3	Ni1	113.2(7)
F5	P1	F6	90.2(4)	C4	N3	C16	110.3(9)
F5	P1	F2	92.4(5)	C4	N3	Ni1	102.2(7)
F5	P1	F3	176.5(5)	C5	N3	C16	111.8(8)
F5	P1	F4	94.0(6)	C5	N3	Ni1	106.6(8)
F2A	P1	F6	86.2(5)	C5	N3	C4	112.4(10)
F2A	P1	F4A	159.4(6)	C6	N4	Ni1	103.3(6)
F2A	P1	F5A	79.5(6)	C7	N4	Ni1	104.9(6)
F3A	P1	F1	96.7(6)	C7	N4	C6	119.3(9)
F3A	P1	F6	81.4(6)	N1	C1	C2	111.3(9)
F3A	P1	F2A	102.6(8)	N2	C2	C1	110.5(10)
F3A	P1	F4A	96.2(7)	N2	C3	C4	107.6(9)
F3A	P1	F5A	173.5(7)	N3	C4	C3	111.6(9)
F4A	P1	F6	88.5(3)	N3	C5	C6	110.0(9)
F4A	P1	F5A	81.0(5)	N4	C6	C5	105.4(9)

Table S13 Bond Angles for MAA07.

Atom	Atom	Atom	Angle/°	Atom	Atom	Atom	Angle/°
C23	O1	Ni1	93.6(4)	N4	C7	C8	105.1(8)
C23	O1	Ni1A	83.5(3)	N1	C8	C7	110.7(8)
C23	O2	Ni1	84.2(3)	O1	Ni1A	C23	29.92(17)
C23	O2	Ni1A	96.7(4)	O2	Ni1A	O1	60.39(19)
C10	C9	N1A	119.4(8)	O2	Ni1A	C23	30.52(18)
N1	C9	C10	116.2(6)	O2	Ni1A	N1A	97.4(5)
C11	C10	C9	122.3(6)	O2	Ni1A	N2A	156.9(4)
C11	C10	C15	118.3(6)	O2	Ni1A	N3A	101.0(5)
C15	C10	C9	119.2(6)	O2	Ni1A	N4A	100.9(4)
C10	C11	C12	120.8(7)	N1A	Ni1A	O1	99.2(4)
C13	C12	C11	119.8(8)	N1A	Ni1A	C23	100.9(5)
C14	C13	C12	120.1(7)	N1A	Ni1A	N3A	160.3(7)
C13	C14	C15	119.8(8)	N2A	Ni1A	O1	96.7(3)
C10	C15	C14	121.3(8)	N2A	Ni1A	C23	126.6(4)
N3	C16	C17	118.5(6)	N2A	Ni1A	N1A	82.7(5)
N3A	C16	C17	113.6(9)	N2A	Ni1A	N3A	83.0(5)
C18	C17	C16	120.4(5)	N3A	Ni1A	O1	95.8(5)
C22	C17	C16	120.7(6)	N3A	Ni1A	C23	98.4(5)
C22	C17	C18	118.9(5)	N4A	Ni1A	O1	161.1(4)
C19	C18	C17	120.2(6)	N4A	Ni1A	C23	131.3(4)
C20	C19	C18	120.2(7)	N4A	Ni1A	N1A	85.0(5)
C21	C20	C19	119.4(6)	N4A	Ni1A	N2A	102.1(5)
C20	C21	C22	122.0(7)	N4A	Ni1A	N3A	85.0(5)
C17	C22	C21	119.2(7)	C9	N1A	Ni1A	111.4(9)
O1	C23	O2	119.2(5)	C1A	N1A	C9	111.5(11)
O1	C23	C24	121.3(5)	C1A	N1A	Ni1A	106.1(10)
O1	C23	Ni1	55.2(3)	C1A	N1A	C8A	112.4(14)
O1	C23	Ni1A	66.6(3)	C8A	N1A	C9	111.1(12)
O2	C23	C24	119.5(5)	C8A	N1A	Ni1A	104.0(10)
O2	C23	Ni1	64.0(3)	C2A	N2A	Ni1A	103.7(9)
O2	C23	Ni1A	52.8(3)	C2A	N2A	C3A	117.7(12)
C24	C23	Ni1	176.0(4)	C3A	N2A	Ni1A	104.6(9)
C24	C23	Ni1A	171.0(5)	C16	N3A	Ni1A	110.6(10)
O1	Ni1	O2	62.95(17)	C16	N3A	C4A	112.1(14)
O1	Ni1	C23	31.22(18)	C16	N3A	C5A	113.0(12)
O1	Ni1	N1	101.7(3)	C4A	N3A	Ni1A	104.9(10)
O1	Ni1	N2	92.4(3)	C4A	N3A	C5A	112.7(14)
O1	Ni1	N3	96.8(4)	C5A	N3A	Ni1A	102.9(12)
O1	Ni1	N4	164.7(3)	C6A	N4A	Ni1A	107.5(9)
O2	Ni1	C23	31.74(17)	C7A	N4A	Ni1A	106.3(9)
N1	Ni1	O2	97.2(3)	C7A	N4A	C6A	117.2(12)
N1	Ni1	C23	101.5(3)	N1A	C1A	C2A	111.6(13)
N2	Ni1	O2	155.3(3)	N2A	C2A	C1A	106.3(14)
N2	Ni1	C23	123.6(3)	N2A	C3A	C4A	106.0(12)
N2	Ni1	N1	86.2(4)	N3A	C4A	C3A	112.7(12)
N2	Ni1	N3	85.5(4)	N3A	C5A	C6A	112.1(14)

Table S13 Bond Angles for MAA07.

Atom	Atom	Atom	Angle/°	Atom	Atom	Atom	Angle/°
N2	Ni1	N4	102.6(4)	N4A	C6A	C5A	110.2(12)
N3	Ni1	O2	97.9(3)	N4A	C7A	C8A	111.1(13)
N3	Ni1	C23	98.2(4)	C7A	C8A	N1A	110.7(13)

Table S14 Torsion Angles for MAA07.

A	B	C	D	Angle/°	A	B	C	D	Angle/°
C9	C10	C11	C12	-175.2(6)	Ni1	N4	C6	C5	59.2(9)
C9	C10	C15	C14	174.4(6)	Ni1	N4	C7	C8	-55.6(9)
C9	N1	C1	C2	-158.1(10)	N1	C9	C10	C11	-87.8(8)
C9	N1	C8	C7	90.6(10)	N1	C9	C10	C15	97.7(8)
C9	N1A	C1A	C2A	-100.2(16)	N1	C1	C2	N2	53.5(15)
C9	N1A	C8A	C7A	153.6(13)	N2	C3	C4	N3	-57.0(14)
C10	C9	N1	Ni1	-173.3(5)	N3	C16	C17	C18	98.2(7)
C10	C9	N1	C1	-58.4(10)	N3	C16	C17	C22	-82.2(8)
C10	C9	N1	C8	69.0(9)	N3	C5	C6	N4	-57.5(12)
C10	C9	N1A	Ni1A	-176.1(5)	N4	C7	C8	N1	58.7(11)
C10	C9	N1A	C1A	-57.7(14)	C1	N1	C8	C7	-141.3(9)
C10	C9	N1A	C8A	68.4(13)	C2	N2	C3	C4	168.4(10)
C10	C11	C12	C13	0.1(11)	C3	N2	C2	C1	-160.9(11)
C11	C10	C15	C14	-0.4(10)	C4	N3	C5	C6	136.7(11)
C11	C12	C13	C14	1.3(11)	C5	N3	C4	C3	-77.3(13)
C12	C13	C14	C15	-2.2(12)	C6	N4	C7	C8	-170.6(8)
C13	C14	C15	C10	1.8(11)	C7	N4	C6	C5	175.0(8)
C15	C10	C11	C12	-0.6(10)	C8	N1	C1	C2	75.1(13)
C16	C17	C18	C19	179.1(5)	Ni1A	O1	C23	O2	4.0(5)
C16	C17	C22	C21	179.9(6)	Ni1A	O1	C23	C24	-175.0(5)
C16	N3	C4	C3	157.2(10)	Ni1A	O2	C23	O1	-4.6(5)
C16	N3	C5	C6	-98.6(11)	Ni1A	O2	C23	C24	174.4(5)
C16	N3A	C4A	C3A	95.6(17)	Ni1A	N1A	C1A	C2A	21.2(16)
C16	N3A	C5A	C6A	-155.0(14)	Ni1A	N1A	C8A	C7A	33.6(16)
C17	C16	N3	Ni1	179.8(5)	Ni1A	N2A	C2A	C1A	56.9(15)
C17	C16	N3	C4	66.1(10)	Ni1A	N2A	C3A	C4A	-55.4(13)
C17	C16	N3	C5	-59.7(10)	Ni1A	N3A	C4A	C3A	-24.4(16)
C17	C16	N3A	Ni1A	-176.4(5)	Ni1A	N3A	C5A	C6A	-35.7(16)
C17	C16	N3A	C4A	67.0(13)	Ni1A	N4A	C6A	C5A	-42.2(16)
C17	C16	N3A	C5A	-61.6(16)	Ni1A	N4A	C7A	C8A	44.8(15)
C17	C18	C19	C20	2.4(9)	N1A	C9	C10	C11	-67.1(10)
C18	C17	C22	C21	-0.5(9)	N1A	C9	C10	C15	118.3(8)
C18	C19	C20	C21	-3.1(10)	N1A	C1A	C2A	N2A	-53.2(19)
C19	C20	C21	C22	2.1(11)	N2A	C3A	C4A	N3A	54.6(19)
C20	C21	C22	C17	-0.3(11)	N3A	C16	C17	C18	81.0(9)
C22	C17	C18	C19	-0.5(9)	N3A	C16	C17	C22	-99.4(9)
Ni1	O1	C23	O2	1.3(5)	N3A	C5A	C6A	N4A	55(2)
Ni1	O1	C23	C24	-177.7(5)	N4A	C7A	C8A	N1A	-54.8(19)
Ni1	O2	C23	O1	-1.2(5)	C1A	N1A	C8A	C7A	-80.8(18)

Table S14 Torsion Angles for MAA07.

A	B	C	D	Angle/°	A	B	C	D	Angle/°
Ni1	O2	C23	C24	177.8(5)	C2A	N2A	C3A	C4A	-169.8(14)
Ni1	N1	C1	C2	-38.0(11)	C3A	N2A	C2A	C1A	171.8(13)
Ni1	N1	C8	C7	-30.4(10)	C4A	N3A	C5A	C6A	77(2)
Ni1	N2	C2	C1	-38.1(14)	C5A	N3A	C4A	C3A	-135.6(15)
Ni1	N2	C3	C4	45.4(11)	C6A	N4A	C7A	C8A	165.0(14)
Ni1	N3	C4	C3	36.6(11)	C7A	N4A	C6A	C5A	-161.7(13)
Ni1	N3	C5	C6	25.6(11)	C8A	N1A	C1A	C2A	134.3(16)

Table S15 Hydrogen Atom Coordinates (Å×10^4) and Isotropic Displacement Parameters (Å^2×10^3) for MAA07.

Atom	*x*	*y*	*z*	U(eq)
H9AA	2621.66	3731.1	4765.91	45
H9AB	3198.97	4313.07	4405.17	45
H9BC	3256.28	4537.85	4472.33	45
H9BD	2688.08	3674.55	4626.27	45
H11	1507.43	4676.16	4791.97	64
H12	726.93	5721	4222.28	81
H13	1081.61	6857.99	3448.53	81
H14	2196.75	6897.31	3214.88	85
H15	2974.79	5896.11	3799.72	66
H16A	5177.91	2295.85	5599.85	41
H16B	4604.15	1543.29	5860.87	41
H16C	5193.43	2508.37	5666.2	41
H16D	4674.8	1524.64	5762.48	41
H18	4846.96	281.81	6677.48	50
H19	5643.77	-664.39	7266.58	60
H20	6724.47	26.48	7372.16	66
H21	7027.83	1529.93	6821.31	72
H22	6256.83	2478.92	6201.27	57
H24A	4729.52	2037.26	4127.31	72
H24B	3976.95	2139.46	3874.15	72
H24C	4183.82	1156.04	4325.98	72
H2	4750.9	5336.23	5420.96	40
H4	3410.59	4394.63	6596.74	38
H1A	3116.51	6575.88	5163.58	43
H1B	3683.43	5991.13	4785.84	43
H2A	4203.33	6876.68	5605.29	49
H2B	3718.62	6282.77	6063.87	49
H3A	4491.88	5198.32	6654.3	44
H3B	5135.05	5729.95	6355.57	44
H4A	5449.01	3991.57	5990.48	45
H4B	5368	3904.94	6703.11	45
H5A	4317.56	3479.05	7064	40
H5B	4437.61	2179.07	6964.04	40
H6A	3458.29	2073.18	6344.47	42

Table S15 Hydrogen Atom Coordinates (Å×10[4]) and Isotropic Displacement Parameters (Å[2]×10[3]) for MAA07.

Atom	*x*	*y*	*z*	U(eq)
H6B	3276.12	2584.43	6987.15	42
H7A	2303.04	3874.17	6387.72	39
H7B	2521.61	3210.23	5798.04	39
H8A	2171.13	5028.02	5522.17	43
H8B	2714.2	5544.48	5986.54	43
H2AA	3933.41	5201.42	6427.34	32
H4AA	2904.04	2407.7	6197.17	36
H1AA	2997.06	6049.14	5863.55	45
H1AB	3060.07	6266.68	5157.14	45
H2AB	4179.17	5678.62	5196.38	46
H2AC	4097.99	6599.1	5712.18	46
H3AA	5085.75	5443.63	6354.36	38
H3AB	5088.9	4658.21	5769.62	38
H4AB	5380.69	3570.93	6619.71	41
H4AC	4730.71	3974.52	6953.58	41
H5AA	4404.82	2173.38	7129.18	40
H5AB	4010.33	1519.22	6601.81	40
H6AA	3241.63	2458.37	7164.61	41
H6AB	3632.77	3604.34	7084.85	41
H7AA	2815.47	4633.83	6561.26	41
H7AB	2240.06	3715.38	6568.41	41
H8AA	2091.62	4943.39	5751.98	47
H8AB	2166.28	3686.52	5530.99	47

Table S16 Atomic Occupancy for MAA07.

Atom	*Occupancy*	Atom	*Occupancy*	Atom	*Occupancy*
F2	0.548(8)	F3	0.548(8)	F4	0.548(8)
F5	0.548(8)	F2A	0.452(8)	F3A	0.452(8)
F4A	0.452(8)	F5A	0.452(8)	H9AA	0.588(7)
H9AB	0.588(7)	H9BC	0.412(7)	H9BD	0.412(7)
H16A	0.588(7)	H16B	0.588(7)	H16C	0.412(7)
H16D	0.412(7)	Ni1	0.588(7)	N1	0.588(7)
N2	0.588(7)	H2	0.588(7)	N3	0.588(7)
N4	0.588(7)	H4	0.588(7)	C1	0.588(7)
H1A	0.588(7)	H1B	0.588(7)	C2	0.588(7)
H2A	0.588(7)	H2B	0.588(7)	C3	0.588(7)
H3A	0.588(7)	H3B	0.588(7)	C4	0.588(7)
H4A	0.588(7)	H4B	0.588(7)	C5	0.588(7)
H5A	0.588(7)	H5B	0.588(7)	C6	0.588(7)
H6A	0.588(7)	H6B	0.588(7)	C7	0.588(7)
H7A	0.588(7)	H7B	0.588(7)	C8	0.588(7)
H8A	0.588(7)	H8B	0.588(7)	Ni1A	0.412(7)
N1A	0.412(7)	N2A	0.412(7)	H2AA	0.412(7)
N3A	0.412(7)	N4A	0.412(7)	H4AA	0.412(7)

Table S16 Atomic Occupancy for MAA07.

Atom	*Occupancy*	Atom	*Occupancy*	Atom	*Occupancy*
C1A	0.412(7)	H1AA	0.412(7)	H1AB	0.412(7)
C2A	0.412(7)	H2AB	0.412(7)	H2AC	0.412(7)
C3A	0.412(7)	H3AA	0.412(7)	H3AB	0.412(7)
C4A	0.412(7)	H4AB	0.412(7)	H4AC	0.412(7)
C5A	0.412(7)	H5AA	0.412(7)	H5AB	0.412(7)
C6A	0.412(7)	H6AA	0.412(7)	H6AB	0.412(7)
C7A	0.412(7)	H7AA	0.412(7)	H7AB	0.412(7)
C8A	0.412(7)	H8AA	0.412(7)	H8AB	0.412(7)

Table S17 Crystal data and structure refinement for [Ni(**1**)(OOCCH_3)(OH_2)](OOCCH_3)·H_2O. **[TJH422-P21n]**

Identification code	TJH422-P21n
Empirical formula	$C_{26}H_{42}N_4NiO_6$
Formula weight	565.34
Temperature/K	150
Crystal system	monoclinic
Space group	$P2_1/n$
a/Å	17.0242(13)
b/Å	7.8117(6)
c/Å	22.7132(17)
α/°	90
β/°	108.604(2)
γ/°	90
Volume/Å^3	2862.7(4)
Z	4
ρ_{calc}g/cm^3	1.312
μ/mm^{-1}	0.905
F(000)	1208.0
Crystal size/mm^3	0.07 × 0.06 × 0.03
Radiation	synchrotron (λ = 0.7749 Å)
2Θ range for data collection/°	4.126 to 59.718
Index ranges	-21 ≤ h ≤ 21, -10 ≤ k ≤ 10, -29 ≤ l ≤ 29
Reflections collected	41189
Independent reflections	6328 [R_{int} = 0.0502, R_{sigma} = 0.0346]
Data/restraints/parameters	6328/6/339
Goodness-of-fit on F^2	1.048
Final R indexes [I>=2σ (I)]	R_1 = 0.0411, wR_2 = 0.0906
Final R indexes [all data]	R_1 = 0.0576, wR_2 = 0.0971
Largest diff. peak/hole / e Å^{-3}	0.47/-0.41

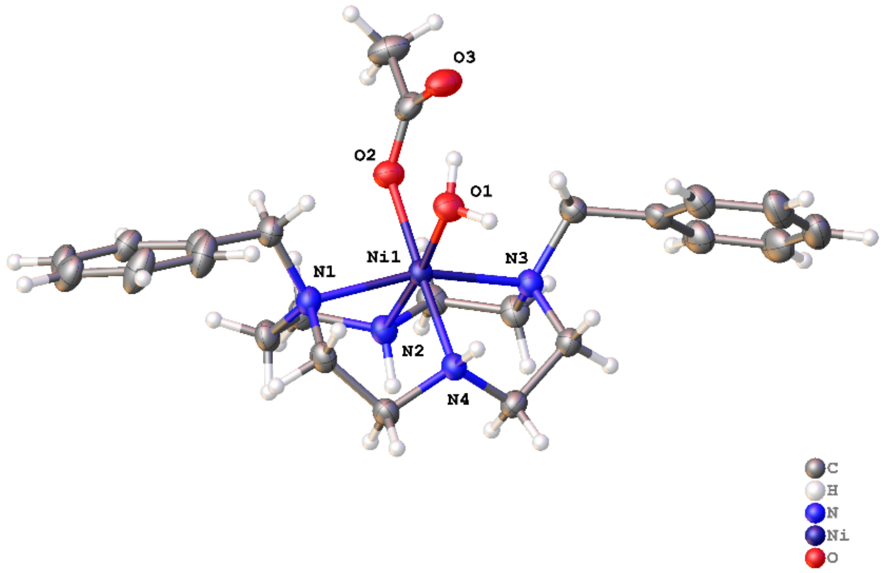

Figure S3: Metal complex within [Ni(**1**)(OOCCH_3)(OH_2)](OOCCH_3)·H_2O **with atoms as 50 % probability ellipsoids.**

Table S18 Fractional Atomic Coordinates (×10[4]) and Equivalent Isotropic Displacement Parameters (Å[2]×10[3]) for TJH422-P21n. U_{eq} is defined as 1/3 of of the trace of the orthogonalised U_{IJ} tensor.

Atom	*x*	*y*	*z*	U(eq)
Ni1	2662.8(2)	8069.2(3)	5278.7(2)	21.81(8)
O1	2673.9(9)	5414.0(18)	5268.1(7)	30.6(3)
O2	1436.7(9)	7946.9(19)	5221.6(7)	29.8(3)
O3	1274.0(10)	5261(2)	5491.2(8)	40.3(4)
N1	2439.3(11)	8352(2)	4299.7(8)	26.3(4)
N2	2520.9(11)	10720(2)	5240.6(8)	26.9(4)
N3	3126.2(10)	8587(2)	6272.6(8)	24.9(4)
N4	3888.8(10)	8022(2)	5337.5(7)	24.3(4)
C1	2223.2(14)	10185(3)	4146.5(10)	30.1(5)
C2	1912.2(13)	11036(3)	4626.5(10)	31.1(5)
C3	2285.1(14)	11139(3)	5789.0(10)	30.6(5)
C4	2938.0(14)	10424(3)	6358.5(10)	30.3(5)
C5	4031.6(12)	8306(3)	6422.2(9)	27.1(5)
C6	4345.5(12)	8953(3)	5905.7(9)	26.7(4)
C7	3959.3(13)	8680(3)	4749.1(9)	27.9(5)
C8	3250.4(13)	7916(3)	4224.3(9)	28.9(5)
C9	1772.4(13)	7146(3)	3946.4(10)	30.9(5)
C10	1682.6(13)	6903(3)	3269.1(10)	30.0(5)
C11	2134.5(15)	5641(3)	3086.1(11)	41.3(6)
C12	2072.2(15)	5437(4)	2466.0(11)	45.9(7)
C13	1554.7(14)	6489(3)	2021.7(11)	38.5(6)
C14	1090.1(14)	7707(3)	2192.9(10)	34.2(5)
C15	1144.9(13)	7905(3)	2809.8(10)	31.2(5)
C16	2760.3(13)	7386(3)	6629.8(9)	28.0(5)
C17	3287.9(13)	7024(3)	7290.4(10)	30.0(5)
C18	3407.9(15)	8207(3)	7768.1(10)	38.2(5)
C19	3927.4(17)	7838(4)	8364.8(11)	46.0(7)
C20	4313.3(16)	6280(4)	8491.0(12)	48.4(7)
C21	4187.0(16)	5072(4)	8031.4(12)	46.5(7)
C22	3677.8(15)	5445(3)	7432.1(11)	38.7(6)
C23	1030.0(13)	6767(3)	5369.4(10)	29.7(5)
C24	187.5(14)	7243(3)	5405.2(13)	43.2(6)
O4A	4938(3)	5096(6)	5863(2)	38.8(12)
O5	3895(3)	3506(6)	6045(4)	39.9(18)
C25	4646.7(14)	3858(3)	6074.9(12)	35.4(5)
C26	5243.6(16)	3027(3)	6664.5(12)	46.4(6)
O7	6156.4(13)	6740(2)	5513.6(10)	53.6(5)
O5A	3957.0(19)	3270(4)	5810(3)	37.9(12)
O4	4899(4)	4790(10)	5747(4)	46(2)

Table S19 Anisotropic Displacement Parameters (Å^2×10^3) for TJH422-P21n. The Anisotropic displacement factor exponent takes the form: -2π^2[h^{2}a*^{2}U$_{11}$+2hka*b*U$_{12}$+…].

Atom	U11	U22	U33	U23	U13	U12
Ni1	22.85(13)	20.59(14)	21.07(13)	1.47(11)	5.71(10)	1.57(11)
O1	31.6(8)	23.8(8)	36.1(8)	3.9(7)	10.4(7)	1.4(6)
O2	26.4(7)	27.7(8)	35.2(8)	2.2(7)	9.8(6)	0.0(6)
O3	29.7(8)	31.0(9)	55.6(11)	6.7(8)	6.9(8)	-5.5(7)
N1	27.3(9)	26.8(9)	22.5(8)	1.1(7)	4.9(7)	0.8(7)
N2	29.1(9)	24.1(9)	26.7(9)	3.2(7)	8.0(8)	3.1(7)
N3	27.3(9)	23.3(9)	23.6(9)	0.4(7)	7.5(7)	0.7(7)
N4	26.7(8)	24.1(9)	21.8(8)	0.4(7)	7.4(7)	-0.8(7)
C1	32.4(11)	29.2(12)	26.0(11)	7.2(9)	5.6(9)	4.2(9)
C2	31.8(11)	26.3(11)	31.9(12)	6.1(9)	5.4(9)	5.7(9)
C3	37.6(12)	23.7(11)	32.8(12)	-0.2(9)	14.4(10)	4.0(9)
C4	37.6(12)	25.4(11)	27.8(11)	-2.4(9)	10.2(10)	2.3(9)
C5	25.7(10)	30.3(12)	23.2(10)	-0.1(9)	5.0(8)	-0.7(9)
C6	24.8(10)	27.7(11)	25.3(10)	-0.8(9)	4.7(8)	-2.1(9)
C7	26.9(10)	32.5(11)	25.1(10)	4.1(9)	9.4(9)	0.0(9)
C8	31.8(11)	32.8(12)	23.4(10)	2.2(9)	10.8(9)	2.9(9)
C9	30.7(11)	34.6(13)	24.5(10)	0.6(9)	4.6(9)	-1.2(10)
C10	26.9(10)	34.7(12)	24.5(10)	-2.3(10)	2.6(8)	0.0(10)
C11	39.8(13)	43.8(14)	29.4(12)	-4.7(11)	-4.4(10)	12.6(11)
C12	36.1(13)	59.8(17)	33.1(13)	-16.1(12)	-1.3(11)	15.9(12)
C13	32.7(12)	53.7(16)	25.6(11)	-9.0(11)	4.3(10)	-0.8(11)
C14	29.5(11)	39.1(13)	25.1(11)	0.7(10)	-4.0(9)	-2.8(10)
C15	24.2(10)	34.7(13)	29.9(11)	-3.0(10)	1.9(9)	2.8(9)
C16	29.2(11)	30.6(11)	25.1(10)	0.4(9)	9.9(9)	-3.9(9)
C17	29.8(11)	36.6(12)	26.1(10)	2.4(10)	12.6(9)	-5.3(10)
C18	45.3(13)	42.8(14)	29.5(12)	-1.3(11)	16.1(10)	-3.4(11)
C19	52.1(15)	61.4(18)	28.3(12)	-5.5(12)	18.3(11)	-13.4(14)
C20	38.9(14)	75(2)	30.9(13)	13.7(13)	10.0(11)	-3.4(14)
C21	45.1(14)	55.7(17)	40.8(14)	19.2(13)	16.4(12)	11.1(13)
C22	43.4(14)	39.7(14)	35.6(13)	5.8(11)	16.4(11)	2.3(11)
C23	25.8(10)	33.8(12)	25.5(10)	-5.2(9)	2.5(8)	-5.2(9)
C24	29.8(12)	43.5(15)	58.0(16)	-10.4(12)	16.4(11)	-7.8(11)
C25	38.2(13)	20.6(11)	51.2(15)	-3.8(10)	19.5(12)	0.4(10)
C26	47.6(14)	39.3(14)	45.7(15)	1.8(12)	5.7(12)	4.9(12)
O7	56.8(12)	46.0(11)	58.5(12)	-4.5(10)	19.2(10)	-7.0(9)

Table S20 Bond Lengths for TJH422-P21n.

Atom	Atom	Length/Å	Atom	Atom	Length/Å
Ni1	O1	2.0745(15)	C7	C8	1.522(3)
Ni1	O2	2.0518(14)	C9	C10	1.508(3)
Ni1	N1	2.1443(17)	C10	C11	1.393(3)
Ni1	N2	2.0832(18)	C10	C15	1.389(3)
Ni1	N3	2.1786(17)	C11	C12	1.388(3)

Table S20 Bond Lengths for TJH422-P21n.

Atom	Atom	Length/Å	Atom	Atom	Length/Å
Ni1	N4	2.0489(17)	C12	C13	1.379(3)
O2	C23	1.261(3)	C13	C14	1.370(3)
O3	C23	1.248(3)	C14	C15	1.383(3)
N1	C1	1.492(3)	C16	C17	1.509(3)
N1	C8	1.484(3)	C17	C18	1.390(3)
N1	C9	1.496(3)	C17	C22	1.389(3)
N2	C2	1.469(3)	C18	C19	1.392(3)
N2	C3	1.462(3)	C19	C20	1.370(4)
N3	C4	1.497(3)	C20	C21	1.372(4)
N3	C5	1.485(3)	C21	C22	1.392(3)
N3	C16	1.500(3)	C23	C24	1.509(3)
N4	C6	1.469(3)	O4A	C25	1.251(5)
N4	C7	1.472(3)	O5	C25	1.289(5)
C1	C2	1.510(3)	C25	C26	1.543(3)
C3	C4	1.518(3)	C25	O5A	1.225(4)
C5	C6	1.522(3)	C25	O4	1.214(6)

Table S21 Bond Angles for TJH422-P21n.

Atom	Atom	Atom	Angle/°	Atom	Atom	Atom	Angle/°
O1	Ni1	N1	95.17(6)	N2	C2	C1	107.93(17)
O1	Ni1	N2	174.08(6)	N2	C3	C4	108.55(17)
O1	Ni1	N3	101.32(6)	N3	C4	C3	111.76(17)
O2	Ni1	O1	88.03(6)	N3	C5	C6	111.60(16)
O2	Ni1	N1	95.67(6)	N4	C6	C5	107.17(16)
O2	Ni1	N2	86.65(6)	N4	C7	C8	107.48(17)
O2	Ni1	N3	95.68(6)	N1	C8	C7	110.76(17)
N1	Ni1	N3	160.26(7)	N1	C9	C10	116.02(18)
N2	Ni1	N1	82.81(7)	C11	C10	C9	120.5(2)
N2	Ni1	N3	81.79(7)	C15	C10	C9	121.5(2)
N4	Ni1	O1	88.29(7)	C15	C10	C11	118.0(2)
N4	Ni1	O2	176.31(6)	C12	C11	C10	120.9(2)
N4	Ni1	N1	84.81(6)	C13	C12	C11	119.9(2)
N4	Ni1	N2	97.04(7)	C14	C13	C12	119.9(2)
N4	Ni1	N3	84.90(6)	C13	C14	C15	120.4(2)
C23	O2	Ni1	130.62(14)	C14	C15	C10	120.9(2)
C1	N1	Ni1	106.83(12)	N3	C16	C17	115.91(17)
C1	N1	C9	113.01(16)	C18	C17	C16	122.9(2)
C8	N1	Ni1	103.43(12)	C22	C17	C16	119.1(2)
C8	N1	C1	111.24(17)	C22	C17	C18	118.0(2)
C8	N1	C9	111.63(16)	C17	C18	C19	120.8(2)
C9	N1	Ni1	110.16(12)	C20	C19	C18	120.1(3)
C2	N2	Ni1	104.12(13)	C19	C20	C21	120.3(2)
C3	N2	Ni1	104.30(13)	C20	C21	C22	119.8(3)
C3	N2	C2	117.96(17)	C17	C22	C21	121.0(2)
C4	N3	Ni1	106.94(12)	O2	C23	C24	116.5(2)

Table S21 Bond Angles for TJH422-P21n.

Atom	Atom	Atom	Angle/°	Atom	Atom	Atom	Angle/°
C4	N3	C16	112.72(17)	O3	C23	O2	125.1(2)
C5	N3	Ni1	102.47(12)	O3	C23	C24	118.4(2)
C5	N3	C4	111.23(16)	O4A	C25	C26	115.3(3)
C5	N3	C16	111.30(16)	O5	C25	C26	108.9(3)
C16	N3	Ni1	111.63(12)	O5A	C25	O4A	122.5(3)
C6	N4	Ni1	106.92(12)	O5A	C25	C26	122.0(3)
C6	N4	C7	116.83(16)	O4	C25	O5	129.4(4)
C7	N4	Ni1	108.12(12)	O4	C25	C26	121.4(4)
N1	C1	C2	111.82(17)				

Table S22 Torsion Angles for TJH422-P21n.

A	B	C	D	Angle/°	A	B	C	D	Angle/°
Ni1	O2	C23	O3	-13.1(3)	C5	N3	C4	C3	128.58(19)
Ni1	O2	C23	C24	166.30(15)	C5	N3	C16	C17	38.1(2)
Ni1	N1	C1	C2	-22.0(2)	C6	N4	C7	C8	-162.51(17)
Ni1	N1	C8	C7	-40.81(18)	C7	N4	C6	C5	167.60(17)
Ni1	N1	C9	C10	-166.44(15)	C8	N1	C1	C2	-134.18(18)
Ni1	N2	C2	C1	-53.74(18)	C8	N1	C9	C10	-52.1(2)
Ni1	N2	C3	C4	55.27(18)	C9	N1	C1	C2	99.3(2)
Ni1	N3	C4	C3	17.4(2)	C9	N1	C8	C7	-159.23(17)
Ni1	N3	C5	C6	37.91(18)	C9	C10	C11	C12	-178.3(2)
Ni1	N3	C16	C17	151.97(15)	C9	C10	C15	C14	177.8(2)
Ni1	N4	C6	C5	46.39(18)	C10	C11	C12	C13	-0.2(4)
Ni1	N4	C7	C8	-41.93(19)	C11	C10	C15	C14	-3.0(3)
N1	C1	C2	N2	52.0(2)	C11	C12	C13	C14	-1.5(4)
N1	C9	C10	C11	89.0(3)	C12	C13	C14	C15	0.9(4)
N1	C9	C10	C15	-91.8(3)	C13	C14	C15	C10	1.3(3)
N2	C3	C4	N3	-49.5(2)	C15	C10	C11	C12	2.4(4)
N3	C5	C6	N4	-59.1(2)	C16	N3	C4	C3	-105.6(2)
N3	C16	C17	C18	73.5(3)	C16	N3	C5	C6	157.35(17)
N3	C16	C17	C22	-106.1(2)	C16	C17	C18	C19	-177.4(2)
N4	C7	C8	N1	57.5(2)	C16	C17	C22	C21	178.3(2)
C1	N1	C8	C7	73.5(2)	C17	C18	C19	C20	-1.5(4)
C1	N1	C9	C10	74.2(2)	C18	C17	C22	C21	-1.3(3)
C2	N2	C3	C4	170.12(18)	C18	C19	C20	C21	-0.3(4)
C3	N2	C2	C1	-168.70(18)	C19	C20	C21	C22	1.3(4)
C4	N3	C5	C6	-76.1(2)	C20	C21	C22	C17	-0.4(4)
C4	N3	C16	C17	-87.6(2)	C22	C17	C18	C19	2.3(3)

Table S23 Hydrogen Atom Coordinates (Å×10[4]) and Isotropic Displacement Parameters (Å[2]×10[3]) for TJH422-P21n.

Atom	*x*	*y*	*z*	U(eq)
H1A	3122.19	4930.11	5505.62	46
H1B	2253.3	5001.01	5360.62	46
H2	3059.48	11281.78	5268.95	32
H4	4079.6	6804.52	5385.3	29
H1C	1790.72	10258.23	3734.99	36
H1D	2719.23	10801.88	4121.09	36
H2A	1847.39	12282.04	4546.07	37
H2B	1366.68	10556.78	4608.49	37
H3A	1737.56	10634.31	5750.36	37
H3B	2245.81	12396.43	5827.27	37
H4A	3452.05	11107.87	6443.97	36
H4B	2740.03	10526.42	6722.22	36
H5A	4152.57	7067.44	6486.56	32
H5B	4328.53	8906.75	6813.79	32
H6A	4246.68	10199.26	5845.73	32
H6B	4947.78	8737.51	6011.9	32
H7A	4498.3	8342.59	4705.04	34
H7B	3922.75	9945.05	4739.51	34
H8A	3270.39	8362.36	3821.37	35
H8B	3313	6656.61	4222.35	35
H9A	1237.02	7563.62	3975.48	37
H9B	1880.44	6013.88	4152.76	37
H11	2490.23	4910.63	3390.05	50
H12	2385.49	4574.4	2347.6	55
H13	1520.41	6370.26	1598.02	46
H14	727.89	8419.53	1885.78	41
H15	810.53	8737.53	2920.99	37
H16A	2639.63	6285.68	6402.49	34
H16B	2225.32	7867.3	6636.75	34
H18	3131.98	9280.03	7686.33	46
H19	4014.4	8668.44	8684.87	55
H20	4669.68	6034.04	8898.26	58
H21	4446.56	3983.15	8122.21	56
H22	3595.63	4606.83	7115.01	46
H24A	108.55	8483.42	5352.74	65
H24B	149.21	6904.81	5810.61	65
H24C	-242.37	6651.59	5075.51	65
H26A	5806.29	3458.9	6733.6	70
H26B	5237.5	1781.08	6611.58	70
H26C	5066.2	3311.4	7022.97	70
H7C	5778.36	6171.32	5595.57	80
H7D	6163.5	6480.84	5151.5	80

Table S24 Atomic Occupancy for TJH422-P21n.

Atom	*Occupancy*	Atom	*Occupancy*	Atom	*Occupancy*
O4A	0.587(13)	O5	0.413(13)	O5A	0.587(13)
O4	0.413(13)				

Table S25 Crystal data and structure refinement for [Cu(**1**)(NH_3)](PF_6)$_2$ **[SC07]**

Identification code	SC07
Empirical formula	$C_{22}H_{35}CuF_{12}N_5P_2$
Formula weight	723.03
Temperature/K	150
Crystal system	monoclinic
Space group	$P2_1/m$
a/Å	9.1979(8)
b/Å	14.6533(12)
c/Å	10.3138(9)
α/°	90
β/°	93.878(2)
γ/°	90
Volume/Å^3	1386.9(2)
Z	2
ρ_{calc}g/cm^3	1.731
μ/mm^{-1}	1.270
F(000)	738.0
Crystal size/mm^3	0.09 × 0.07 × 0.05
Radiation	synchrotron (λ = 0.7749 Å)
2Θ range for data collection/°	4.316 to 62.376
Index ranges	-12 ≤ h ≤ 12, -19 ≤ k ≤ 19, -13 ≤ l ≤ 13
Reflections collected	25074
Independent reflections	3584 [R_{int} = 0.0285, R_{sigma} = 0.0172]
Data/restraints/parameters	3584/5/268
Goodness-of-fit on F^2	1.036
Final R indexes [I>=2σ (I)]	R_1 = 0.0248, wR_2 = 0.0632
Final R indexes [all data]	R_1 = 0.0258, wR_2 = 0.0638
Largest diff. peak/hole / e Å^{-3}	0.37/-0.33

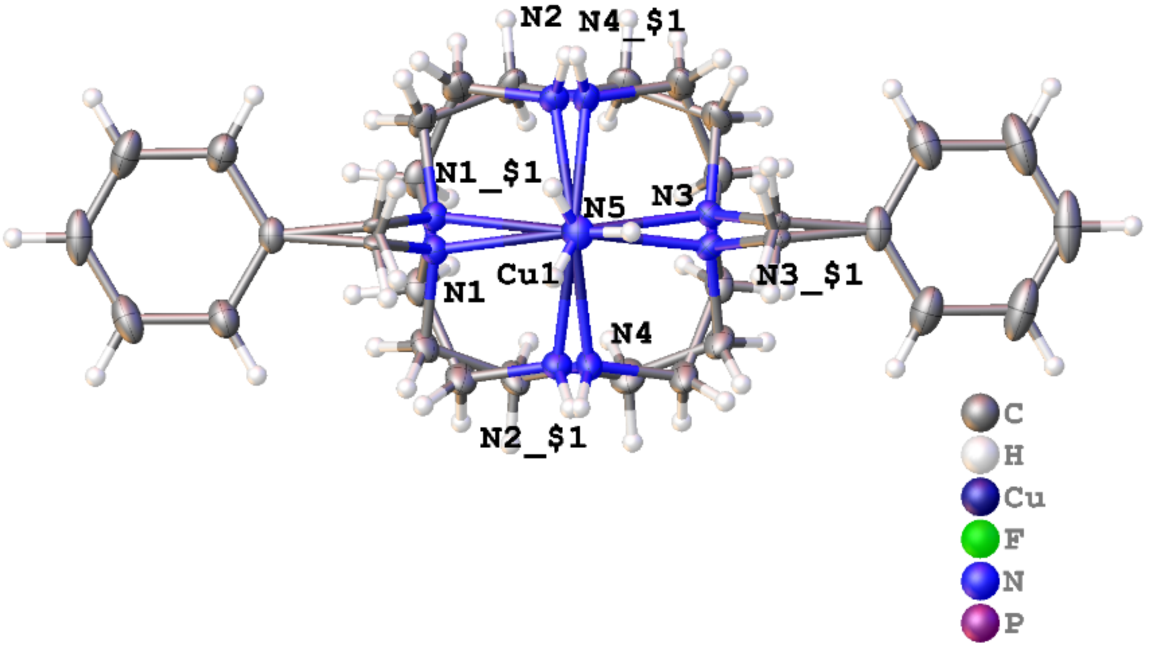

Figure S4: Metal complex within [Cu(**1**)(NH_3)](PF_6)$_2$ **with atoms as 50 % probability ellipsoids. The two disorder component generated by symmetry are both shown.**

Table S26 Fractional Atomic Coordinates (×10[4]) and Equivalent Isotropic Displacement Parameters (Å[2]×10[3]) for SC07. U_{eq} is defined as 1/3 of the trace of the orthogonalised U_{IJ} tensor.

Atom	*x*	*y*	*z*	U(eq)
Cu1	3475.5(2)	2500	7383.3(2)	14.72(6)
N5	4700.9(16)	2500	5715.7(15)	21.7(3)
N1	5013.2(19)	2334.1(16)	8938.7(18)	17.6(8)
N4	2926(4)	1183(4)	7711(4)	20.9(7)
N3	1335.5(19)	2663.3(16)	6655.8(18)	17.1(8)
C4	896(4)	3608(2)	7000(3)	26.1(6)
C3	1778(3)	3936(2)	8200(3)	30.0(6)
C8	4830(3)	1387(2)	9440(3)	23.0(5)
C1	4563(3)	3032(2)	9882(2)	24.9(5)
C7	3291(3)	1057.0(19)	9127(3)	26.3(5)
C2	4306(4)	3941(2)	9197(3)	26.8(6)
C6	1356(3)	1054(2)	7328(3)	26.6(6)
C5	570(3)	1962(2)	7392(2)	23.5(5)
C9	6525(2)	2412(10)	8514(2)	13.0(16)
C10	7756.7(18)	2500	9567.6(16)	20.7(3)
C11	8375.7(14)	3318.3(11)	10020.1(13)	28.6(3)
C12	9587.7(15)	3314.2(13)	10909.7(14)	36.2(3)
C13	10191(2)	2500	11349.2(19)	36.7(5)
C14	1144(2)	2584(13)	5211(2)	15.5(18)
C15	-413.0(19)	2500	4631.7(17)	26.1(4)
C16	-1120.4(16)	1683.7(13)	4322.8(15)	37.2(3)
C17	-2510.7(17)	1685.4(17)	3692.2(16)	49.3(5)
C18	-3195(2)	2500	3370(2)	52.4(8)
P1	6871.1(4)	5366.9(2)	6789.1(3)	26.20(9)
F1	6701.9(14)	4302.0(6)	6522.0(10)	50.9(3)
F2	8503.0(12)	5344.9(8)	6387.6(12)	55.9(3)
F3	7423.6(12)	5148.4(8)	8260.4(9)	50.1(3)
F4	7009.6(14)	6430.3(7)	7078.4(12)	54.4(3)
F5	5237.0(11)	5380.6(8)	7224.8(14)	60.6(3)
F6	6328.5(14)	5590.9(8)	5329.9(11)	59.5(3)
N2	3342(4)	3816(3)	8001(4)	20.9(7)

Table S27 Anisotropic Displacement Parameters (Å[2]×10[3]) for SC07. The Anisotropic displacement factor exponent takes the form: $-2\pi^2[h^2a^{*2}U_{11}+2hka^*b^*U_{12}+\ldots]$.

Atom	U_{11}	U_{22}	U_{33}	U_{23}	U_{13}	U_{12}
Cu1	14.46(10)	15.13(10)	14.65(10)	0	1.57(7)	0
N5	19.3(7)	27.7(8)	18.4(7)	0	4.1(5)	0
N1	16.7(7)	20(2)	16.3(7)	0.2(7)	1.1(6)	1.0(7)
N4	18.0(19)	21.3(12)	22.9(19)	5.6(13)	-3.1(12)	-1.9(14)
N3	16.7(7)	17(2)	17.5(8)	2.2(7)	1.9(6)	1.6(7)
C4	23.0(14)	26.7(17)	28.2(15)	-5.6(14)	-1.7(12)	10.9(12)
C3	26.9(13)	29.1(14)	34.0(16)	-10.1(12)	2.3(12)	9.3(11)

Table S27 Anisotropic Displacement Parameters (Å²×10³) for SC07. The Anisotropic displacement factor exponent takes the form: $-2\pi^2[h^2a^{*2}U_{11}+2hka^*b^*U_{12}+\ldots]$.

Atom	U_{11}	U_{22}	U_{33}	U_{23}	U_{13}	U_{12}
C8	21.7(14)	26.5(16)	20.5(13)	10.2(12)	-1.5(10)	0.2(12)
C1	23.7(11)	34.6(16)	16.5(11)	-5.2(11)	2.2(9)	2.8(11)
C7	21.7(14)	28.2(14)	28.6(14)	14.3(11)	-1.6(10)	-3.0(10)
C2	29(2)	25.1(16)	25.9(14)	-10.4(12)	-3.3(12)	4.3(12)
C6	21.5(14)	26.2(16)	31.4(19)	9.8(12)	-4.1(12)	-8.7(12)
C5	15.7(10)	33.0(16)	22.1(12)	6.6(11)	2.9(9)	-1.9(10)
C9	16.0(7)	7(5)	16.0(7)	2.8(12)	0.8(6)	-0.2(11)
C10	15.7(7)	30.1(9)	16.3(7)	0	2.0(6)	0
C11	23.0(6)	34.4(7)	28.2(6)	-5.8(6)	-1.0(5)	-1.1(5)
C12	23.9(6)	54.6(10)	29.7(7)	-14.1(7)	-1.0(5)	-7.0(6)
C13	18.9(8)	71.7(16)	19.0(9)	0	-1.2(7)	0
C14	17.5(7)	12(6)	16.5(8)	-1.1(13)	2.0(6)	2.1(13)
C15	17.2(8)	46.0(11)	15.2(8)	0	2.3(6)	0
C16	26.0(7)	54.3(10)	31.0(7)	-8.9(7)	0.5(5)	-6.6(6)
C17	26.2(7)	88.1(15)	33.5(8)	-16.2(9)	0.6(6)	-15.5(8)
C18	18.2(9)	120(3)	18.7(9)	0	0.6(7)	0
P1	27.06(17)	21.01(16)	29.68(18)	4.42(13)	-4.32(13)	-4.22(12)
F1	84.0(8)	21.0(4)	48.0(6)	3.1(4)	5.9(5)	-8.1(5)
F2	39.0(5)	61.5(7)	69.4(7)	19.5(6)	19.1(5)	2.1(5)
F3	53.3(6)	63.2(7)	32.0(5)	12.7(5)	-10.0(4)	-15.4(5)
F4	74.7(8)	24.7(5)	64.1(7)	-6.6(5)	6.8(6)	-13.7(5)
F5	28.0(5)	52.3(7)	102.0(10)	22.0(6)	8.2(5)	3.2(4)
F6	83.4(8)	45.7(6)	44.6(6)	20.3(5)	-30.5(6)	-25.6(6)
N2	22(2)	19.1(12)	22(2)	-3.2(13)	0.1(13)	1.6(15)

Table S28 Bond Lengths for SC07.

Atom	Atom	Length/Å	Atom	Atom	Length/Å
Cu1	N5	2.1191(15)	C6	C5	1.519(4)
Cu1	N1	2.0799(18)	C9	C10	1.521(3)
Cu1	N4	2.029(5)	C10	C11[1]	1.3942(16)
Cu1	N3	2.0724(18)	C10	C11	1.3942(16)
Cu1	N2	2.037(5)	C11	C12	1.3951(19)
N1	C8	1.495(4)	C12	C13	1.380(2)
N1	C1	1.489(3)	C14	C15	1.519(3)
N1	C9	1.490(3)	C15	C16	1.3884(19)
N4	C7	1.487(5)	C15	C16[1]	1.3883(19)
N4	C6	1.483(4)	C16	C17	1.395(2)
N3	C4	1.491(4)	C17	C18	1.379(3)
N3	C5	1.484(3)	P1	F1	1.5903(10)
N3	C14	1.493(3)	P1	F2	1.5842(11)
C4	C3	1.513(4)	P1	F3	1.5998(10)
C3	N2	1.477(5)	P1	F4	1.5900(10)
C8	C7	1.510(4)	P1	F5	1.5977(11)
C1	C2	1.519(4)	P1	F6	1.5875(10)

Table S28 Bond Lengths for SC07.

Atom	Atom	Length/Å	Atom	Atom	Length/Å
C2	N2	1.482(4)			

[1]+X,1/2-Y,+Z

Table S29 Bond Angles for SC07.

Atom	Atom	Atom	Angle/°	Atom	Atom	Atom	Angle/°
N1	Cu1	N5	104.76(7)	N1	C9	C10	117.48(16)
N4	Cu1	N5	106.70(15)	C11[1]	C10	C9	115.7(6)
N4	Cu1	N1	85.66(11)	C11	C10	C9	125.4(6)
N4	Cu1	N3	85.93(11)	C11	C10	C11[1]	118.65(17)
N4	Cu1	N2	145.81(8)	C10	C11	C12	120.43(15)
N3	Cu1	N5	104.50(7)	C13	C12	C11	120.38(15)
N3	Cu1	N1	150.74(7)	C12[1]	C13	C12	119.72(18)
N2	Cu1	N5	107.48(14)	N3	C14	C15	116.33(16)
N2	Cu1	N1	85.51(11)	C16[1]	C15	C14	115.8(7)
N2	Cu1	N3	85.87(11)	C16	C15	C14	125.1(7)
C8	N1	Cu1	106.77(17)	C16[1]	C15	C16	118.98(19)
C1	N1	Cu1	102.65(14)	C15	C16	C17	120.41(18)
C1	N1	C8	111.7(2)	C18	C17	C16	120.17(19)
C1	N1	C9	116.4(5)	C17[1]	C18	C17	119.8(2)
C9	N1	Cu1	111.34(16)	F1	P1	F3	89.37(6)
C9	N1	C8	107.6(6)	F1	P1	F5	88.75(7)
C7	N4	Cu1	103.8(3)	F2	P1	F1	91.04(7)
C6	N4	Cu1	109.0(3)	F2	P1	F3	89.89(6)
C6	N4	C7	113.4(4)	F2	P1	F4	90.21(7)
C4	N3	Cu1	106.70(17)	F2	P1	F5	178.74(7)
C4	N3	C14	107.1(7)	F2	P1	F6	89.95(7)
C5	N3	Cu1	101.94(14)	F4	P1	F1	178.64(7)
C5	N3	C4	112.0(2)	F4	P1	F3	90.11(7)
C5	N3	C14	115.4(6)	F4	P1	F5	89.99(7)
C14	N3	Cu1	113.43(18)	F5	P1	F3	88.87(7)
N3	C4	C3	110.5(2)	F6	P1	F1	91.02(6)
N2	C3	C4	108.8(3)	F6	P1	F3	179.58(6)
N1	C8	C7	110.4(2)	F6	P1	F4	89.51(6)
N1	C1	C2	109.9(2)	F6	P1	F5	91.29(7)
N4	C7	C8	108.4(2)	C3	N2	Cu1	103.7(3)
N2	C2	C1	110.1(3)	C3	N2	C2	113.8(4)
N4	C6	C5	109.5(3)	C2	N2	Cu1	109.4(3)
N3	C5	C6	109.9(2)				

[1]+X,1/2-Y,+Z

Table S30 Torsion Angles for SC07.

A	B	C	D	Angle/°	A	B	C	D	Angle/°
Cu1	N1	C8	C7	-25.9(3)	C8	N1	C9	C10	-75.5(10)
Cu1	N1	C1	C2	-48.2(2)	C1	N1	C8	C7	85.5(3)
Cu1	N1	C9	C10	167.8(7)	C1	N1	C9	C10	50.6(11)
Cu1	N4	C7	C8	-50.6(3)	C1	C2	N2	Cu1	-23.8(4)
Cu1	N4	C6	C5	-24.5(4)	C1	C2	N2	C3	91.6(4)
Cu1	N3	C4	C3	-26.2(3)	C7	N4	C6	C5	90.6(4)
Cu1	N3	C5	C6	-49.3(2)	C6	N4	C7	C8	-168.8(3)
Cu1	N3	C14	C15	167.8(8)	C5	N3	C4	C3	84.5(3)
N1	C8	C7	N4	52.5(4)	C5	N3	C14	C15	50.7(13)
N1	C1	C2	N2	49.9(4)	C9	N1	C8	C7	-145.5(3)
N1	C9	C10	C11[1]	90.3(10)	C9	N1	C1	C2	73.7(5)
N1	C9	C10	C11	-95.4(10)	C9	C10	C11	C12	-174.31(16)
N4	C6	C5	N3	51.4(3)	C10	C11	C12	C13	0.2(2)
N3	C4	C3	N2	52.6(4)	C11[1]	C10	C11	C12	-0.1(3)
N3	C14	C15	C16	-94.9(11)	C11	C12	C13	C12[1]	-0.3(3)
N3	C14	C15	C16[1]	89.4(11)	C14	N3	C4	C3	-148.0(3)
C4	N3	C5	C6	-163.1(3)	C14	N3	C5	C6	74.1(5)
C4	N3	C14	C15	-74.8(12)	C14	C15	C16	C17	-174.54(17)
C4	C3	N2	Cu1	-49.9(3)	C15	C16	C17	C18	0.0(3)
C4	C3	N2	C2	-168.6(3)	C16[1]	C15	C16	C17	1.1(3)
C8	N1	C1	C2	-162.2(3)	C16	C17	C18	C17[1]	-1.1(3)

[1]+X,1/2-Y,+Z

Table S31 Hydrogen Atom Coordinates (Å×10^4) and Isotropic Displacement Parameters (Å^2×10^3) for SC07.

Atom	*x*	*y*	*z*	U(eq)
H4	3527.28	763.2	7201.1	25
H4A	-153.48	3616.08	7159.86	31
H4B	1048.41	4023.67	6264.77	31
H3A	1567.77	4587.8	8357.2	36
H3B	1515.77	3581.49	8967.18	36
H8A	5051.58	1377.89	10392.4	28
H8B	5522.35	972.33	9039.48	28
H1A	5333.74	3100.43	10593.05	30
H1B	3658.56	2832.32	10266.32	30
H7A	3209.61	404.46	9359.85	32
H7B	2605.06	1409.56	9631.7	32
H2A	3850.99	4374.06	9785.89	32
H2B	5249.34	4201.49	8970.99	32
H6A	930.44	610.55	7919.91	32
H6B	1238.8	807.1	6433.24	32
H5C	-445.43	1896.23	7019.58	28
H5D	538.72	2155.41	8309.91	28

Table S31 Hydrogen Atom Coordinates (Å×10[4]) and Isotropic Displacement Parameters (Å[2]×10[3]) for SC07.

Atom	*x*	*y*	*z*	U(eq)
H9A	6561.77	2950.19	7937.2	16
H9B	6720.49	1867.4	7985.21	16
H11	7969.55	3882.35	9720.59	34
H12	9999.69	3875.36	11214.61	43
H13	11019.39	2500.01	11952.07	44
H14A	1695.62	2043.87	4943.35	19
H14B	1588.95	3127.77	4827.66	19
H16	-654.7	1120.58	4542.21	45
H17	-2987.69	1124.01	3483.97	59
H18	-4134.59	2500	2927.42	63
H2	3639.07	4240.84	7307.86	25
H5A	5231(16)	2054(9)	5685(16)	31
H5B	4190(20)	2500	5040(20)	31

Table S32 Atomic Occupancy for SC07.

Atom	*Occupancy*	Atom	*Occupancy*	Atom	*Occupancy*
N1	0.5	N4	0.5	H4	0.5
N3	0.5	C4	0.5	H4A	0.5
H4B	0.5	C3	0.5	H3A	0.5
H3B	0.5	C8	0.5	H8A	0.5
H8B	0.5	C1	0.5	H1A	0.5
H1B	0.5	C7	0.5	H7A	0.5
H7B	0.5	C2	0.5	H2A	0.5
H2B	0.5	C6	0.5	H6A	0.5
H6B	0.5	C5	0.5	H5C	0.5
H5D	0.5	C9	0.5	H9A	0.5
H9B	0.5	C14	0.5	H14A	0.5
H14B	0.5	N2	0.5	H2	0.5

Table S33 Crystal data and structure refinement for [Zn(**1**)(OOCCH$_3$)]PF$_6$ **[ZNL1l]**

]Identification code	ZNL1l
Empirical formula	$C_{24}H_{35}F_6N_4O_2PZn$
Formula weight	621.90
Temperature/K	150
Crystal system	monoclinic
Space group	$P2_1$
a/Å	10.5603(9)
b/Å	12.2847(11)
c/Å	10.5771(9)
α/°	90
β/°	97.265(3)
γ/°	90
Volume/Å^3	1361.2(2)
Z	2
ρ_{calc}g/cm^3	1.517
μ/mm^{-1}	1.298
F(000)	644.0
Crystal size/mm^3	0.04 × 0.03 × 0.03
Radiation	synchrotron (λ = 0.7749 Å)
2Θ range for data collection/°	4.232 to 58.154
Index ranges	-13 ≤ h ≤ 13, -15 ≤ k ≤ 15, -13 ≤ l ≤ 13
Reflections collected	22785
Independent reflections	5555 [R_{int} = 0.1106, R_{sigma} = 0.0843]
Data/restraints/parameters	5555/634/447
Goodness-of-fit on F^2	1.035
Final R indexes [I>=2σ (I)]	R_1 = 0.0494, wR_2 = 0.1039
Final R indexes [all data]	R_1 = 0.0680, wR_2 = 0.1111
Largest diff. peak/hole / e Å^{-3}	0.88/-0.37
Flack parameter	0.077(9)

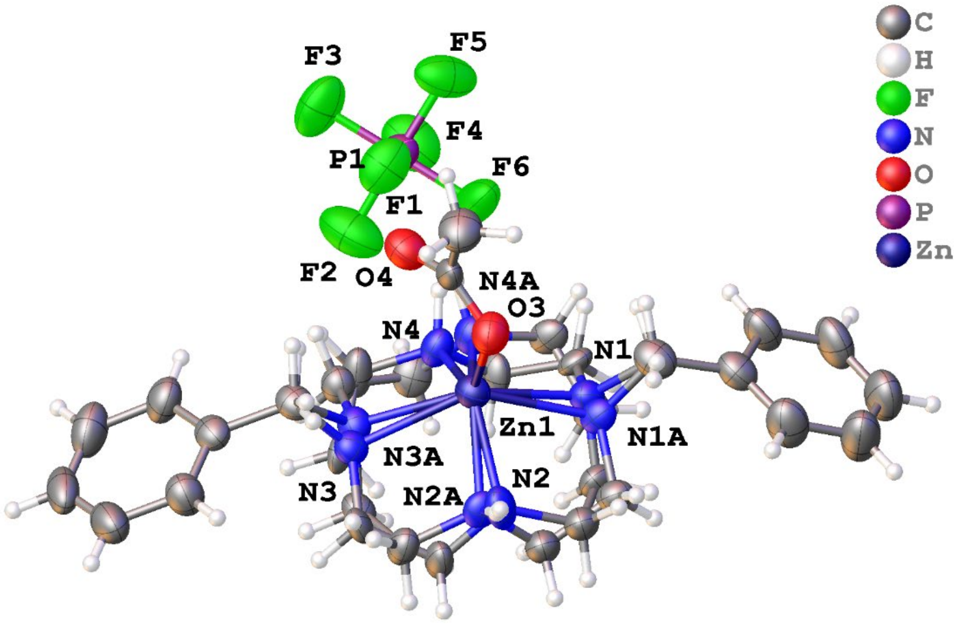

Figure S5 Asymmetric unit of [Zn(**1**)(OOCCH$_3$)]PF$_6$ **with atoms drawn as 50 % probability ellipsoids. The two disorder components are both shown.**

Table S34 Fractional Atomic Coordinates (×10[4]) and Equivalent Isotropic Displacement Parameters (Å[2]×10[3]) for ZNL1l. U_{eq} is defined as 1/3 of of the trace of the orthogonalised U_{IJ} tensor.

Atom	*x*	*y*	*z*	U(eq)
Zn1	-375.7(6)	-8624.2(6)	11549.5(6)	37.93(19)
O3	-1317(4)	-8826(4)	9864(4)	54.1(12)
O4	-1127(4)	-7082(5)	9594(5)	58.9(13)
N3A	1474(12)	-7808(9)	11755(11)	38(2)
N1A	-1624(14)	-9568(8)	12806(11)	37(3)
N4A	-853(10)	-7359(11)	12761(11)	45(3)
C23	-1440(6)	-7959(6)	9272(7)	45.8(15)
C18	4224(6)	-7775(6)	10182(7)	56.5(17)
C10	-3829(6)	-10195(6)	13109(7)	51.9(16)
C12	-5234(7)	-10041(8)	14729(9)	69(2)
C24	-2131(8)	-8155(7)	7851(7)	64(2)
C22	3575(7)	-6074(6)	10882(7)	59.3(18)
C11	-4517(6)	-9563(6)	13893(8)	58.3(18)
C9	-2971(6)	-9651(6)	12239(6)	53.3(17)
C20	5758(8)	-6359(8)	10619(8)	72(2)
C21	4822(8)	-5708(8)	10946(8)	75(2)
C13	-5308(8)	-11137(9)	14799(10)	79(3)
C7A	-1750(12)	-7719(10)	13629(12)	43(3)
C15	-3929(8)	-11293(7)	13187(9)	71(2)
C5A	1329(14)	-6809(11)	12514(14)	43(3)
C3A	2114(12)	-9725(10)	11935(13)	45(3)
C8A	-1515(11)	-8890(9)	13958(10)	41(3)
C19	5465(7)	-7397(8)	10211(8)	73(2)
C4A	2339(7)	-8587(10)	12489(8)	41(2)
C14	-4661(9)	-11778(8)	14022(11)	86(3)
C1A	-1005(9)	-10625(9)	13043(11)	42(2)
C16	1913(6)	-7541(6)	10466(6)	42.9(14)
C17	3268(6)	-7117(6)	10511(6)	43.8(14)
C6A	363(12)	-6990(10)	13441(11)	47(3)
N3	1740(20)	-8157(15)	11559(18)	37(4)
N1	-1680(20)	-9171(15)	12880(20)	41(4)
C3	1830(20)	-10031(18)	12400(30)	49(6)
C2A	432(10)	-10539(11)	13103(12)	49(3)
C1	-889(19)	-10018(15)	13600(20)	53(5)
C5	1994(17)	-7565(14)	12759(16)	41(4)
C7	-649(18)	-7542(15)	13944(17)	48(5)
C8	-1820(20)	-8243(18)	13730(20)	38(5)
C4	2430(16)	-9201(14)	11600(20)	44(4)
C6	980(20)	-6698(19)	12820(30)	56(7)
P1	-1746.8(19)	-4238.7(15)	13053.1(17)	49.6(4)
F6	-2196(5)	-5343(4)	13604(6)	96.0(18)
F2	-321(5)	-4535(6)	13582(5)	95.4(18)
F4	-1932(4)	-3638(6)	14348(4)	85.5(13)
F5	-3176(5)	-3956(4)	12540(5)	84.9(16)

Table S34 Fractional Atomic Coordinates (×10^4) and Equivalent Isotropic Displacement Parameters (Å^2×10^3) for ZNL1l. U_{eq} is defined as 1/3 of of the trace of the orthogonalised U_{IJ} tensor.

Atom	*x*	*y*	*z*	U(eq)
F1	-1550(5)	-4850(4)	11763(4)	76.2(13)
F3	-1282(6)	-3141(4)	12503(5)	90.4(16)
N4	-292(19)	-7205(17)	12708(18)	41(4)
N2	480(30)	-10200(40)	11880(30)	53(6)
N2A	755(14)	-10023(16)	11902(15)	38(3)
C2	-320(20)	-10795(15)	12720(20)	49(3)

Table S35 Anisotropic Displacement Parameters (Å^2×10^3) for ZNL1l. The Anisotropic displacement factor exponent takes the form: $-2\pi^2[h^2a^{*2}U_{11}+2hka^*b^*U_{12}+\ldots]$.

Atom	U_{11}	U_{22}	U_{33}	U_{23}	U_{13}	U_{12}
Zn1	36.9(3)	42.4(3)	34.8(3)	-0.7(4)	5.9(2)	2.4(4)
O3	53(3)	56(3)	53(3)	4(2)	6(2)	-9(2)
O4	47(3)	68(3)	60(3)	0(3)	1(2)	1(2)
N3A	38(6)	43(6)	32(5)	0(4)	6(4)	-2(5)
N1A	46(5)	35(6)	33(5)	-2(5)	14(4)	5(5)
N4A	46(6)	43(6)	48(6)	-3(4)	15(5)	-2(5)
C23	27(3)	40(3)	74(4)	19(3)	20(3)	4(3)
C18	48(4)	57(4)	65(5)	-1(4)	10(3)	-3(3)
C10	38(3)	61(4)	56(4)	-3(3)	2(3)	2(3)
C12	45(4)	81(5)	87(6)	-15(5)	28(4)	-6(4)
C24	63(4)	74(5)	52(4)	10(4)	-2(3)	-6(4)
C22	65(4)	51(4)	65(5)	9(3)	23(4)	-9(3)
C11	40(4)	53(4)	84(5)	-10(4)	15(3)	1(3)
C9	48(4)	63(5)	47(4)	-4(3)	2(3)	3(3)
C20	47(4)	98(6)	70(5)	38(4)	1(3)	-22(4)
C21	80(5)	71(5)	74(5)	7(4)	11(4)	-33(4)
C13	60(6)	79(5)	106(8)	10(5)	39(5)	-3(5)
C7A	51(8)	38(6)	43(6)	0(6)	20(5)	11(6)
C15	62(5)	58(4)	96(6)	-15(4)	28(4)	-1(4)
C5A	40(7)	41(7)	48(7)	-2(5)	11(6)	-2(6)
C3A	45(7)	37(7)	55(9)	6(6)	20(6)	0(5)
C8A	50(6)	43(6)	31(5)	2(4)	10(4)	0(5)
C19	44(4)	90(6)	86(6)	31(5)	14(4)	3(4)
C4A	29(4)	48(5)	46(5)	1(6)	1(3)	-1(5)
C14	74(5)	56(5)	138(8)	7(5)	47(5)	-1(5)
C1A	46(6)	37(6)	44(6)	7(5)	9(5)	7(5)
C16	39(3)	55(4)	34(3)	2(3)	2(2)	3(3)
C17	40(3)	54(4)	37(3)	11(3)	5(3)	1(3)
C6A	59(7)	44(7)	39(6)	-13(5)	13(5)	-4(5)
N3	36(10)	38(8)	38(8)	-2(6)	2(7)	-2(6)
N1	37(8)	37(9)	48(11)	3(7)	3(7)	3(7)
C3	37(10)	38(11)	78(17)	9(10)	27(10)	1(9)
C2A	39(6)	41(6)	67(7)	13(5)	8(5)	3(5)

Table S35 Anisotropic Displacement Parameters (Å^2×10^3) for ZNL1l. The Anisotropic displacement factor exponent takes the form: $-2\pi^2[h^2a^{*2}U_{11}+2hka^*b^*U_{12}+\ldots]$.

Atom	U_{11}	U_{22}	U_{33}	U_{23}	U_{13}	U_{12}
C1	47(11)	32(9)	78(14)	12(9)	6(9)	0(8)
C5	52(10)	32(9)	42(8)	-5(7)	13(8)	-22(8)
C7	52(11)	46(10)	51(11)	-6(8)	31(8)	-6(8)
C8	46(11)	26(9)	41(11)	13(8)	7(8)	5(8)
C4	21(8)	41(9)	69(13)	2(8)	6(8)	-6(7)
C6	57(13)	42(12)	77(19)	-11(11)	35(11)	-15(8)
P1	61.2(11)	46.2(10)	40.9(10)	-0.4(8)	5.0(8)	0.9(9)
F6	107(4)	67(3)	127(4)	39(3)	64(3)	26(3)
F2	71(3)	143(5)	70(3)	-16(3)	-1(2)	27(3)
F4	85(3)	121(4)	50(2)	-27(3)	8.9(19)	7(4)
F5	80(3)	73(4)	92(3)	-20(3)	-27(2)	17(2)
F1	111(4)	63(3)	55(3)	-14(2)	15(2)	-11(3)
F3	136(4)	50(2)	91(4)	-10(3)	37(3)	-18(3)
N4	49(10)	33(8)	45(10)	-1(7)	17(9)	-3(8)
N2	39(10)	43(13)	80(14)	-7(10)	14(8)	-12(8)
N2A	48(8)	27(7)	40(5)	-9(4)	12(5)	-2(6)
C2	39(6)	41(6)	67(7)	13(5)	8(5)	3(5)

Table S36 Bond Lengths for ZNL1l.

Atom	Atom	Length/Å	Atom	Atom	Length/Å
Zn1	O3	1.944(5)	C20	C21	1.350(13)
Zn1	N3A	2.182(14)	C20	C19	1.370(14)
Zn1	N1A	2.301(14)	C13	C14	1.380(14)
Zn1	N4A	2.115(13)	C7A	C8A	1.493(14)
Zn1	N3	2.30(2)	C15	C14	1.381(12)
Zn1	N1	2.19(3)	C5A	C6A	1.517(13)
Zn1	N4	2.13(2)	C3A	C4A	1.523(14)
Zn1	N2	2.15(5)	C3A	N2A	1.477(14)
Zn1	N2A	2.10(2)	C1A	C2A	1.514(13)
O3	C23	1.234(8)	C16	C17	1.518(9)
O4	C23	1.165(9)	C16	N3	1.41(2)
N3A	C5A	1.485(14)	N3	C5	1.459(17)
N3A	C4A	1.474(12)	N3	C4	1.474(17)
N3A	C16	1.530(13)	N1	C1	1.480(17)
N1A	C9	1.476(16)	N1	C8	1.471(18)
N1A	C8A	1.468(13)	C3	C4	1.517(18)
N1A	C1A	1.461(13)	C3	N2	1.478(19)
N4A	C7A	1.469(13)	C2A	N2A	1.497(14)
N4A	C6A	1.463(13)	C1	C2	1.505(19)
C23	C24	1.604(11)	C5	C6	1.515(19)
C18	C19	1.387(10)	C7	C8	1.501(19)
C18	C17	1.372(9)	C7	N4	1.465(17)
C10	C11	1.403(10)	C6	N4	1.477(18)
C10	C9	1.525(10)	P1	F6	1.573(5)

Table S36 Bond Lengths for ZNL1l.

Atom	Atom	Length/Å	Atom	Atom	Length/Å
C10	C15	1.356(11)	P1	F2	1.581(5)
C12	C11	1.368(11)	P1	F4	1.590(5)
C12	C13	1.351(13)	P1	F5	1.577(5)
C22	C21	1.385(11)	P1	F1	1.594(5)
C22	C17	1.367(10)	P1	F3	1.572(5)
C9	N1	1.56(3)	N2	C2	1.495(19)

Table S37 Bond Angles for ZNL1l.

Atom	Atom	Atom	Angle/°	Atom	Atom	Atom	Angle/°
O3	Zn1	N3A	119.8(3)	C20	C19	C18	119.6(8)
O3	Zn1	N1A	101.6(3)	N3A	C4A	C3A	109.7(9)
O3	Zn1	N4A	121.1(4)	C13	C14	C15	119.6(9)
O3	Zn1	N3	114.7(5)	N1A	C1A	C2A	111.7(10)
O3	Zn1	N1	105.1(6)	C17	C16	N3A	115.9(7)
O3	Zn1	N4	127.8(6)	N3	C16	C17	112.3(10)
O3	Zn1	N2	100.9(10)	C18	C17	C16	120.7(6)
O3	Zn1	N2A	105.8(5)	C22	C17	C18	118.0(7)
N3A	Zn1	N1A	138.2(4)	C22	C17	C16	121.2(7)
N4A	Zn1	N3A	83.2(4)	N4A	C6A	C5A	110.4(11)
N4A	Zn1	N1A	79.8(4)	C16	N3	Zn1	111.0(12)
N1	Zn1	N3	139.4(8)	C16	N3	C5	114.5(16)
N4	Zn1	N3	79.8(6)	C16	N3	C4	112.0(15)
N4	Zn1	N1	82.2(6)	C5	N3	Zn1	101.7(13)
N4	Zn1	N2	131.4(12)	C5	N3	C4	111.7(17)
N2	Zn1	N3	80.6(7)	C4	N3	Zn1	105.1(12)
N2	Zn1	N1	84.6(8)	C9	N1	Zn1	114.9(13)
N2A	Zn1	N3A	82.9(4)	C1	N1	Zn1	100.7(15)
N2A	Zn1	N1A	80.8(4)	C1	N1	C9	111.2(16)
N2A	Zn1	N4A	131.9(6)	C8	N1	Zn1	106.0(15)
C23	O3	Zn1	111.4(5)	C8	N1	C9	113.9(18)
C5A	N3A	Zn1	106.4(8)	C8	N1	C1	109.2(19)
C5A	N3A	C16	111.6(10)	N2	C3	C4	109.6(19)
C4A	N3A	Zn1	104.0(7)	N2A	C2A	C1A	109.2(10)
C4A	N3A	C5A	110.5(11)	N1	C1	C2	112.0(18)
C4A	N3A	C16	111.8(8)	N3	C5	C6	109.6(19)
C16	N3A	Zn1	112.2(7)	N4	C7	C8	109.1(17)
C9	N1A	Zn1	113.2(7)	N1	C8	C7	112.9(18)
C8A	N1A	Zn1	101.7(7)	N3	C4	C3	111.1(17)
C8A	N1A	C9	110.3(10)	N4	C6	C5	109.8(18)
C1A	N1A	Zn1	105.7(8)	F6	P1	F2	89.3(3)
C1A	N1A	C9	113.3(9)	F6	P1	F4	90.3(4)
C1A	N1A	C8A	112.1(11)	F6	P1	F5	89.8(3)
C7A	N4A	Zn1	112.3(8)	F6	P1	F1	89.4(3)
C6A	N4A	Zn1	105.3(7)	F2	P1	F4	91.0(3)
C6A	N4A	C7A	112.4(10)	F2	P1	F1	88.5(3)

Table S37 Bond Angles for ZNL1l.

Atom	Atom	Atom	Angle/°	Atom	Atom	Atom	Angle/°
O3	C23	C24	110.6(7)	F4	P1	F1	179.4(3)
O4	C23	O3	129.9(7)	F5	P1	F2	179.1(4)
O4	C23	C24	119.4(6)	F5	P1	F4	88.7(3)
C17	C18	C19	121.4(8)	F5	P1	F1	91.8(3)
C11	C10	C9	120.3(7)	F3	P1	F6	179.3(3)
C15	C10	C11	117.7(7)	F3	P1	F2	90.1(4)
C15	C10	C9	122.0(7)	F3	P1	F4	89.9(3)
C13	C12	C11	120.2(8)	F3	P1	F5	90.8(3)
C17	C22	C21	120.5(8)	F3	P1	F1	90.3(3)
C12	C11	C10	121.0(7)	C7	N4	Zn1	106.6(13)
N1A	C9	C10	113.6(7)	C7	N4	C6	112(2)
C10	C9	N1	117.2(11)	C6	N4	Zn1	111.4(14)
C21	C20	C19	119.2(8)	C3	N2	Zn1	107(2)
C20	C21	C22	121.2(9)	C3	N2	C2	116(2)
C12	C13	C14	120.0(9)	C2	N2	Zn1	106(2)
N4A	C7A	C8A	109.5(10)	C3A	N2A	Zn1	109.3(10)
C10	C15	C14	121.4(8)	C3A	N2A	C2A	114.2(12)
N3A	C5A	C6A	110.6(10)	C2A	N2A	Zn1	108.2(11)
N2A	C3A	C4A	109.9(11)	N2	C2	C1	111(2)
N1A	C8A	C7A	111.1(10)				

Table S38 Torsion Angles for ZNL1l.

A	B	C	D	Angle/°	A	B	C	D	Angle/°
Zn1	O3	C23	O4	-3.2(9)	C5A	N3A	C4A	C3A	159.7(10)
Zn1	O3	C23	C24	176.0(4)	C5A	N3A	C16	C17	69.0(11)
Zn1	N3A	C5A	C6A	31.2(14)	C8A	N1A	C9	C10	-62.2(11)
Zn1	N3A	C4A	C3A	45.9(9)	C8A	N1A	C1A	C2A	-77.5(14)
Zn1	N3A	C16	C17	-171.7(5)	C19	C18	C17	C22	-0.6(10)
Zn1	N1A	C9	C10	-175.4(5)	C19	C18	C17	C16	179.6(6)
Zn1	N1A	C8A	C7A	48.7(11)	C19	C20	C21	C22	0.4(13)
Zn1	N1A	C1A	C2A	32.4(12)	C4A	N3A	C5A	C6A	-81.1(15)
Zn1	N4A	C7A	C8A	32.0(13)	C4A	N3A	C16	C17	-55.3(11)
Zn1	N4A	C6A	C5A	47.8(11)	C4A	C3A	N2A	Zn1	33.7(13)
Zn1	N3	C5	C6	-51.1(17)	C4A	C3A	N2A	C2A	-87.6(17)
Zn1	N3	C4	C3	-36(2)	C1A	N1A	C9	C10	64.3(11)
Zn1	N1	C1	C2	-48.7(19)	C1A	N1A	C8A	C7A	161.2(11)
Zn1	N1	C8	C7	-31(2)	C1A	C2A	N2A	Zn1	48.2(13)
Zn1	N2	C2	C1	-31(3)	C1A	C2A	N2A	C3A	170.2(14)
N3A	C5A	C6A	N4A	-55.2(15)	C16	N3A	C5A	C6A	153.9(11)
N3A	C16	C17	C18	104.3(8)	C16	N3A	C4A	C3A	-75.4(12)
N3A	C16	C17	C22	-75.5(9)	C16	N3	C5	C6	69(2)
N1A	C1A	C2A	N2A	-55.4(16)	C16	N3	C4	C3	-156.1(19)
N4A	C7A	C8A	N1A	-56.9(14)	C17	C18	C19	C20	2.4(12)
C10	C9	N1	Zn1	171.2(7)	C17	C22	C21	C20	1.5(12)
C10	C9	N1	C1	58(2)	C17	C16	N3	Zn1	173.9(6)

Table S38 Torsion Angles for ZNL1l.

A	B	C	D	Angle/°	A	B	C	D	Angle/°
C10	C9	N1	C8	-66.2(19)	C17	C16	N3	C5	59.6(17)
C10	C15	C14	C13	-0.1(15)	C17	C16	N3	C4	-69.0(16)
C12	C13	C14	C15	0.8(16)	C6A	N4A	C7A	C8A	-86.5(14)
C11	C10	C9	N1A	94.6(9)	N3	C16	C17	C18	80.9(10)
C11	C10	C9	N1	74.4(11)	N3	C16	C17	C22	-98.9(10)
C11	C10	C15	C14	-1.1(13)	N3	C5	C6	N4	58(3)
C11	C12	C13	C14	-0.2(15)	N1	C1	C2	N2	58(3)
C9	N1A	C8A	C7A	-71.6(13)	C3	N2	C2	C1	88(4)
C9	N1A	C1A	C2A	156.9(10)	C1	N1	C8	C7	77(3)
C9	C10	C11	C12	-176.3(7)	C5	N3	C4	C3	74(2)
C9	C10	C15	C14	176.9(8)	C5	C6	N4	Zn1	-31(3)
C9	N1	C1	C2	73(2)	C5	C6	N4	C7	88(3)
C9	N1	C8	C7	-158.5(17)	C8	N1	C1	C2	-160.0(19)
C21	C22	C17	C18	-1.3(11)	C8	C7	N4	Zn1	-46.5(19)
C21	C22	C17	C16	178.5(6)	C8	C7	N4	C6	-168.8(18)
C21	C20	C19	C18	-2.3(12)	C4	N3	C5	C6	-162.7(17)
C13	C12	C11	C10	-1.1(13)	C4	C3	N2	Zn1	-48(3)
C7A	N4A	C6A	C5A	170.5(11)	C4	C3	N2	C2	-166(3)
C15	C10	C11	C12	1.7(12)	N4	C7	C8	N1	54(2)
C15	C10	C9	N1A	-83.4(10)	N2	C3	C4	N3	58(3)
C15	C10	C9	N1	-103.6(11)	N2A	C3A	C4A	N3A	-55.5(13)

Table S39 Hydrogen Atom Coordinates (Å×10^4) and Isotropic Displacement Parameters (Å^2×10^3) for ZNL1l.

Atom	*x*	*y*	*z*	U(eq)
H4A	-1241.45	-6760.11	12236.22	54
H18	4035.92	-8489.67	9934.6	68
H12	-5672.38	-9610.24	15251.58	83
H24A	-1508.96	-8375.99	7313.77	96
H24B	-2532	-7492.97	7528.57	96
H24C	-2764.27	-8714.83	7859.69	96
H22	2941.64	-5606.36	11092.38	71
H11	-4483.86	-8808.24	13842.88	70
H9AA	-3014.32	-10061.24	11451.55	64
H9AB	-3294.27	-8925.07	12028.78	64
H9BC	-3451.69	-9065.89	11786.61	64
H9BD	-2784.12	-10180.08	11608.18	64
H20	6592.89	-6105	10670.65	86
H21	5017.67	-5001.33	11218.97	90
H13	-5794.02	-11458.98	15370.82	95
H7AA	-2620.34	-7624.73	13225.4	51
H7AB	-1637.4	-7282.56	14399.53	51
H15	-3494.06	-11729.26	12665.82	85
H5AA	1050.28	-6212.04	11946.55	51
H5AB	2147.18	-6614.19	12982.77	51

Table S39 Hydrogen Atom Coordinates (Å×10^4) and Isotropic Displacement Parameters (Å^2×10^3) for ZNL1l.

Atom	*x*	*y*	*z*	U(eq)
H3AA	2352.04	-9746.11	11079.51	53
H3AB	2641.1	-10245.49	12453.36	53
H8AA	-668.15	-8971.34	14422.76	49
H8AB	-2129.69	-9133.91	14505.76	49
H19	6093.89	-7846.19	9954.09	87
H4AA	2184.83	-8584.47	13374.29	50
H4AB	3218.29	-8373.81	12455.93	50
H14	-4718.79	-12532.4	14060.83	104
H1AA	-1325.9	-11125.33	12368.17	50
H1AB	-1217.46	-10917.12	13841.38	50
H16A	1833.28	-8192.97	9945.48	51
H16B	1336.44	-7000.81	10043.94	51
H16C	1326.02	-6929.77	10396.43	51
H16D	1711.92	-7990.2	9713.64	51
H6AA	686.42	-7530.08	14068.8	56
H6AB	234.62	-6315.81	13885.45	56
H3A	2290.3	-10714.28	12406.08	59
H3B	1878.69	-9774.41	13276.25	59
H2AA	771.47	-10102.99	13832.22	59
H2AB	811.18	-11258.47	13199.35	59
H1A	-1412.48	-10423.14	14121.58	63
H1B	-207.47	-9672.3	14154.36	63
H5A	2830.54	-7228.66	12819.12	49
H5B	1989.32	-8065.17	13467.73	49
H7A	-816.66	-6906.09	14439.45	57
H7B	45.96	-7947.09	14414.35	57
H8A	-2014.7	-8514.07	14544.94	45
H8B	-2536.66	-7800.8	13364.43	45
H4B	3312.16	-9083.62	11955.08	52
H4C	2420.73	-9478.96	10741.58	52
H6A	1146.1	-6312.19	13629.28	68
H6B	1019.79	-6175.99	12141.47	68
H4	-923.17	-6679.64	12319.23	50
H2	444.91	-10587.7	11062.32	64
H2A	534.12	-10534.93	11198.08	46
H2B	202.67	-11326.55	13225.75	59
H2C	-998.33	-11180.59	12202.69	59

Table S40 Atomic Occupancy for ZNL1l.

Atom	*Occupancy*	Atom	*Occupancy*	Atom	*Occupancy*
N3A	0.630(7)	N1A	0.630(7)	N4A	0.630(7)
H4A	0.630(7)	H9AA	0.630(7)	H9AB	0.630(7)
H9BC	0.370(7)	H9BD	0.370(7)	C7A	0.630(7)
H7AA	0.630(7)	H7AB	0.630(7)	C5A	0.630(7)

Table S40 Atomic Occupancy for ZNL1l.

Atom	*Occupancy*	Atom	*Occupancy*	Atom	*Occupancy*
H5AA	0.630(7)	H5AB	0.630(7)	C3A	0.630(7)
H3AA	0.630(7)	H3AB	0.630(7)	C8A	0.630(7)
H8AA	0.630(7)	H8AB	0.630(7)	C4A	0.630(7)
H4AA	0.630(7)	H4AB	0.630(7)	C1A	0.630(7)
H1AA	0.630(7)	H1AB	0.630(7)	H16A	0.630(7)
H16B	0.630(7)	H16C	0.370(7)	H16D	0.370(7)
C6A	0.630(7)	H6AA	0.630(7)	H6AB	0.630(7)
N3	0.370(7)	N1	0.370(7)	C3	0.370(7)
H3A	0.370(7)	H3B	0.370(7)	C2A	0.630(7)
H2AA	0.630(7)	H2AB	0.630(7)	C1	0.370(7)
H1A	0.370(7)	H1B	0.370(7)	C5	0.370(7)
H5A	0.370(7)	H5B	0.370(7)	C7	0.370(7)
H7A	0.370(7)	H7B	0.370(7)	C8	0.370(7)
H8A	0.370(7)	H8B	0.370(7)	C4	0.370(7)
H4B	0.370(7)	H4C	0.370(7)	C6	0.370(7)
H6A	0.370(7)	H6B	0.370(7)	N4	0.370(7)
H4	0.370(7)	N2	0.370(7)	H2	0.370(7)
N2A	0.630(7)	H2A	0.630(7)	C2	0.370(7)
H2B	0.370(7)	H2C	0.370(7)		

Table S41 Crystal data and structure refinement for [Co(**2**)(μ-OOCCH$_3$)]PF$_6$. **[MLB07 C2onC]**

Identification code	MLB07 C2onC
Empirical formula	$C_{26}H_{39}CoF_6N_4O_2P$
Formula weight	643.51
Temperature/K	150
Crystal system	monoclinic
Space group	C2/c
a/Å	21.981(2)
b/Å	12.0238(10)
c/Å	22.357(2)
α/°	90
β/°	99.330(4)
γ/°	90
Volume/Å^3	5830.5(10)
Z	8
ρ_{calc}g/cm^3	1.466
μ/mm^{-1}	0.902
F(000)	2680.0
Crystal size/mm^3	0.09 × 0.05 × 0.05
Radiation	synchrotron (λ = 0.7749 Å)
2Θ range for data collection/°	4.094 to 62.252
Index ranges	-29 ≤ h ≤ 29, -15 ≤ k ≤ 16, -29 ≤ l ≤ 29
Reflections collected	51124
Independent reflections	7238 [R_{int} = 0.0518, R_{sigma} = 0.0263]
Data/restraints/parameters	7238/273/417
Goodness-of-fit on F^2	1.043
Final R indexes [I>=2σ (I)]	R_1 = 0.0627, wR_2 = 0.1647
Final R indexes [all data]	R_1 = 0.0711, wR_2 = 0.1713
Largest diff. peak/hole / e Å^{-3}	1.14/-0.71

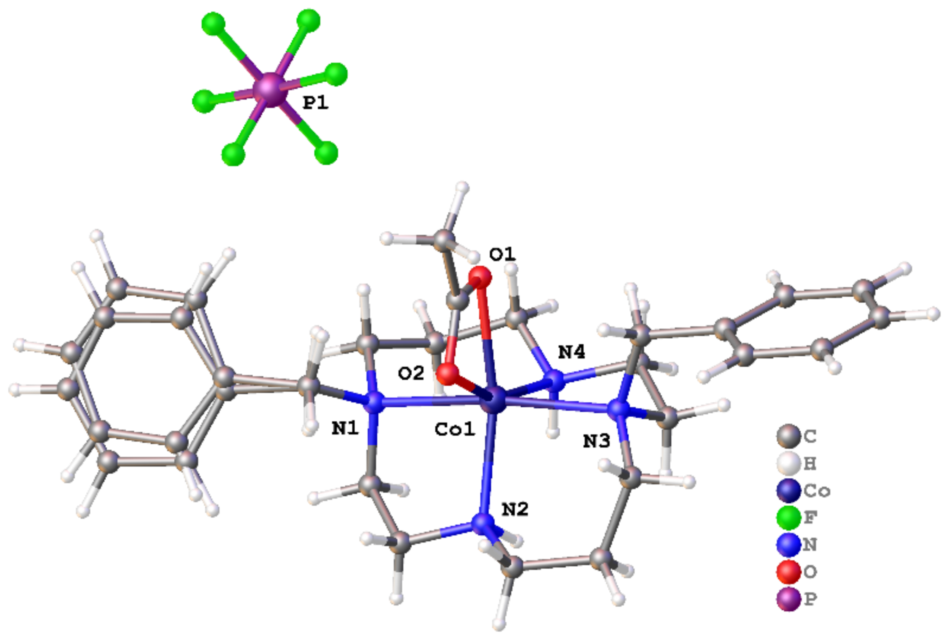

Figure S6: Asymmetric unit of [Co(**2**)(μ-OOCCH$_3$)]PF$_6$ **with atoms shown as spheres of arbitrary radius. Minor disorder is represented in full.**

Table S42 Fractional Atomic Coordinates (×10[4]) and Equivalent Isotropic Displacement Parameters (Å[2]×10[3]) for MLB07 C2onC. U_{eq} is defined as 1/3 of of the trace of the orthogonalised U_{IJ} tensor.

Atom	*x*	*y*	*z*	U(eq)
Co1	6270.6(2)	5934.3(3)	4384.0(2)	22.40(12)
O1	5875.2(11)	6055(2)	5195.8(11)	36.2(5)
O2	6455.9(10)	7381(2)	4930.9(11)	33.8(5)
N1	7087.1(13)	4955(2)	4845.8(13)	33.5(6)
N2	6831.6(12)	6136(2)	3716.4(13)	31.7(6)
N3	5469.3(13)	6772(2)	3797.5(12)	33.7(6)
N4	5812.8(12)	4475(2)	4025.4(13)	34.4(6)
C1	7445.6(15)	4766(3)	4351.3(17)	37.9(7)
C2	7461.2(16)	5817(3)	3978.7(17)	39.1(8)
C3	6792.7(18)	7265(3)	3442.6(17)	41.2(8)
C4	6169.3(19)	7506(3)	3079.0(17)	44.0(8)
C5	5644.9(18)	7711(3)	3439.5(16)	40.3(8)
C6	5188.0(16)	5864(3)	3402.5(16)	38.8(8)
C7	5176.6(16)	4794(3)	3754.3(17)	41.4(8)
C8	5824.8(17)	3593(3)	4486.2(19)	43.8(8)
C9	6469.8(18)	3140(3)	4691(2)	46.1(9)
C10	6913.1(19)	3893(3)	5106.5(18)	43.1(8)
C11	7449.1(16)	5628(3)	5346.8(15)	35.8(7)
C12	8021(9)	5030(30)	5669(11)	51(5)
C13	8566(8)	5000(30)	5434(12)	47(4)
C14	9093(8)	4493(17)	5758(13)	54(4)
C15	9078(10)	4049(14)	6343(12)	55(4)
C16	8549(11)	4112(18)	6598(10)	60(4)
C17	8033(11)	4640(20)	6270(10)	55(4)
C18	5023.1(16)	7208(4)	4184.1(16)	43.6(8)
C19	4430.3(17)	7715(4)	3858.0(17)	45.6(9)
C20	4392(2)	8840(4)	3738(2)	59.5(12)
C21	3847(2)	9312(5)	3438(3)	70.8(16)
C22	3342.7(19)	8648(5)	3265(2)	61.9(13)
C23	3361.0(18)	7552(4)	3380.2(19)	53.1(10)
C24	3908.5(18)	7059(4)	3682.4(18)	49.7(9)
C25	6101.4(13)	7012(2)	5282.6(13)	26.4(6)
C26	5948.7(16)	7735(3)	5786.0(14)	34.8(7)
P1	6524.1(4)	6086.0(8)	7361.3(4)	37.5(2)
F1	6004.5(14)	5227(3)	7485.9(15)	81.6(10)
F2	6637.2(13)	6359(2)	8070.0(10)	59.3(7)
F3	7019.1(14)	5119(3)	7509.8(13)	70.9(8)
F4	7015.6(14)	6959(3)	7258.8(14)	76.8(9)
F5	6405.0(16)	5749(2)	6667.3(11)	68.1(8)
F6	6012.6(13)	7030(3)	7238.2(12)	66.9(8)
C12A	7982(6)	5070(20)	5753(8)	34(3)
C17A	7868(9)	4623(18)	6313(6)	45(3)
C16A	8352(10)	4109(15)	6705(7)	52(3)
C15A	8911(9)	3976(13)	6533(9)	55(3)

Table S42 Fractional Atomic Coordinates (×10^4) and Equivalent Isotropic Displacement Parameters (Å^2×10^3) for MLB07 C2onC. U_{eq} is defined as 1/3 of of the trace of the orthogonalised U_{IJ} tensor.

Atom	*x*	*y*	*z*	U(eq)
C14A	9032(6)	4396(15)	5973(10)	50(3)
C13A	8559(7)	4950(30)	5591(10)	43(3)

Table S43 Anisotropic Displacement Parameters (Å^2×10^3) for MLB07 C2onC. The Anisotropic displacement factor exponent takes the form: -2π^2[h^2a*^2U11+2hka*b*U12+…].

Atom	U_{11}	U_{22}	U_{33}	U_{23}	U_{13}	U_{12}
Co1	22.9(2)	25.4(2)	20.10(19)	-2.31(14)	7.20(14)	-5.70(14)
O1	39.2(12)	40.5(13)	30.8(11)	-5.0(9)	12.0(9)	-11.2(10)
O2	32.4(11)	35.0(12)	35.4(12)	-7.2(9)	9.8(9)	-6.1(9)
N1	36.1(14)	30.5(13)	33.4(14)	-3.8(11)	3.8(11)	-4.2(11)
N2	33.3(13)	30.8(13)	34.3(14)	-7.1(10)	15.4(11)	-8.6(10)
N3	33.0(13)	40.3(15)	28.3(13)	-6.5(11)	6.4(10)	-0.5(11)
N4	29.3(13)	39.8(15)	34.9(14)	-5.7(12)	7.9(11)	-9.1(11)
C1	29.4(15)	39.3(17)	45.4(19)	-9.3(15)	7.4(14)	0.4(13)
C2	31.8(16)	45.4(19)	43.5(19)	-7.8(15)	16.1(14)	-6.5(14)
C3	55(2)	35.3(17)	38.8(18)	-0.4(14)	23.2(16)	-12.6(15)
C4	58(2)	37.6(18)	40.6(19)	4.0(15)	20.5(17)	-3.7(16)
C5	53(2)	33.4(17)	35.1(17)	-4.3(14)	7.7(15)	6.6(15)
C6	34.7(16)	49(2)	31.7(16)	-14.9(14)	2.2(13)	-3.9(14)
C7	31.6(16)	50(2)	41.7(19)	-4.6(16)	4.9(14)	-12.4(15)
C8	41.2(18)	36.0(18)	54(2)	2.4(16)	8.3(16)	-14.6(15)
C9	46(2)	34.1(17)	55(2)	-1.0(16)	1.2(17)	-9.7(15)
C10	49(2)	33.9(17)	42.1(19)	5.1(14)	-3.7(16)	-3.0(15)
C11	38.4(17)	32.1(15)	34.8(16)	-2.7(13)	-0.7(13)	-3.2(13)
C12	55(7)	38(9)	54(8)	0(8)	-7(5)	-3(6)
C13	42(5)	34(6)	59(10)	-2(8)	-13(5)	-3(4)
C14	54(6)	28(5)	70(10)	-2(6)	-18(5)	-5(4)
C15	58(7)	32(5)	63(9)	1(6)	-24(6)	4(5)
C16	55(9)	47(6)	68(8)	6(6)	-19(6)	-3(8)
C17	59(8)	44(7)	60(7)	8(5)	-4(6)	-3(7)
C18	34.6(17)	66(2)	30.4(17)	-10.4(16)	6.3(13)	5.2(17)
C19	33.6(17)	64(2)	38.7(19)	-18.4(17)	4.0(14)	6.6(17)
C20	39(2)	58(3)	76(3)	-32(2)	-6.6(19)	6.7(18)
C21	51(2)	68(3)	87(4)	-40(3)	-7(2)	20(2)
C22	36(2)	86(3)	61(3)	-30(3)	0.3(18)	15(2)
C23	34.7(18)	79(3)	46(2)	-14(2)	6.6(16)	-4.2(19)
C24	38.2(19)	68(3)	44(2)	-7.2(19)	10.6(16)	-5.4(18)
C25	28.8(14)	30.1(14)	20.2(13)	-0.3(10)	3.3(10)	-1.4(11)
C26	41.9(17)	37.7(17)	26.9(15)	-5.2(13)	11.6(13)	-2.8(14)
P1	39.3(5)	44.0(5)	27.3(4)	9.9(3)	-0.7(3)	-14.8(4)
F1	67.9(18)	90(2)	82(2)	23.2(17)	-4.3(15)	-43.0(17)
F2	71.5(16)	73.8(17)	29.1(11)	7.7(11)	-2.2(10)	-6.6(14)

Table S43 Anisotropic Displacement Parameters (Å^2×10^3) for MLB07 C2onC. The Anisotropic displacement factor exponent takes the form: -2π^2[h^{2}a*^{2}U$_{11}$+2hka*b*U$_{12}$+…].

Atom	U11	U22	U33	U23	U13	U12
F3	78.1(19)	78.1(19)	53.4(15)	7.7(14)	0.9(13)	26.5(16)
F4	74.5(18)	83(2)	76.8(19)	15.4(16)	23.1(15)	-43.8(16)
F5	106(2)	59.7(16)	31.8(12)	1.6(11)	-8.0(13)	-1.3(15)
F6	68.6(17)	82(2)	47.9(14)	8.7(13)	3.4(12)	21.4(15)
C12A	43(5)	19(5)	35(5)	-7(4)	-11(3)	-3(4)
C17A	54(6)	37(5)	35(4)	1(4)	-14(4)	-9(5)
C16A	60(7)	38(4)	53(5)	6(4)	-6(5)	-4(6)
C15A	60(7)	39(5)	60(7)	7(5)	-11(6)	-3(6)
C14A	43(4)	37(5)	62(8)	8(6)	-15(5)	-5(4)
C13A	43(5)	30(5)	49(8)	-1(6)	-8(4)	-3(3)

Table S44 Bond Lengths for MLB07 C2onC.

Atom	Atom	Length/Å	Atom	Atom	Length/Å
Co1	O1	2.141(2)	C12	C13	1.385(13)
Co1	O2	2.127(2)	C12	C17	1.418(13)
Co1	N1	2.252(3)	C13	C14	1.402(12)
Co1	N2	2.099(3)	C14	C15	1.418(15)
Co1	N3	2.256(3)	C15	C16	1.377(16)
Co1	N4	2.115(3)	C16	C17	1.402(13)
Co1	C25	2.469(3)	C18	C19	1.515(5)
O1	C25	1.256(4)	C19	C20	1.378(7)
O2	C25	1.273(4)	C19	C24	1.395(6)
N1	C1	1.476(4)	C20	C21	1.396(6)
N1	C10	1.480(4)	C21	C22	1.370(7)
N1	C11	1.501(4)	C22	C23	1.342(8)
N2	C2	1.464(5)	C23	C24	1.412(6)
N2	C3	1.486(4)	C25	C26	1.502(4)
N3	C5	1.471(5)	P1	F1	1.597(3)
N3	C6	1.476(4)	P1	F2	1.597(2)
N3	C18	1.503(4)	P1	F3	1.591(3)
N4	C7	1.482(4)	P1	F4	1.550(3)
N4	C8	1.476(5)	P1	F5	1.584(3)
C1	C2	1.517(5)	P1	F6	1.590(3)
C3	C4	1.504(6)	C12A	C17A	1.420(11)
C4	C5	1.529(5)	C12A	C13A	1.380(11)
C6	C7	1.510(5)	C17A	C16A	1.407(11)
C8	C9	1.518(6)	C16A	C15A	1.355(15)
C9	C10	1.530(5)	C15A	C14A	1.415(14)
C11	C12	1.525(12)	C14A	C13A	1.405(11)
C11	C12A	1.518(9)			

Table S45 Bond Angles for MLB07 C2onC.

Atom	Atom	Atom	Angle/°	Atom	Atom	Atom	Angle/°
O1	Co1	N1	92.86(10)	N1	C10	C9	116.8(3)
O1	Co1	N3	94.38(10)	N1	C11	C12	113.2(16)
O1	Co1	C25	30.57(9)	N1	C11	C12A	118.2(11)
O2	Co1	O1	61.53(9)	C13	C12	C11	122.1(13)
O2	Co1	N1	95.73(10)	C13	C12	C17	117.9(11)
O2	Co1	N3	91.57(10)	C17	C12	C11	119.3(13)
O2	Co1	C25	31.04(9)	C12	C13	C14	120.6(13)
N1	Co1	N3	171.54(10)	C13	C14	C15	119.9(12)
N1	Co1	C25	96.62(10)	C16	C15	C14	120.6(10)
N2	Co1	O1	164.03(10)	C15	C16	C17	118.4(12)
N2	Co1	O2	103.56(10)	C16	C17	C12	122.2(13)
N2	Co1	N1	82.53(11)	N3	C18	C19	117.0(3)
N2	Co1	N3	91.62(11)	C20	C19	C18	120.6(4)
N2	Co1	N4	97.15(11)	C20	C19	C24	118.6(4)
N2	Co1	C25	134.54(10)	C24	C19	C18	120.8(4)
N3	Co1	C25	91.84(10)	C19	C20	C21	120.8(4)
N4	Co1	O1	98.29(10)	C22	C21	C20	119.4(5)
N4	Co1	O2	158.64(10)	C23	C22	C21	121.4(4)
N4	Co1	N1	92.01(11)	C22	C23	C24	120.0(4)
N4	Co1	N3	82.62(11)	C19	C24	C23	119.7(4)
N4	Co1	C25	128.23(10)	O1	C25	Co1	60.10(16)
C25	O1	Co1	89.33(18)	O1	C25	O2	119.4(3)
C25	O2	Co1	89.47(18)	O1	C25	C26	121.0(3)
C1	N1	Co1	102.7(2)	O2	C25	Co1	59.49(15)
C1	N1	C10	111.4(3)	O2	C25	C26	119.6(3)
C1	N1	C11	111.1(3)	C26	C25	Co1	174.0(2)
C10	N1	Co1	113.2(2)	F1	P1	F2	87.74(17)
C10	N1	C11	108.1(3)	F3	P1	F1	88.58(19)
C11	N1	Co1	110.3(2)	F3	P1	F2	87.24(15)
C2	N2	Co1	108.1(2)	F4	P1	F1	177.4(2)
C2	N2	C3	113.1(3)	F4	P1	F2	90.43(16)
C3	N2	Co1	113.4(2)	F4	P1	F3	93.22(19)
C5	N3	Co1	114.2(2)	F4	P1	F5	92.10(17)
C5	N3	C6	111.3(3)	F4	P1	F6	88.95(19)
C5	N3	C18	107.4(3)	F5	P1	F1	89.78(18)
C6	N3	Co1	103.2(2)	F5	P1	F2	176.96(16)
C6	N3	C18	110.7(3)	F5	P1	F3	90.94(16)
C18	N3	Co1	110.0(2)	F5	P1	F6	90.73(15)
C7	N4	Co1	107.4(2)	F6	P1	F1	89.20(19)
C8	N4	Co1	112.2(2)	F6	P1	F2	90.99(15)
C8	N4	C7	112.4(3)	F6	P1	F3	177.21(17)
N1	C1	C2	110.2(3)	C17A	C12A	C11	117.7(10)
N2	C2	C1	109.5(3)	C13A	C12A	C11	122.6(10)
N2	C3	C4	112.7(3)	C13A	C12A	C17A	119.6(9)
C3	C4	C5	116.4(3)	C16A	C17A	C12A	119.5(10)
N3	C5	C4	116.7(3)	C15A	C16A	C17A	120.1(11)

Table S45 Bond Angles for MLB07 C2onC.

Atom	Atom	Atom	Angle/°	Atom	Atom	Atom	Angle/°
N3	C6	C7	111.1(3)	C16A	C15A	C14A	121.3(9)
N4	C7	C6	109.8(3)	C13A	C14A	C15A	118.7(10)
N4	C8	C9	112.2(3)	C12A	C13A	C14A	120.6(10)
C8	C9	C10	116.3(3)				

Table S46 Torsion Angles for MLB07 C2onC.

A	B	C	D	Angle/°	A	B	C	D	Angle/°
Co1	O1	C25	O2	-5.4(3)	C7	N4	C8	C9	171.0(3)
Co1	O1	C25	C26	173.2(3)	C8	N4	C7	C6	166.8(3)
Co1	O2	C25	O1	5.5(3)	C8	C9	C10	N1	-64.9(5)
Co1	O2	C25	C26	-173.2(3)	C10	N1	C1	C2	163.9(3)
Co1	N1	C1	C2	42.4(3)	C10	N1	C11	C12	57.4(10)
Co1	N1	C10	C9	49.8(4)	C10	N1	C11	C12A	49.8(8)
Co1	N1	C11	C12	-178.3(10)	C11	N1	C1	C2	-75.5(3)
Co1	N1	C11	C12A	174.1(7)	C11	N1	C10	C9	172.4(3)
Co1	N2	C2	C1	42.6(3)	C11	C12	C13	C14	177(3)
Co1	N2	C3	C4	-66.7(3)	C11	C12	C17	C16	-178(3)
Co1	N3	C5	C4	49.0(4)	C11	C12A	C17A	C16A	-179.4(19)
Co1	N3	C6	C7	40.7(3)	C11	C12A	C13A	C14A	-178(2)
Co1	N3	C18	C19	-175.2(3)	C12	C13	C14	C15	-3(4)
Co1	N4	C7	C6	42.9(3)	C13	C12	C17	C16	-8(5)
Co1	N4	C8	C9	-67.8(4)	C13	C14	C15	C16	0(3)
N1	C1	C2	N2	-60.1(4)	C14	C15	C16	C17	-1(3)
N1	C11	C12	C13	81(4)	C15	C16	C17	C12	6(4)
N1	C11	C12	C17	-109(3)	C17	C12	C13	C14	7(5)
N1	C11	C12A	C17A	-96(2)	C18	N3	C5	C4	171.3(3)
N1	C11	C12A	C13A	82(3)	C18	N3	C6	C7	-76.9(4)
N2	C3	C4	C5	72.9(4)	C18	C19	C20	C21	-180.0(4)
N3	C6	C7	N4	-59.1(4)	C18	C19	C24	C23	179.9(3)
N3	C18	C19	C20	-91.7(5)	C19	C20	C21	C22	0.5(8)
N3	C18	C19	C24	89.4(5)	C20	C19	C24	C23	1.0(6)
N4	C8	C9	C10	73.9(5)	C20	C21	C22	C23	0.1(8)
C1	N1	C10	C9	-65.3(4)	C21	C22	C23	C24	-0.2(7)
C1	N1	C11	C12	-65.1(10)	C22	C23	C24	C19	-0.3(6)
C1	N1	C11	C12A	-72.7(8)	C24	C19	C20	C21	-1.1(7)
C2	N2	C3	C4	169.8(3)	C12A	C17A	C16A	C15A	-4(3)
C3	N2	C2	C1	169.1(3)	C17A	C12A	C13A	C14A	0(4)
C3	C4	C5	N3	-64.4(4)	C17A	C16A	C15A	C14A	3(3)
C5	N3	C6	C7	163.7(3)	C16A	C15A	C14A	C13A	0(3)
C5	N3	C18	C19	59.9(4)	C15A	C14A	C13A	C12A	-1(4)
C6	N3	C5	C4	-67.4(4)	C13A	C12A	C17A	C16A	3(4)
C6	N3	C18	C19	-61.8(5)					

Table S47 Hydrogen Atom Coordinates (Å×10[4]) and Isotropic Displacement Parameters (Å[2]×10[3]) for MLB07 C2onC.

Atom	*x*	*y*	*z*	U(eq)
H2	6684.84	5595.82	3384.12	38
H4	6027.07	4189.96	3694.37	41
H1A	7255.95	4155.08	4087.77	45
H1B	7871.51	4542.38	4523.93	45
H2A	7658.68	6424.28	4239.58	47
H2B	7706.68	5686.71	3651.14	47
H3A	7109.39	7335.34	3176.42	49
H3B	6884.63	7825.28	3769.25	49
H4A	6051.94	6872.8	2801.46	53
H4B	6206.71	8169.77	2825.16	53
H5A	5763.84	8342.12	3718.5	48
H5B	5276.12	7943.96	3151.8	48
H6A	5426.22	5750.53	3067.62	47
H6B	4761.77	6072.01	3222.68	47
H7A	4923.52	4894.05	4078.57	50
H7B	4988.26	4196.42	3480.72	50
H8A	5550.72	2977.34	4316.33	53
H8B	5663.43	3894.43	4841.62	53
H9A	6655.25	2976.09	4325.32	55
H9B	6434.37	2426.93	4903.33	55
H10A	6723.95	4065.78	5468.54	52
H10B	7295.22	3467.36	5246.51	52
H11A	7179.7	5813.7	5647.05	43
H11B	7576.56	6334.83	5176.51	43
H11C	7158.12	5903.51	5607.7	43
H11D	7614.15	6287.2	5161.44	43
H13	8583.67	5326.81	5050.4	57
H14	9460.27	4447.41	5585.88	65
H15	9435.58	3703.75	6560.44	66
H16	8535.24	3803.15	6986.57	72
H17	7678.78	4749.97	6457.17	67
H18A	5238.54	7776.51	4460.99	52
H18B	4913.85	6588.08	4438.26	52
H20	4741.92	9299.42	3861.34	71
H21	3826.23	10086.65	3354.07	85
H22	2972.28	8970.62	3060.37	74
H23	3005.23	7107.67	3257.92	64
H24	3921.61	6284.12	3765.84	60
H26A	5807.84	8463.8	5622.51	52
H26B	6316.87	7829.47	6093.68	52
H26C	5621.78	7383.71	5970.18	52
H17A	7468.93	4671.78	6421.22	53
H16A	8286.51	3854.38	7092.12	62
H15A	9226.87	3592.61	6792.76	66
H14A	9426.46	4302.5	5857.44	60

Table S47 Hydrogen Atom Coordinates (Å×10[4]) and Isotropic Displacement Parameters (Å[2]×10[3]) for MLB07 C2onC.

**Atom**	***x***	***y***	***z***	**U(eq)**
H13A	8636.18	5255.1	5217.7	51

Table S48 Atomic Occupancy for MLB07 C2onC.

**Atom**	***Occupancy***	**Atom**	***Occupancy***	**Atom**	***Occupancy***
H11A	0.47(4)	H11B	0.47(4)	H11C	0.53(4)
H11D	0.53(4)	C12	0.47(4)	C13	0.47(4)
H13	0.47(4)	C14	0.47(4)	H14	0.47(4)
C15	0.47(4)	H15	0.47(4)	C16	0.47(4)
H16	0.47(4)	C17	0.47(4)	H17	0.47(4)
C12A	0.53(4)	C17A	0.53(4)	H17A	0.53(4)
C16A	0.53(4)	H16A	0.53(4)	C15A	0.53(4)
H15A	0.53(4)	C14A	0.53(4)	H14A	0.53(4)
C13A	0.53(4)	H13A	0.53(4)		

Table S49 Crystal data and structure refinement for [Cu(**2**)](PF_6)$_2$**. [LE07 P1]**

Identification code	LE07 P1
Empirical formula	$C_{24}H_{36}CuF_{12}N_4P_2$
Formula weight	734.05
Temperature/K	150
Crystal system	triclinic
Space group	P-1
a/Å	8.8640(8)
b/Å	9.4180(8)
c/Å	10.1630(9)
α/°	100.323(2)
β/°	113.889(2)
γ/°	106.364(2)
Volume/Å^3	701.48(11)
Z	1
ρ_{calc}g/cm^3	1.738
μ/mm^{-1}	1.256
F(000)	375.0
Crystal size/mm^3	0.06 × 0.05 × 0.02
Radiation	Synchrotron (λ = 0.7749 Å)
2Θ range for data collection/°	5.072 to 62.378
Index ranges	-11 ≤ h ≤ 11, -12 ≤ k ≤ 12, -13 ≤ l ≤ 13
Reflections collected	12604
Independent reflections	3483 [R_{int} = 0.0283, R_{sigma} = 0.0255]
Data/restraints/parameters	3483/0/196
Goodness-of-fit on F^2	1.044
Final R indexes [I>=2σ (I)]	R_1 = 0.0272, wR_2 = 0.0686
Final R indexes [all data]	R_1 = 0.0304, wR_2 = 0.0703
Largest diff. peak/hole / e Å^{-3}	0.37/-0.37

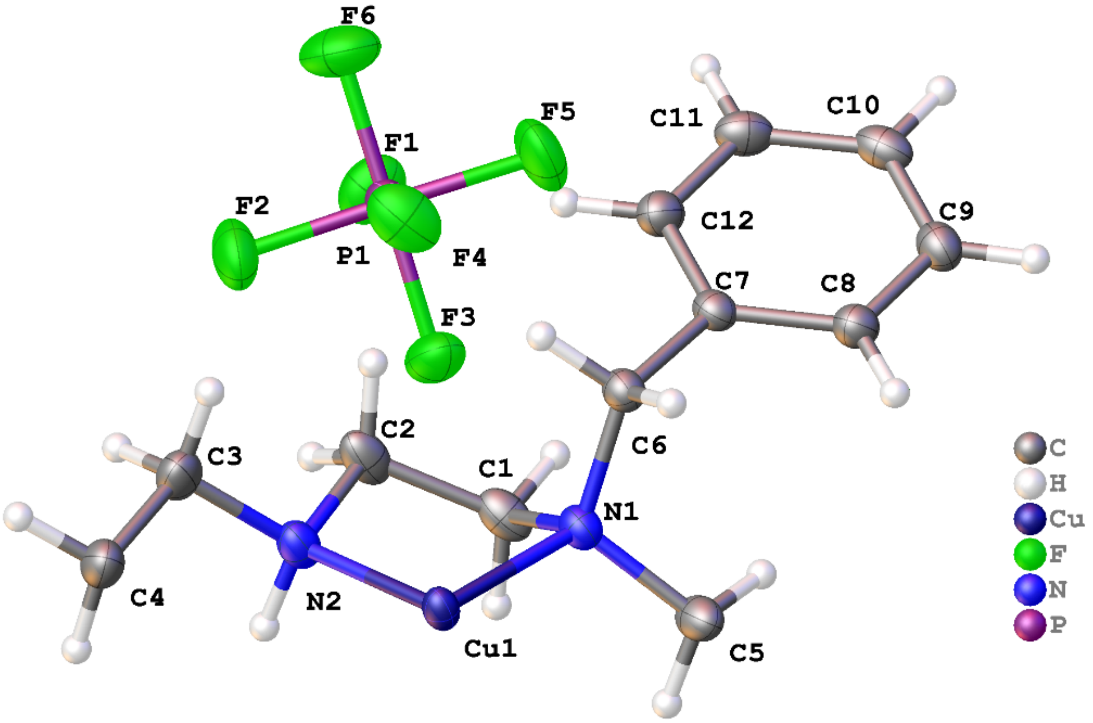

Figure S7: Asymmetric unit of [Cu(**2**)](PF_6)$_2$ **with atoms shown as 50% probability ellipsoids.**

Table S50 Fractional Atomic Coordinates (×10[4]) and Equivalent Isotropic Displacement Parameters (Å[2]×10[3]) for LE07 P1. U_{eq} is defined as 1/3 of the trace of the orthogonalised U_{IJ} tensor.

Atom	*x*	*y*	*z*	U(eq)
Cu1	5000	5000	5000	18.40(7)
N1	6480.8(16)	6769.2(14)	7070.5(13)	21.1(2)
N2	6203.6(17)	6494.3(14)	4198.2(14)	24.2(3)
C1	7960(2)	7831.8(18)	6922.1(17)	27.2(3)
C2	7224(2)	8073.4(17)	5395.5(18)	28.9(3)
C3	5021(2)	6592.2(19)	2712.2(18)	32.0(3)
C4	4039(2)	5024.1(19)	1431.4(17)	30.3(3)
C5	7304(2)	6210.9(19)	8372.4(16)	27.0(3)
C6	5317.3(19)	7583.5(16)	7295.5(15)	21.0(3)
C7	6278.3(19)	9036.3(16)	8693.5(15)	20.6(3)
C8	6487.2(19)	8941.9(17)	10111.9(16)	23.4(3)
C9	7261(2)	10280.9(18)	11364.1(16)	26.0(3)
C10	7835(2)	11751.3(18)	11224.0(17)	27.2(3)
C11	7687(2)	11866.3(18)	9839.9(19)	28.0(3)
C12	6905(2)	10517.6(17)	8581.4(17)	25.3(3)
P1	466.4(5)	6803.1(4)	3495.0(4)	25.16(10)
F1	2411.3(14)	8146.6(12)	4111.5(13)	43.0(3)
F2	333.1(15)	6196.6(14)	1868.8(11)	44.4(3)
F3	1419.7(15)	5637.5(12)	4036.2(11)	38.8(2)
F4	-1473.0(14)	5454.2(13)	2887.1(14)	45.5(3)
F5	597.4(18)	7377.4(14)	5119.7(12)	49.4(3)
F6	-460.1(17)	7959.1(14)	2957.1(15)	52.2(3)

Table S51 Anisotropic Displacement Parameters (Å[2]×10[3]) for LE07 P1. The Anisotropic displacement factor exponent takes the form: $-2\pi^2[h^2a^{*2}U_{11}+2hka^*b^*U_{12}+\ldots]$.

Atom	U_{11}	U_{22}	U_{33}	U_{23}	U_{13}	U_{12}
Cu1	22.87(13)	15.28(12)	16.44(11)	3.93(8)	11.02(9)	5.21(9)
N1	21.4(6)	21.7(6)	19.5(5)	4.3(4)	10.1(5)	8.4(5)
N2	31.3(7)	19.3(6)	24.3(6)	6.2(5)	17.3(5)	8.1(5)
C1	22.3(7)	24.0(7)	29.8(7)	0.2(6)	14.3(6)	4.5(6)
C2	35.7(8)	18.6(7)	33.3(8)	4.0(6)	23.6(7)	4.2(6)
C3	49.3(10)	27.7(8)	29.2(7)	15.6(6)	23.1(7)	18.8(7)
C4	43.5(9)	30.6(8)	22.6(7)	11.9(6)	16.7(7)	19.6(7)
C5	28.6(8)	30.5(8)	20.5(6)	5.9(6)	8.4(6)	16.6(6)
C6	21.8(7)	21.9(7)	19.2(6)	5.7(5)	9.8(5)	9.4(5)
C7	20.7(6)	21.6(7)	20.9(6)	5.7(5)	10.7(5)	10.2(5)
C8	26.0(7)	22.1(7)	24.4(7)	7.9(5)	13.4(6)	10.7(6)
C9	26.9(7)	29.6(8)	21.6(6)	5.8(6)	11.9(6)	12.6(6)
C10	22.2(7)	23.8(7)	29.0(7)	0.7(6)	9.4(6)	9.8(6)
C11	27.2(8)	20.5(7)	36.8(8)	9.1(6)	15.9(7)	9.6(6)
C12	28.5(8)	25.0(7)	27.5(7)	10.8(6)	15.8(6)	12.7(6)
P1	30.8(2)	22.24(19)	23.31(18)	6.57(15)	14.66(16)	9.80(16)
F1	37.6(6)	34.3(6)	46.3(6)	17.6(5)	15.5(5)	4.0(4)

Table S51 Anisotropic Displacement Parameters (Å^2×10^3) for LE07 P1. The Anisotropic displacement factor exponent takes the form: $-2\pi^2[h^2a^{*2}U_{11}+2hka^*b^*U_{12}+\ldots]$.

Atom	U_{11}	U_{22}	U_{33}	U_{23}	U_{13}	U_{12}
F2	51.3(7)	62.0(7)	24.9(5)	13.6(5)	20.9(5)	25.8(6)
F3	51.2(6)	29.1(5)	35.9(5)	13.0(4)	17.2(5)	20.2(5)
F4	36.2(6)	34.8(6)	54.7(7)	2.0(5)	24.7(5)	3.4(5)
F5	72.2(8)	43.1(6)	33.7(5)	2.4(5)	33.4(6)	18.3(6)
F6	54.5(7)	40.0(6)	60.8(7)	19.4(6)	18.7(6)	29.2(6)

Table S52 Bond Lengths for LE07 P1.

Atom	Atom	Length/Å	Atom	Atom	Length/Å
Cu1	N1	2.0496(12)	C7	C8	1.3997(19)
Cu1	N1[1]	2.0496(12)	C7	C12	1.393(2)
Cu1	N2	2.0129(12)	C8	C9	1.382(2)
Cu1	N2[1]	2.0129(12)	C9	C10	1.389(2)
N1	C1	1.4894(19)	C10	C11	1.386(2)
N1	C5	1.4976(18)	C11	C12	1.391(2)
N1	C6	1.5093(18)	P1	F1	1.6054(11)
N2	C2	1.4875(19)	P1	F2	1.5928(10)
N2	C3	1.487(2)	P1	F3	1.6076(10)
C1	C2	1.508(2)	P1	F4	1.6058(11)
C3	C4	1.517(2)	P1	F5	1.5880(10)
C4	C5[1]	1.518(2)	P1	F6	1.5850(11)
C6	C7	1.5109(19)			

[1]1-X,1-Y,1-Z

Table S53 Bond Angles for LE07 P1.

Atom	Atom	Atom	Angle/°	Atom	Atom	Atom	Angle/°
N1[1]	Cu1	N1	180.00(10)	C12	C7	C6	120.36(12)
N2[1]	Cu1	N1[1]	86.58(5)	C12	C7	C8	118.24(13)
N2[1]	Cu1	N1	93.42(5)	C9	C8	C7	121.10(14)
N2	Cu1	N1	86.58(5)	C8	C9	C10	119.99(14)
N2	Cu1	N1[1]	93.42(5)	C11	C10	C9	119.68(14)
N2[1]	Cu1	N2	180.00(5)	C10	C11	C12	120.18(14)
C1	N1	Cu1	103.57(8)	C11	C12	C7	120.76(14)
C1	N1	C5	108.03(12)	F1	P1	F3	89.01(6)
C1	N1	C6	111.30(11)	F1	P1	F4	179.61(7)
C5	N1	Cu1	112.93(9)	F2	P1	F1	90.44(6)
C5	N1	C6	111.77(11)	F2	P1	F3	89.09(6)
C6	N1	Cu1	108.97(8)	F2	P1	F4	89.75(6)
C2	N2	Cu1	108.37(9)	F4	P1	F3	90.64(6)
C3	N2	Cu1	116.41(10)	F5	P1	F1	90.05(7)
C3	N2	C2	110.68(12)	F5	P1	F2	178.95(7)
N1	C1	C2	110.17(12)	F5	P1	F3	89.99(6)

Table S53 Bond Angles for LE07 P1.

Atom	Atom	Atom	Angle/°	Atom	Atom	Atom	Angle/°
N2	C2	C1	107.39(12)	F5	P1	F4	89.75(6)
N2	C3	C4	112.56(12)	F6	P1	F1	90.56(6)
C3	C4	C5[1]	114.03(13)	F6	P1	F2	90.61(7)
N1	C5	C4[1]	114.52(12)	F6	P1	F3	179.48(7)
N1	C6	C7	115.24(11)	F6	P1	F4	89.78(7)
C8	C7	C6	121.33(13)	F6	P1	F5	90.32(7)

[1]1-X,1-Y,1-Z

Table S54 Torsion Angles for LE07 P1.

A	B	C	D	Angle/°	A	B	C	D	Angle/°
Cu1	N1	C1	C2	44.51(13)	C5	N1	C1	C2	164.51(12)
Cu1	N1	C5	C4[1]	-59.90(14)	C5	N1	C6	C7	61.12(15)
Cu1	N1	C6	C7	-173.37(9)	C6	N1	C1	C2	-72.43(14)
Cu1	N2	C2	C1	35.29(14)	C6	N1	C5	C4[1]	63.40(15)
Cu1	N2	C3	C4	-59.27(16)	C6	C7	C8	C9	-175.65(13)
N1	C1	C2	N2	-54.73(15)	C6	C7	C12	C11	175.80(13)
N1	C6	C7	C8	-87.43(16)	C7	C8	C9	C10	0.5(2)
N1	C6	C7	C12	95.69(16)	C8	C7	C12	C11	-1.2(2)
N2	C3	C4	C5[1]	65.56(18)	C8	C9	C10	C11	-2.3(2)
C1	N1	C5	C4[1]	-173.83(12)	C9	C10	C11	C12	2.5(2)
C1	N1	C6	C7	-59.77(15)	C10	C11	C12	C7	-0.7(2)
C2	N2	C3	C4	176.42(13)	C12	C7	C8	C9	1.3(2)
C3	N2	C2	C1	164.07(12)					

[1]1-X,1-Y,1-Z

Table S55 Hydrogen Atom Coordinates (Å×10^4) and Isotropic Displacement Parameters (Å^2×10^3) for LE07 P1.

Atom	*x*	*y*	*z*	U(eq)
H2	7103.27	6133.61	4056.83	29
H1A	8857.3	7368.58	7018.92	33
H1B	8565.95	8856.57	7751.52	33
H2A	6424.77	8640.63	5332.03	35
H2B	8216.75	8699.54	5256.59	35
H3A	5756.21	7365.34	2441.5	38
H3B	4130.46	6973.75	2819.76	38
H4A	4933.51	4622.67	1363.11	36
H4B	3399.8	5189.46	454.8	36
H5A	8022.3	7124.81	9327.91	32
H5B	8135.3	5773.54	8217.22	32
H6A	4337.07	6827.1	7359.36	25

Table S55 Hydrogen Atom Coordinates (Å×10^4) and Isotropic Displacement Parameters (Å^2×10^3) for LE07 P1.

Atom	*x*	*y*	*z*	U(eq)
H6B	4759.33	7878.46	6382.97	25
H8	6090.52	7940.88	10215.77	28
H9	7400.39	10195.38	12320.26	31
H10	8326.23	12673.81	12072.83	33
H11	8120.52	12869.22	9750.78	34
H12	6797.23	10607.69	7634.09	30

Table S56 Crystal data and structure refinement for [Ni(**2'**)(OAc)](PF_6).MeCN**. [MONO]**

Identification code	mono
Empirical formula	$C_{28}H_{43}F_6N_4NiO_3P$
Formula weight	687.34
Temperature/K	150.15
Crystal system	monoclinic
Space group	$P2_1/n$
a/Å	10.145(2)
b/Å	27.296(6)
c/Å	12.454(3)
α/°	90
β/°	101.37(3)
γ/°	90
Volume/Å^3	3381.1(12)
Z	4
ρ_{calc}g/cm^3	1.350
μ/mm^{-1}	0.864
F(000)	1440.0
Crystal size/mm^3	0.05 × 0.03 × 0.025
Radiation	synchrotron (λ = 0.7749 Å)
2Θ range for data collection/°	4.752 to 58.05
Index ranges	$-12 \le h \le 12$, $-34 \le k \le 34$, $-15 \le l \le 15$
Reflections collected	54095
Independent reflections	6931 [R_{int} = 0.0776, R_{sigma} = 0.0585]
Data/restraints/parameters	6931/0/391
Goodness-of-fit on F^2	1.023
Final R indexes [I>=2σ (I)]	R_1 = 0.0526, wR_2 = 0.1047
Final R indexes [all data]	R_1 = 0.0810, wR_2 = 0.1144
Largest diff. peak/hole / e Å^{-3}	0.79/-0.54

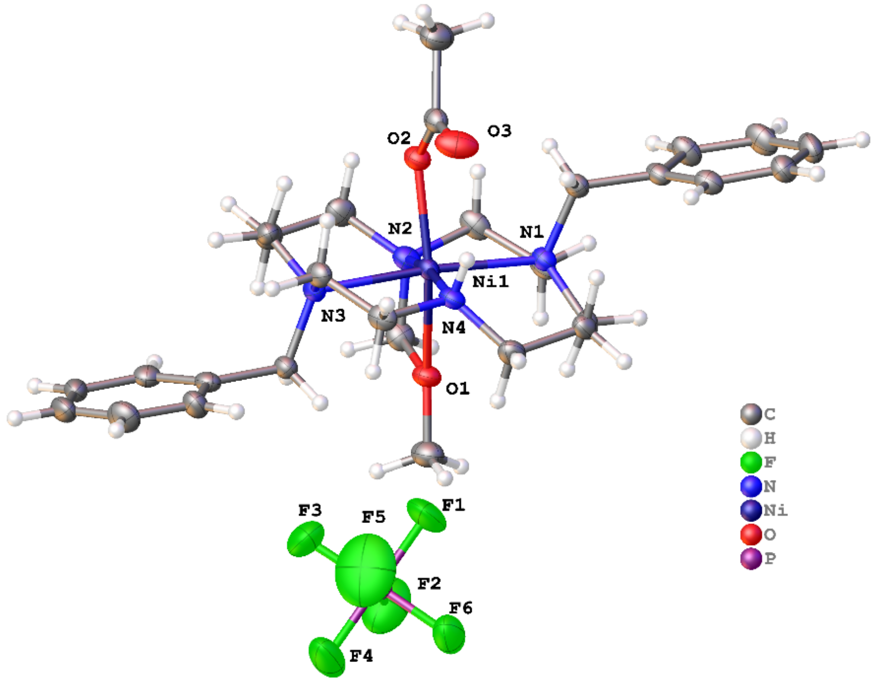

Figure S8: Asymmetric unit of [Ni(**2'**)(OAc)](PF_6).MeCN **atoms shown as 50% probability ellipsoids.**

Table S57 Fractional Atomic Coordinates (×10[4]) and Equivalent Isotropic Displacement Parameters (Å[2]×10[3]) for MONO. U_{eq} is defined as 1/3 of of the trace of the orthogonalised U_{IJ} tensor.

Atom	*x*	*y*	*z*	U(eq)
Ni1	2913.2(3)	6304.5(2)	5190.9(3)	17.61(11)
P1	2309.3(8)	6996.6(3)	382.2(7)	31.1(2)
O2	2553.2(19)	5770.5(7)	6236.0(17)	24.2(5)
O1	3060(2)	7107.3(7)	4648.9(17)	25.3(5)
O3	3171(3)	5097.8(8)	5456.2(19)	40.0(6)
N1	5003(2)	6366.4(9)	5974(2)	20.8(5)
F6	3654(2)	7067.8(10)	-86.1(19)	61.2(7)
N4	3303(2)	5866.4(10)	3973(2)	21.7(6)
N2	2578(2)	6851.4(9)	6269(2)	24.5(6)
N3	852(2)	6290.2(9)	4298(2)	21.8(5)
F3	966(2)	6950.4(10)	855(2)	69.8(8)
F1	3114(2)	6742.2(12)	1430(2)	83.3(9)
F4	1486(2)	7276.8(13)	-653(2)	87.8(10)
F2	2549(3)	7510.4(10)	967(3)	84.4(9)
C12	6965(3)	5910.1(11)	7215(2)	23.0(7)
C10	5838(3)	6439.7(11)	5124(3)	24.1(7)
C27	2802(3)	5319.4(11)	6209(3)	23.4(7)
C9	5750(3)	6028.5(12)	4292(2)	25.4(7)
C5	-40(3)	6273.6(12)	5112(3)	26.3(7)
C19	-966(3)	6642.6(11)	2783(3)	24.6(7)
C11	5487(3)	5928.7(11)	6687(2)	23.0(7)
C2	3795(3)	6841.4(12)	7160(3)	27.8(7)
C1	5038(3)	6815.3(11)	6658(3)	26.5(7)
C8	4444(3)	5999.8(12)	3461(2)	26.9(7)
C6	867(3)	5809.0(11)	3746(3)	25.5(7)
C14	9185(3)	5579.4(12)	7252(3)	29.9(8)
C17	7481(3)	6154.1(12)	8190(3)	29.8(8)
C20	-2038(3)	6890.2(11)	3075(3)	27.3(7)
C13	7839(3)	5619.8(11)	6760(3)	25.2(7)
C24	-1209(3)	6368.5(13)	1829(3)	31.0(8)
C7	2043(3)	5777.5(11)	3165(2)	25.3(7)
C18	444(3)	6690.9(11)	3470(2)	23.1(7)
C25	2573(3)	7284.6(12)	5582(3)	28.1(7)
C21	-3326(3)	6850.1(13)	2444(3)	31.3(8)
C16	8832(3)	6122.9(13)	8666(3)	34.6(8)
C3	1333(3)	6779.5(13)	6717(3)	29.8(8)
C22	-3568(3)	6564.4(14)	1524(3)	36.7(9)
C4	44(3)	6717.3(12)	5862(3)	29.7(8)
C15	9689(3)	5837.0(13)	8208(3)	33.7(8)
C26	3181(3)	7482.8(13)	3882(3)	35.3(8)
F5	2020(4)	6514.1(12)	-247(3)	122.5(14)
C23	-2503(3)	6320.8(14)	1213(3)	38.5(8)
C28	2718(4)	5029.2(13)	7239(3)	39.7(9)

Table S58 Anisotropic Displacement Parameters (Å^2×10^3) for MONO. The Anisotropic displacement factor exponent takes the form: $-2\pi^2[h^2a^{*2}U_{11}+2hka^*b^*U_{12}+\ldots]$.

Atom	U_{11}	U_{22}	U_{33}	U_{23}	U_{13}	U_{12}
Ni1	17.97(18)	17.8(2)	15.6(2)	-0.28(17)	-0.14(14)	0.50(15)
P1	30.7(4)	36.2(5)	23.8(5)	4.4(4)	-1.0(4)	-2.7(4)
O2	26.1(11)	21.5(12)	23.8(12)	2.8(9)	1.6(9)	-0.2(9)
O1	29.8(11)	23.0(12)	22.2(12)	5.6(10)	2.7(9)	-0.1(9)
O3	68.2(17)	22.3(13)	29.4(14)	0.0(11)	9.5(12)	1.3(12)
N1	21.9(12)	18.2(14)	20.7(13)	0.8(11)	0.0(10)	-0.1(10)
F6	38.8(12)	94(2)	52.0(15)	18.2(14)	12.1(11)	16.2(12)
N4	25.1(13)	18.7(13)	19.3(14)	4.2(11)	-0.2(10)	0.7(10)
N2	28.8(13)	20.7(14)	23.1(15)	1.6(11)	2.9(11)	2.9(11)
N3	18.4(12)	23.9(14)	21.3(14)	3.5(12)	-0.3(10)	-1.3(10)
F3	45.8(13)	99(2)	68.8(17)	29.1(15)	20.7(12)	-11.7(13)
F1	58.4(15)	126(3)	61.2(17)	56.6(17)	1.1(13)	23.8(16)
F4	51.1(15)	159(3)	53.5(16)	47.5(18)	9.9(12)	32.3(17)
F2	81.6(19)	53.8(17)	124(3)	-24.2(17)	35.0(18)	-12.6(14)
C12	24.0(15)	20.2(16)	22.2(17)	5.3(13)	-2.0(13)	-1.4(12)
C10	17.3(14)	23.1(17)	30.7(18)	6.5(14)	1.8(13)	0.4(12)
C27	20.6(15)	22.7(18)	24.9(18)	2.2(14)	-0.7(12)	-2.8(12)
C9	23.7(15)	29.3(18)	23.4(17)	5.3(14)	5.3(13)	6.4(13)
C5	19.3(14)	33.1(18)	26.0(17)	9.5(15)	2.8(12)	-1.0(13)
C19	25.2(15)	25.9(18)	21.9(17)	9.4(14)	2.4(13)	-1.3(13)
C11	22.2(15)	25.3(17)	19.9(17)	2.3(13)	0.2(12)	-0.9(13)
C2	35.7(17)	24.5(18)	19.6(17)	-6.6(14)	-3.3(14)	2.9(14)
C1	27.1(16)	19.4(17)	27.6(18)	-0.1(14)	-7.8(13)	-1.0(13)
C8	28.5(16)	32.8(19)	19.7(17)	2.4(14)	5.3(13)	3.6(14)
C6	23.1(15)	28.5(18)	22.1(17)	1.8(14)	-2.3(13)	-5.5(13)
C14	26.0(16)	31.3(19)	31.5(19)	5.9(15)	3.4(14)	4.5(14)
C17	33.2(17)	30.9(19)	22.2(18)	-2.2(14)	-2.4(14)	5.6(14)
C20	28.1(16)	24.1(18)	29.7(19)	10.2(15)	6.0(14)	0.8(13)
C13	30.2(16)	22.1(17)	21.2(17)	1.5(13)	0.1(13)	-0.1(13)
C24	24.9(16)	41(2)	25.5(18)	6.9(16)	0.1(13)	3.4(14)
C7	30.5(16)	21.8(17)	21.2(17)	-3.0(14)	-0.4(13)	-4.0(13)
C18	20.3(14)	25.4(17)	22.1(17)	5.2(13)	0.9(12)	-0.9(12)
C25	30.2(17)	24.1(18)	29.2(19)	-3.1(15)	3.7(14)	2.7(13)
C21	20.0(15)	38(2)	36(2)	17.9(17)	4.0(14)	2.3(14)
C16	38.5(19)	33(2)	26.5(19)	-2.6(16)	-9.2(15)	-1.6(15)
C3	33.9(17)	32.9(19)	24.2(18)	-1.6(15)	9.4(14)	5.3(14)
C22	24.8(17)	50(2)	31(2)	17.8(18)	-4.6(14)	-3.7(16)
C4	27.0(16)	36(2)	28.1(19)	5.0(15)	9.8(14)	5.3(14)
C15	23.8(16)	36(2)	36(2)	7.4(16)	-6.0(15)	-0.4(14)
C26	38.2(19)	34(2)	32(2)	8.5(16)	2.4(16)	-4.2(15)
F5	126(3)	78(2)	157(3)	-66(2)	12(3)	-33(2)
C23	38.6(19)	48(2)	25.5(19)	1.6(18)	-3.2(15)	-3.9(17)
C28	43(2)	36(2)	42(2)	12.0(18)	11.9(17)	6.6(17)

Table S59 Bond Lengths for MONO.

Atom	Atom	Length/Å	Atom	Atom	Length/Å
Ni1	O2	2.035(2)	N3	C5	1.487(4)
Ni1	O1	2.306(2)	N3	C6	1.484(4)
Ni1	N1	2.159(2)	N3	C18	1.504(4)
Ni1	N4	2.031(3)	C12	C11	1.515(4)
Ni1	N2	2.080(3)	C12	C17	1.394(4)
Ni1	N3	2.167(2)	C12	C13	1.390(4)
P1	F6	1.597(2)	C10	C9	1.518(4)
P1	F3	1.593(2)	C27	C28	1.524(4)
P1	F1	1.559(2)	C9	C8	1.513(4)
P1	F4	1.587(2)	C5	C4	1.521(5)
P1	F2	1.577(3)	C19	C20	1.388(4)
P1	F5	1.531(3)	C19	C24	1.385(5)
O2	C27	1.259(4)	C19	C18	1.521(4)
O1	C25	1.434(4)	C2	C1	1.516(4)
O1	C26	1.422(4)	C6	C7	1.514(4)
O3	C27	1.234(4)	C14	C13	1.386(4)
N1	C10	1.494(4)	C14	C15	1.390(5)
N1	C11	1.512(4)	C17	C16	1.384(4)
N1	C1	1.489(4)	C20	C21	1.390(4)
N4	C8	1.474(4)	C24	C23	1.390(4)
N4	C7	1.483(4)	C21	C22	1.368(5)
N2	C2	1.489(4)	C16	C15	1.373(5)
N2	C25	1.459(4)	C3	C4	1.525(4)
N2	C3	1.490(4)	C22	C23	1.387(5)

Table S60 Bond Angles for MONO.

Atom	Atom	Atom	Angle/˚	Atom	Atom	Atom	Angle/˚
O2	Ni1	O1	153.90(8)	C2	N2	C3	111.0(2)
O2	Ni1	N1	92.94(9)	C25	N2	Ni1	100.69(18)
O2	Ni1	N2	91.64(9)	C25	N2	C2	111.0(2)
O2	Ni1	N3	92.22(9)	C25	N2	C3	114.9(2)
N1	Ni1	O1	86.76(8)	C3	N2	Ni1	113.91(19)
N1	Ni1	N3	174.83(9)	C5	N3	Ni1	107.81(17)
N4	Ni1	O2	98.15(10)	C5	N3	C18	111.6(2)
N4	Ni1	O1	107.92(9)	C6	N3	Ni1	99.01(16)
N4	Ni1	N1	93.73(10)	C6	N3	C5	110.6(2)
N4	Ni1	N2	170.21(10)	C6	N3	C18	110.6(2)
N4	Ni1	N3	85.19(10)	C18	N3	Ni1	116.48(17)
N2	Ni1	O1	62.30(9)	C17	C12	C11	122.1(3)
N2	Ni1	N1	85.60(10)	C13	C12	C11	119.9(3)
N2	Ni1	N3	94.60(10)	C13	C12	C17	117.8(3)
N3	Ni1	O1	88.76(8)	N1	C10	C9	114.8(2)
F3	P1	F6	177.55(16)	O2	C27	C28	116.2(3)
F1	P1	F6	90.93(13)	O3	C27	O2	126.3(3)

Table S60 Bond Angles for MONO.

Atom	Atom	Atom	Angle/°	Atom	Atom	Atom	Angle/°
F1	P1	F3	89.99(14)	O3	C27	C28	117.4(3)
F1	P1	F4	177.44(19)	C8	C9	C10	115.5(2)
F1	P1	F2	90.12(18)	N3	C5	C4	115.3(2)
F4	P1	F6	89.74(13)	C20	C19	C18	120.6(3)
F4	P1	F3	89.24(14)	C24	C19	C20	118.1(3)
F2	P1	F6	89.73(15)	C24	C19	C18	121.2(3)
F2	P1	F3	88.00(15)	N1	C11	C12	117.4(2)
F2	P1	F4	87.41(18)	N2	C2	C1	109.2(2)
F5	P1	F6	90.51(18)	N1	C1	C2	110.3(2)
F5	P1	F3	91.70(18)	N4	C8	C9	111.9(2)
F5	P1	F1	93.5(2)	N3	C6	C7	110.7(2)
F5	P1	F4	89.0(2)	C13	C14	C15	120.2(3)
F5	P1	F2	176.4(2)	C16	C17	C12	120.9(3)
C27	O2	Ni1	128.4(2)	C19	C20	C21	120.7(3)
C25	O1	Ni1	91.77(16)	C14	C13	C12	121.1(3)
C26	O1	Ni1	154.2(2)	C19	C24	C23	120.9(3)
C26	O1	C25	113.0(2)	N4	C7	C6	108.8(2)
C10	N1	Ni1	109.52(17)	N3	C18	C19	115.1(2)
C10	N1	C11	111.0(2)	O1	C25	N2	103.9(2)
C11	N1	Ni1	112.31(17)	C22	C21	C20	120.8(3)
C1	N1	Ni1	103.49(17)	C15	C16	C17	120.7(3)
C1	N1	C10	110.0(2)	N2	C3	C4	115.3(3)
C1	N1	C11	110.2(2)	C21	C22	C23	119.1(3)
C8	N4	Ni1	117.58(19)	C5	C4	C3	117.0(3)
C8	N4	C7	113.1(2)	C16	C15	C14	119.2(3)
C7	N4	Ni1	109.54(18)	C22	C23	C24	120.3(3)
C2	N2	Ni1	104.52(18)				

Table S61 Torsion Angles for MONO.

A	B	C	D	Angle/°	A	B	C	D	Angle/°
Ni1	O2	C27	O3	-9.7(4)	C2	N2	C25	O1	99.1(3)
Ni1	O2	C27	C28	166.4(2)	C2	N2	C3	C4	-172.4(3)
Ni1	O1	C25	N2	9.8(2)	C1	N1	C10	C9	-173.2(2)
Ni1	N1	C10	C9	-60.1(3)	C1	N1	C11	C12	-69.6(3)
Ni1	N1	C11	C12	175.5(2)	C8	N4	C7	C6	-161.6(3)
Ni1	N1	C1	C2	38.5(3)	C6	N3	C5	C4	170.2(2)
Ni1	N4	C8	C9	57.6(3)	C6	N3	C18	C19	62.5(3)
Ni1	N4	C7	C6	-28.3(3)	C17	C12	C11	N1	83.3(4)
Ni1	N2	C2	C1	45.8(3)	C17	C12	C13	C14	-0.9(5)
Ni1	N2	C25	O1	-11.1(2)	C17	C16	C15	C14	0.3(5)
Ni1	N2	C3	C4	-54.8(3)	C20	C19	C24	C23	-3.5(5)
Ni1	N3	C5	C4	63.0(3)	C20	C19	C18	N3	96.1(3)
Ni1	N3	C6	C7	-51.1(2)	C20	C21	C22	C23	-1.2(5)
Ni1	N3	C18	C19	174.5(2)	C13	C12	C11	N1	-100.5(3)

Table S61 Torsion Angles for MONO.

A	B	C	D	Angle/°	A	B	C	D	Angle/°
N1	C10	C9	C8	72.6(3)	C13	C12	C17	C16	2.5(5)
N2	C2	C1	N1	-59.6(3)	C13	C14	C15	C16	1.3(5)
N2	C3	C4	C5	62.7(4)	C24	C19	C20	C21	2.1(5)
N3	C5	C4	C3	-70.1(4)	C24	C19	C18	N3	-86.6(4)
N3	C6	C7	N4	56.7(3)	C7	N4	C8	C9	-173.1(3)
C12	C17	C16	C15	-2.2(5)	C18	N3	C5	C4	-66.1(3)
C10	N1	C11	C12	52.5(3)	C18	N3	C6	C7	71.7(3)
C10	N1	C1	C2	155.4(2)	C18	C19	C20	C21	179.5(3)
C10	C9	C8	N4	-67.0(4)	C18	C19	C24	C23	179.1(3)
C5	N3	C6	C7	-164.1(2)	C25	N2	C2	C1	-61.9(3)
C5	N3	C18	C19	-61.2(3)	C25	N2	C3	C4	60.6(4)
C19	C20	C21	C22	0.3(5)	C21	C22	C23	C24	-0.2(5)
C19	C24	C23	C22	2.6(5)	C3	N2	C2	C1	169.0(3)
C11	N1	C10	C9	64.5(3)	C3	N2	C25	O1	-133.9(2)
C11	N1	C1	C2	-81.8(3)	C15	C14	C13	C12	-1.0(5)
C11	C12	C17	C16	178.7(3)	C26	O1	C25	N2	-177.8(2)
C11	C12	C13	C14	-177.2(3)					

Table S62 Hydrogen Atom Coordinates (Å×10[4]) and Isotropic Displacement Parameters (Å[2]×10[3]) for MONO.

Atom	*x*	*y*	*z*	U(eq)
H4	3505(9)	5599(11)	4265(12)	26
H10A	6789.32	6477.83	5496.02	29
H10B	5557.61	6748.88	4728.67	29
H9A	6496.59	6066.82	3893.44	30
H9B	5885.25	5713.31	4692.05	30
H5A	184.87	5977.6	5570.66	32
H5B	-980.78	6238.59	4712.82	32
H11A	5270.84	5629.9	6235.95	28
H11B	4961.49	5914.04	7276.72	28
H2A	3761.03	6552.96	7635.63	33
H2B	3825.83	7140.16	7614.63	33
H1A	5082.57	7109.03	6199.99	32
H1B	5851.87	6810.64	7246.47	32
H8A	4262.87	6320.94	3091.87	32
H8B	4534.87	5753.17	2897.03	32
H6A	16.13	5764.7	3208.31	31
H6B	934.93	5543.35	4295.1	31
H14	9763.53	5375.23	6935.38	36
H17	6897.85	6344.46	8533.17	36
H20	-1889.11	7089.3	3712.95	33
H13	7508.98	5446.17	6101.06	30
H24	-481.74	6211.15	1591.22	37
H7A	2069.61	5449.2	2832.43	30
H7B	1942.44	6025.32	2574.46	30

Table S62 Hydrogen Atom Coordinates (Å×10^4) and Isotropic Displacement Parameters (Å^2×10^3) for MONO.

Atom	*x*	*y*	*z*	U(eq)
H18A	1093.25	6698.16	2971.4	28
H18B	510.5	7008.6	3860	28
H25A	1653.23	7419.53	5362.19	34
H25B	3171.74	7541.89	5969.75	34
H21	-4047.06	7023.21	2654.3	38
H16	9170.4	6301.19	9317.1	42
H3A	1223.94	7064.73	7182.16	36
H3B	1454.71	6486	7193.09	36
H22	-4452.98	6533.18	1103.53	44
H4A	-718.7	6701.43	6249.07	36
H4B	-81.7	7014.83	5398.2	36
H15	10614.16	5815.57	8539.09	40
H26A	3492.67	7340.17	3254.42	53
H26B	3829.78	7728.55	4232.74	53
H26C	2303.8	7638.43	3631.36	53
H23	-2659.32	6120.37	577.24	46
H28A	3606.57	5018.48	7721.03	60
H28B	2415.75	4694.88	7036.15	60
H28C	2077.2	5187.81	7622.83	60

Table S63 Solvent masks information for MONO.

Number	X	Y	Z	Volume	Electron count	Content
1	-0.890	0.000	0.500	255.1	44.3	2 MeCN
2	0.410	0.500	0.000	255.1	44.3	2 MeCN

Cyclic Voltammograms for Co, Ni and Cu Complexes of Ligands 1 and 2.

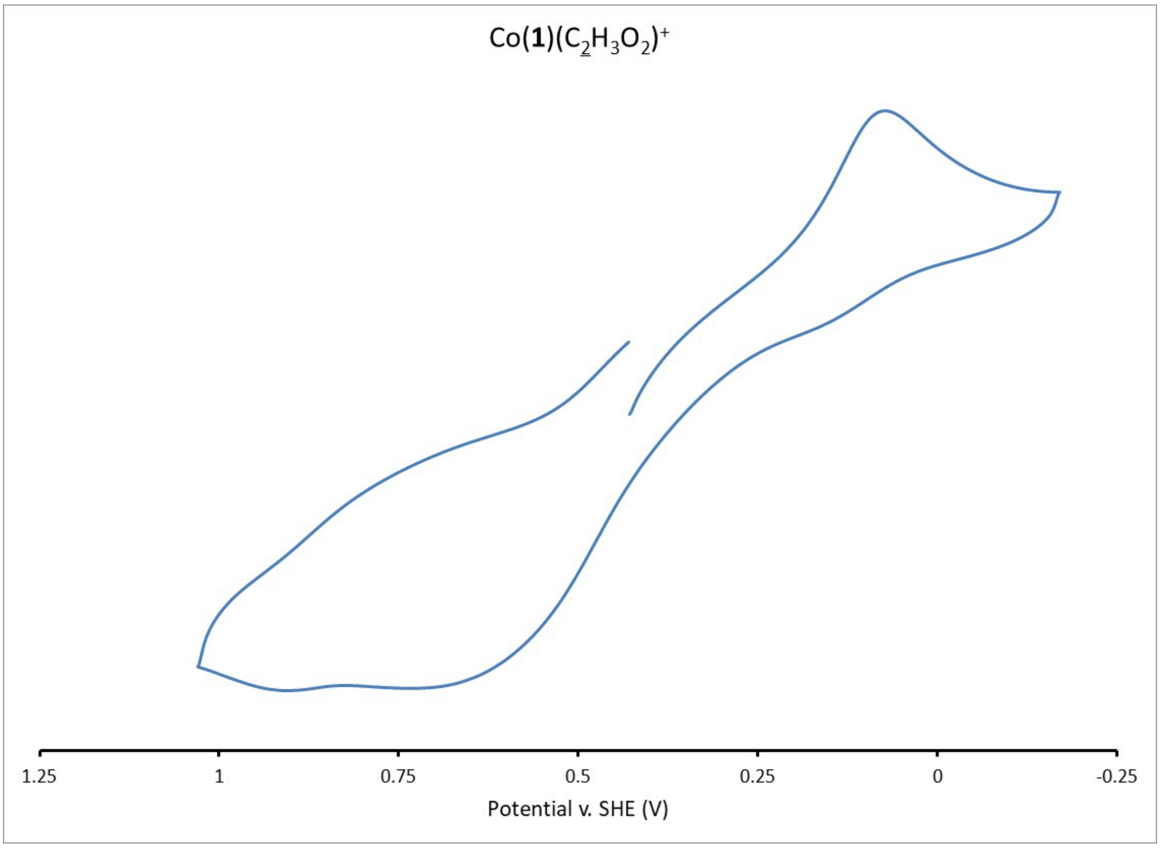

Figure S9. Cyclic Voltammogram of Co(1)($C_2H_3O_2$)$^+$ in Acetonitrile.

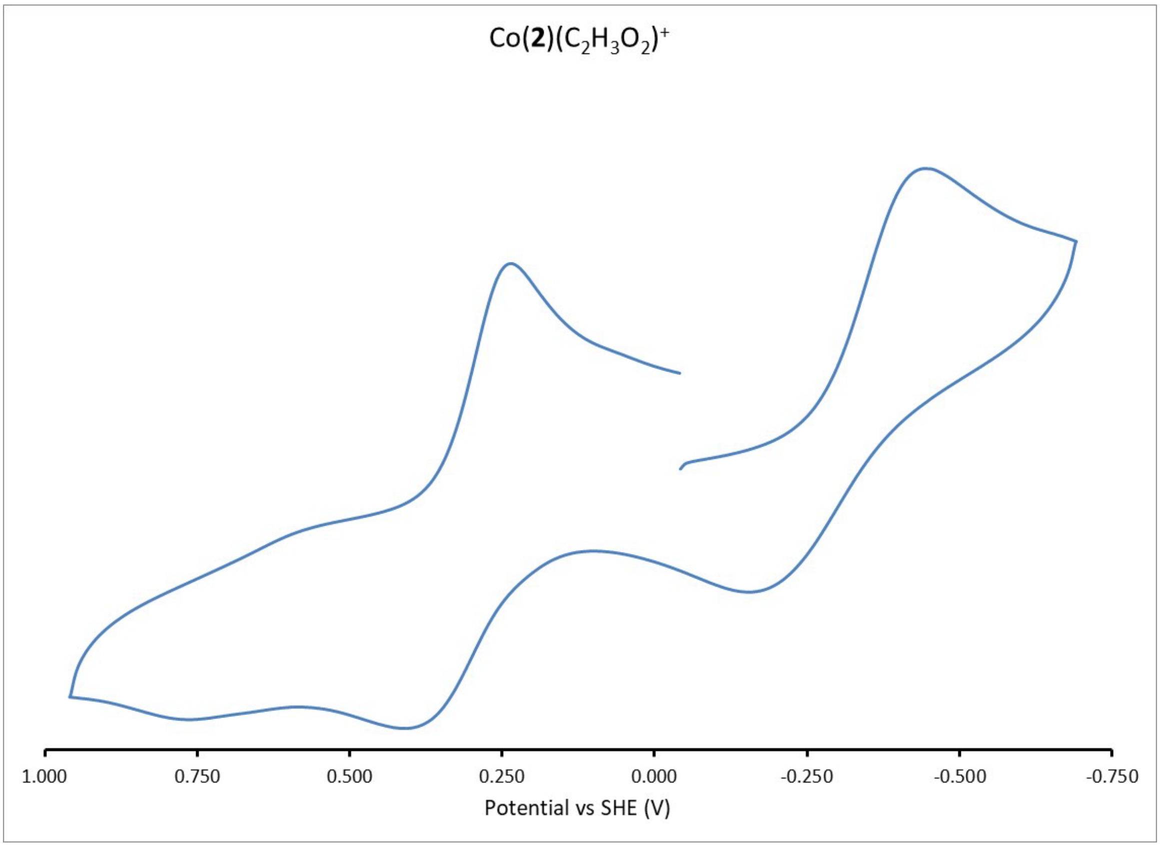

Figure S10. Cyclic Voltammogram of Co(2)($C_2H_3O_2$)$^+$ in Acetonitrile.

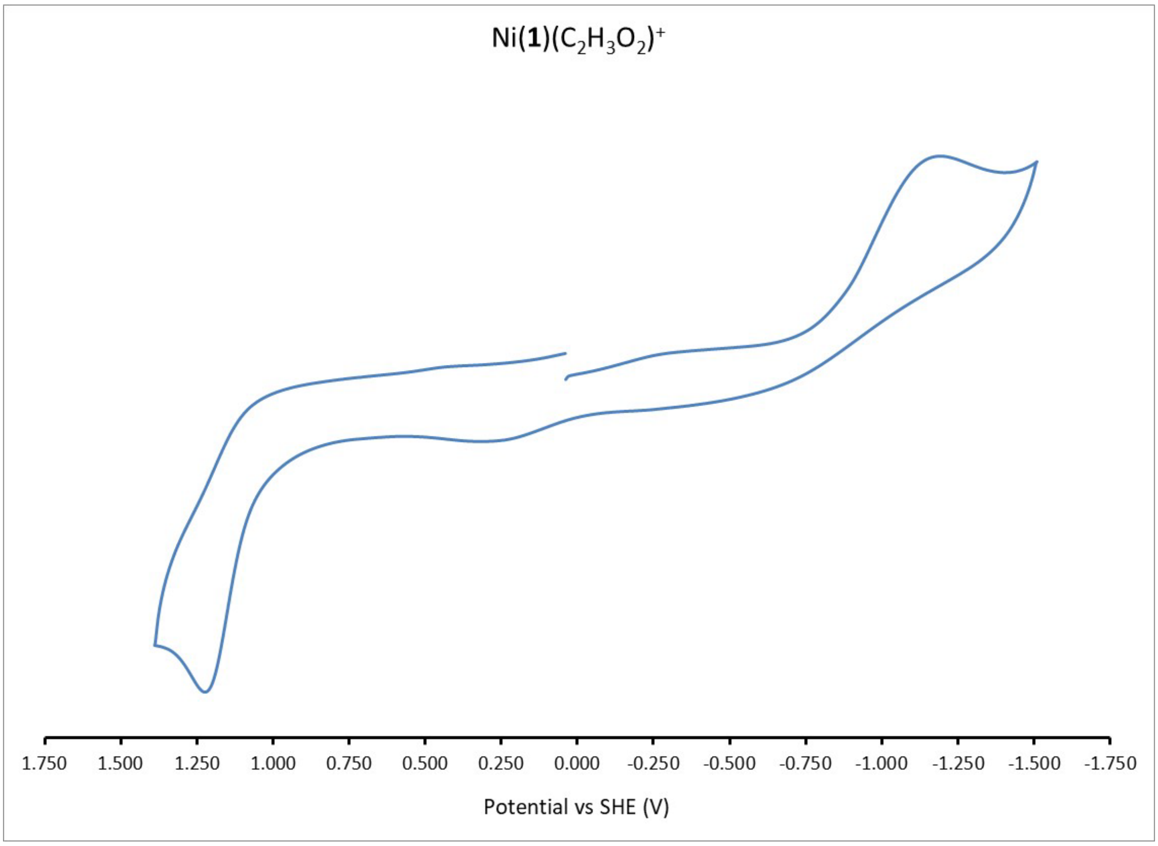

Figure S11. Cyclic Voltammogram of Ni(1)($C_2H_3O_2$)$^+$ in Acetonitrile.

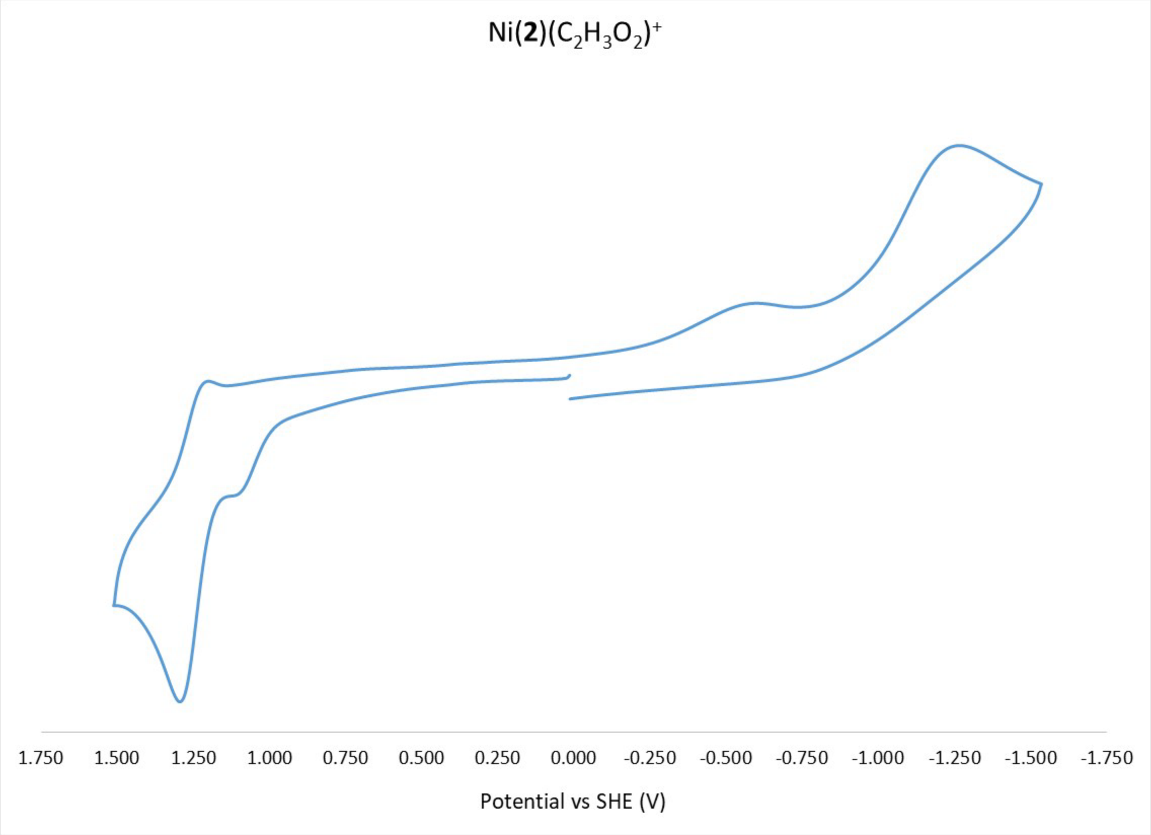

Figure S12. Cyclic Voltammogram of Ni(2)($C_2H_3O_2$)$^+$ in Acetonitrile.

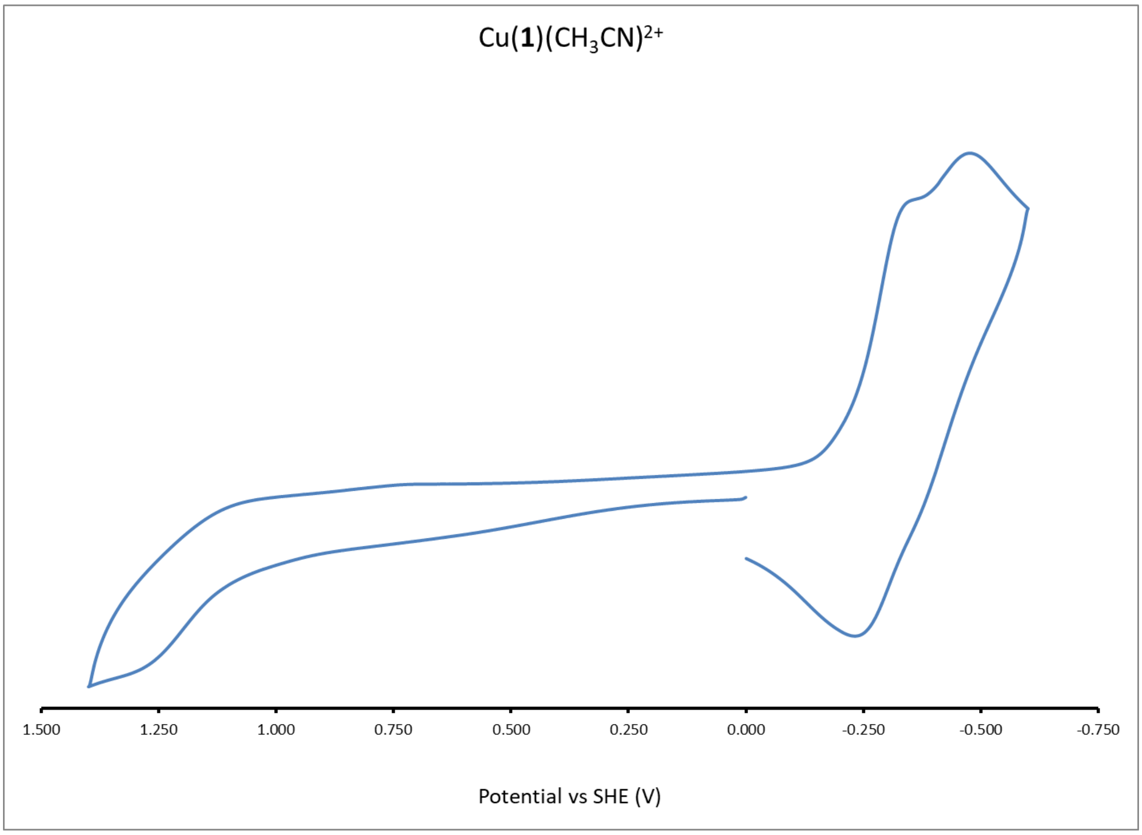

Figure S13. Cyclic Voltammogram of Cu(2)(CH_3CN)$^{2+}$ in Acetonitrile.

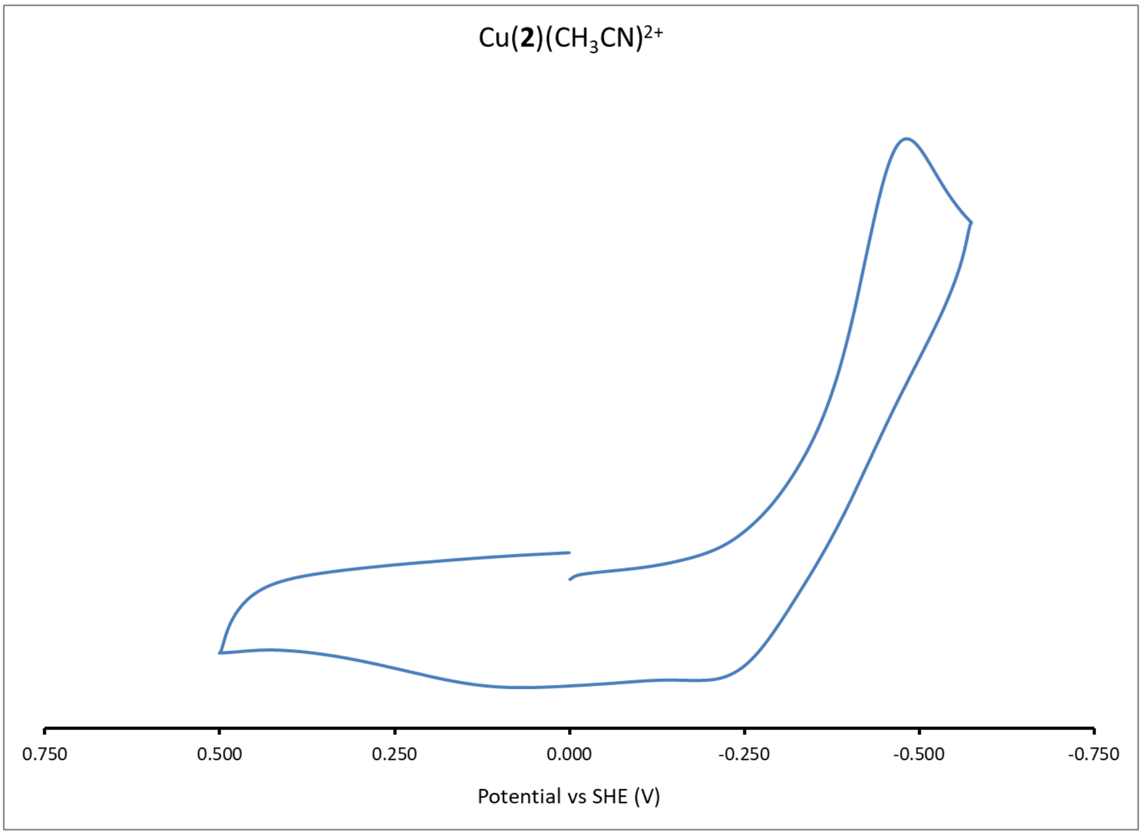

Figure S14. Cyclic Voltammogram of Cu(2)(CH_3CN)$^{2+}$ in Acetonitrile.

UV-Vis Spectra of Co, Ni, and Cu complexes of Ligands 1 and 2. Figures S15-S20.

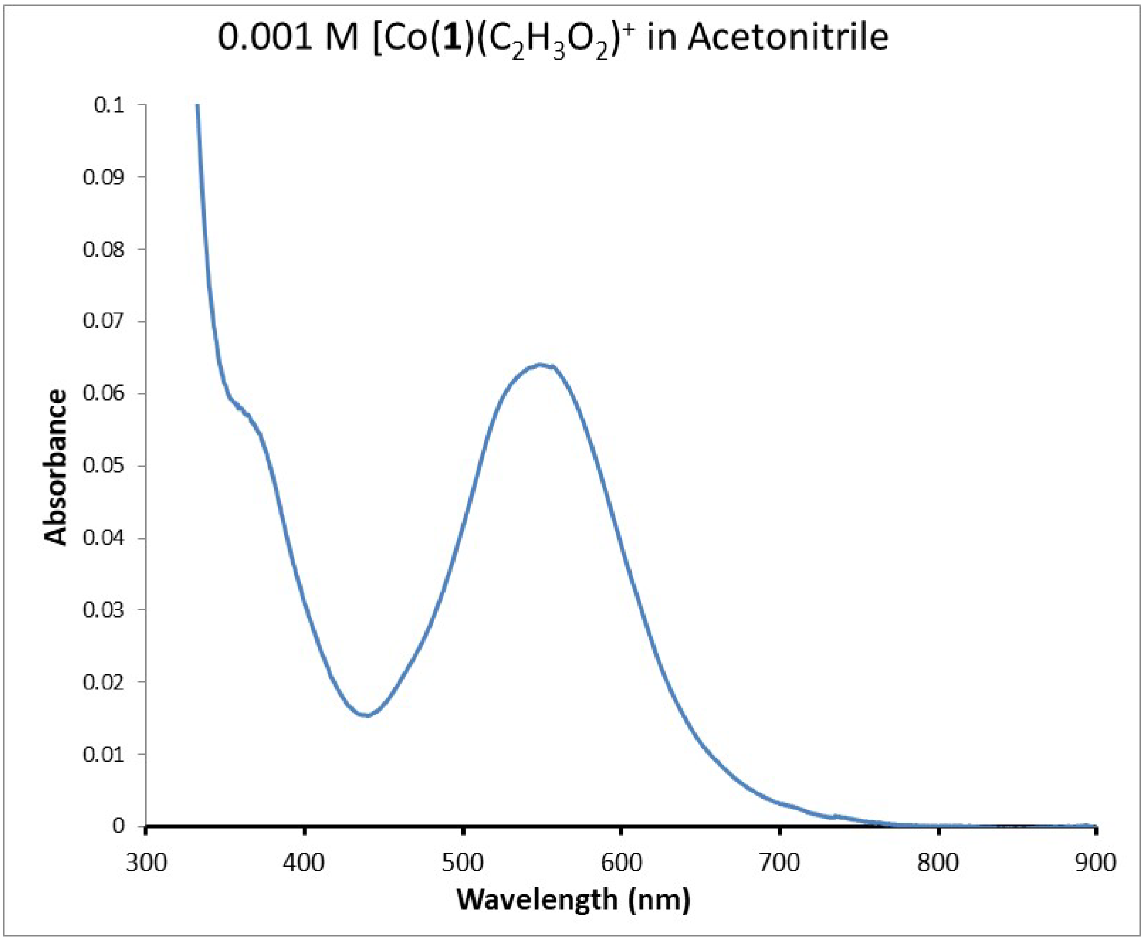

Figure S15. UV-Vis Spectrum of Co(1)($C_2H_3O_2$)$^+$ in Acetonitrile.

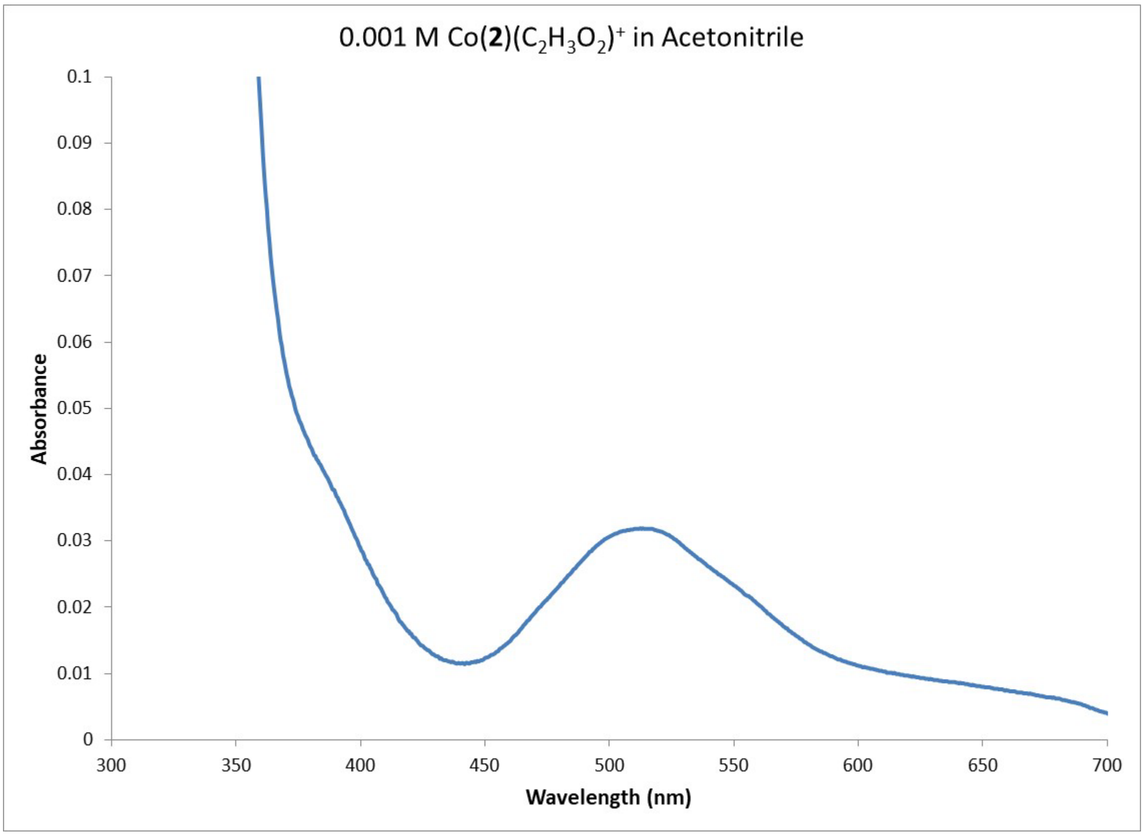

Figure S16. UV-Vis Spectrum of Co(2)($C_2H_3O_2$)$^+$ in Acetonitrile.

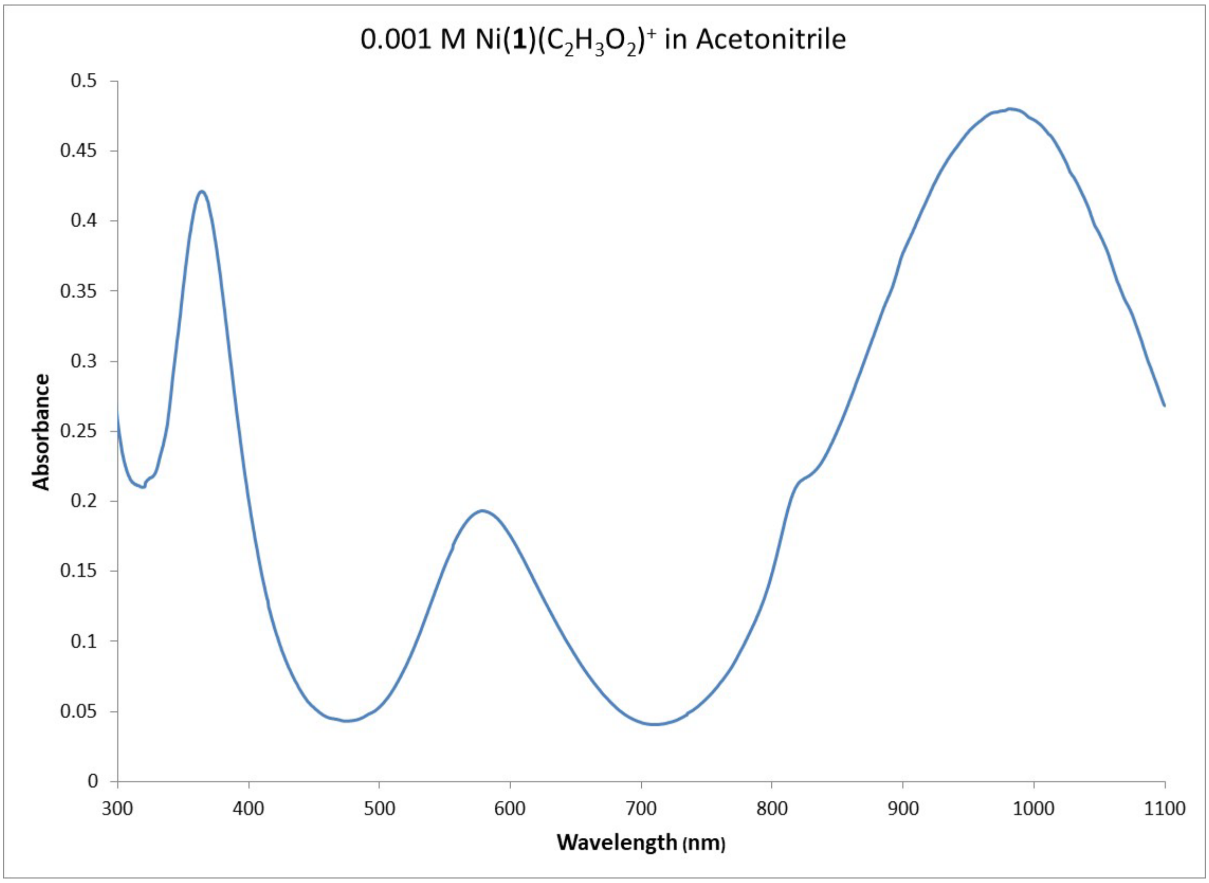

Figure S17. UV-Vis Spectrum of Ni(1)($C_2H_3O_2$)$^+$ in Acetonitrile.

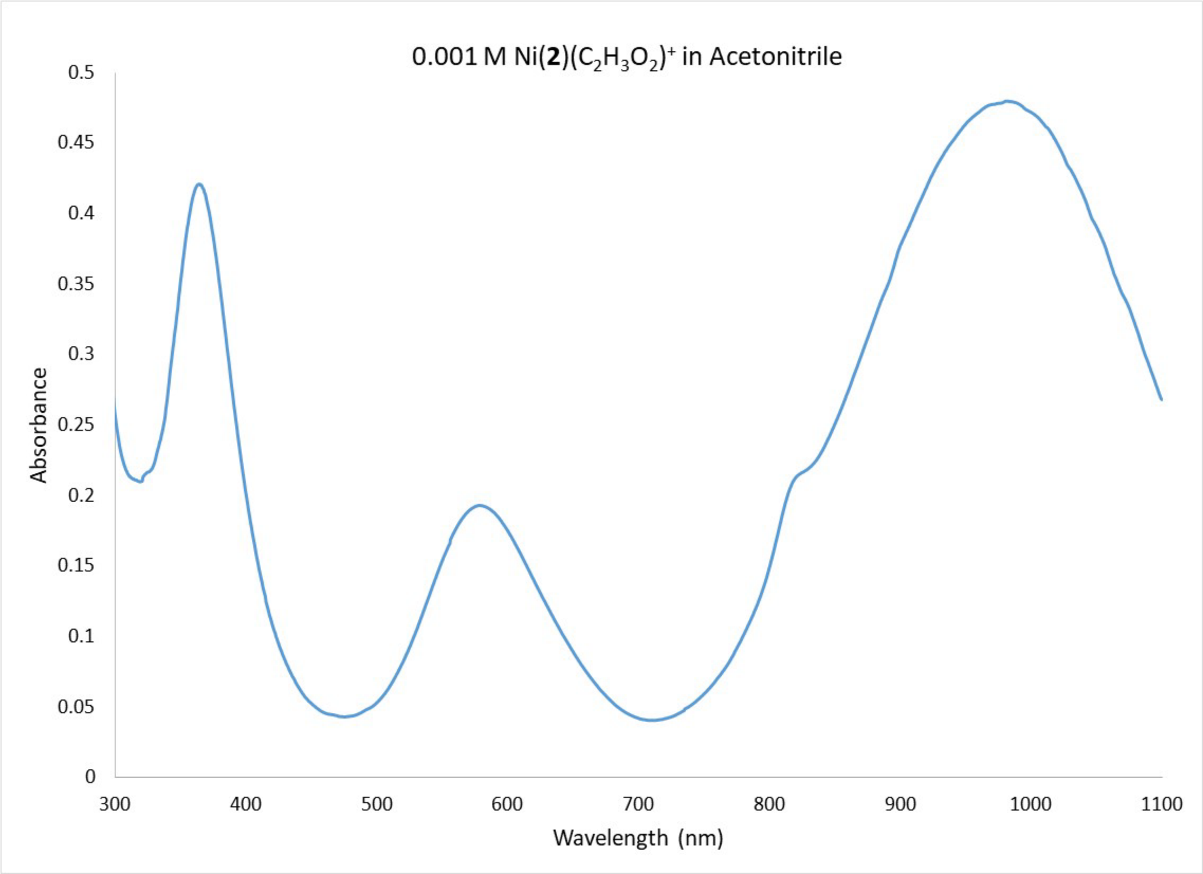

Figure S18. UV-Vis Spectrum of Ni(2)($C_2H_3O_2$)$^+$ in Acetonitrile.

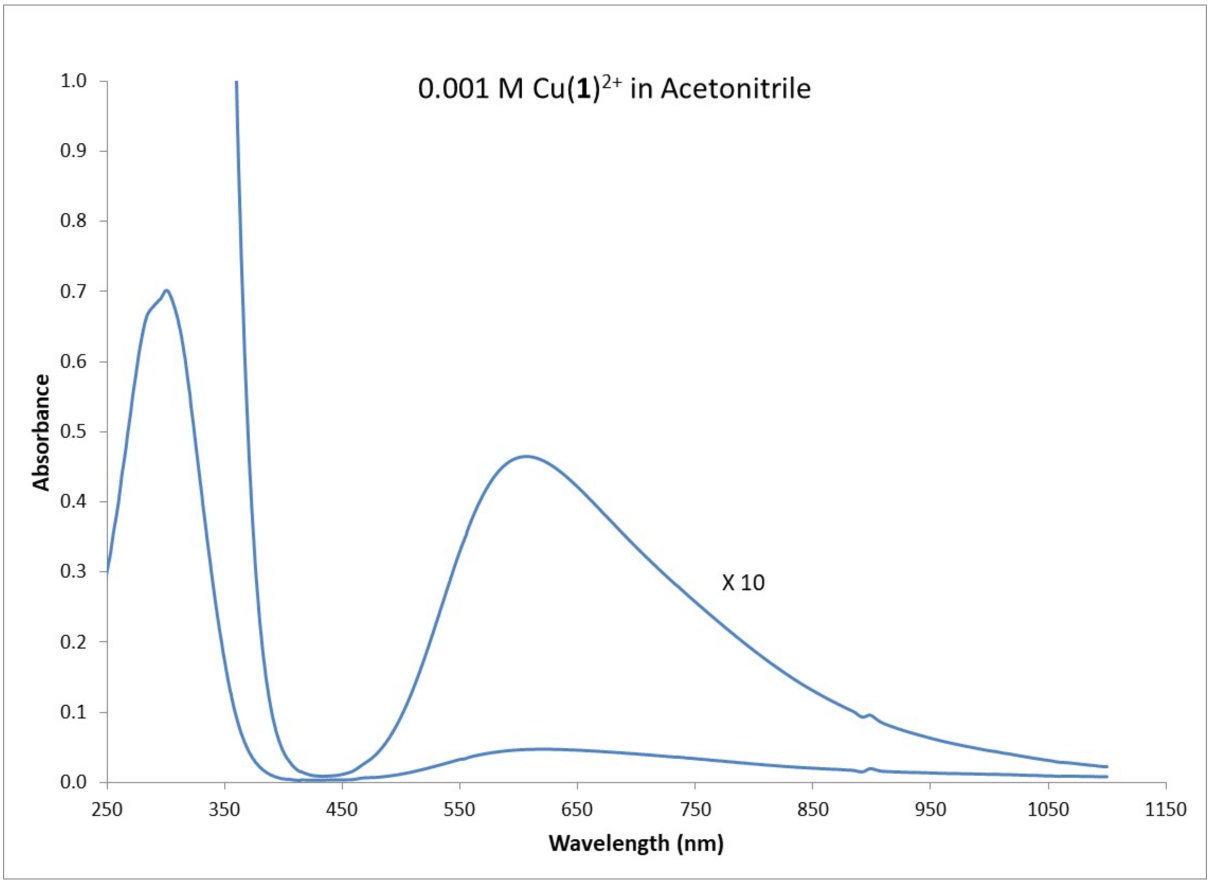

Figure S19. UV-Vis Spectrum of Cu(1)$^{2+}$ in Acetonitrile.

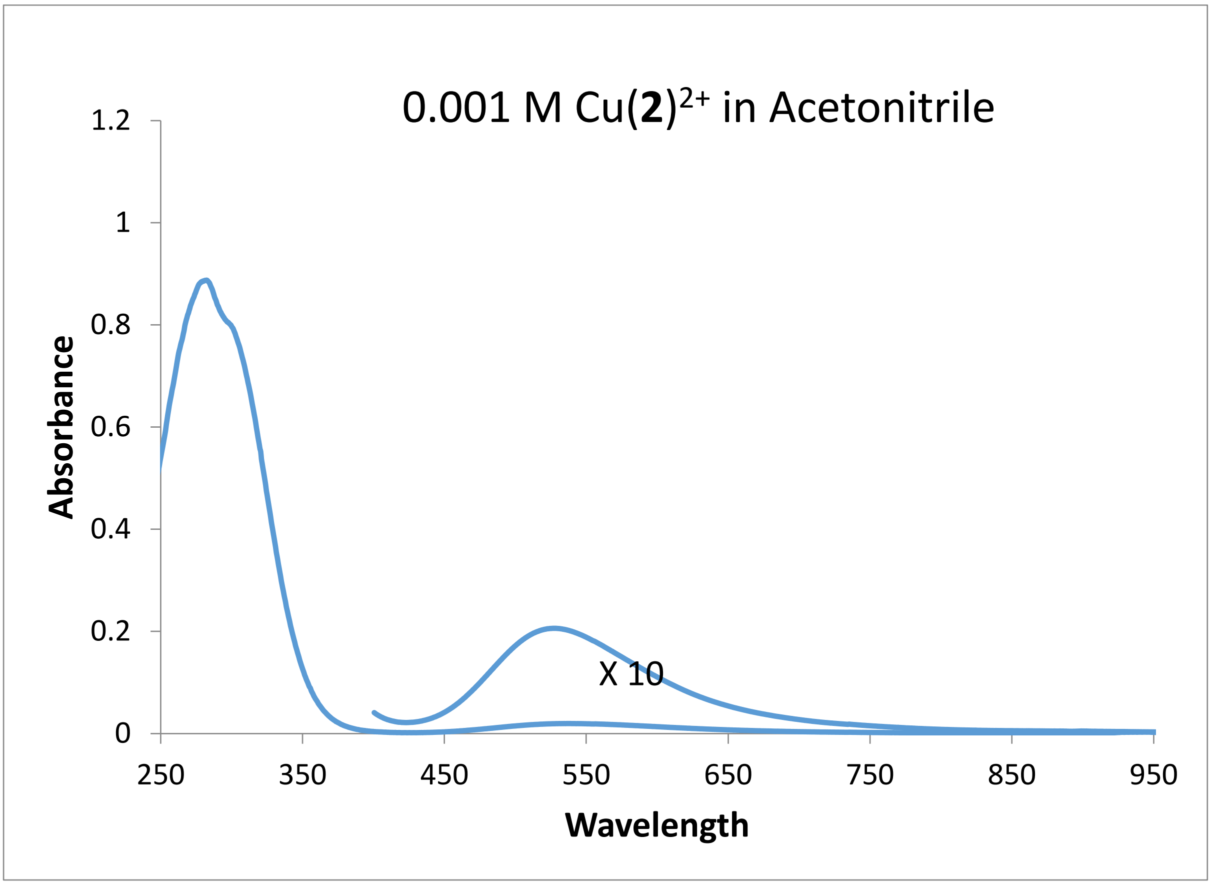

Figure S20. UV-Vis Spectrum of Cu(2)$^{2+}$ in Acetonitrile.

Example Kinetic Study for Dissociation of [Cu(1)]PF_6 in H_20 5M HCl 30°C at 640nm

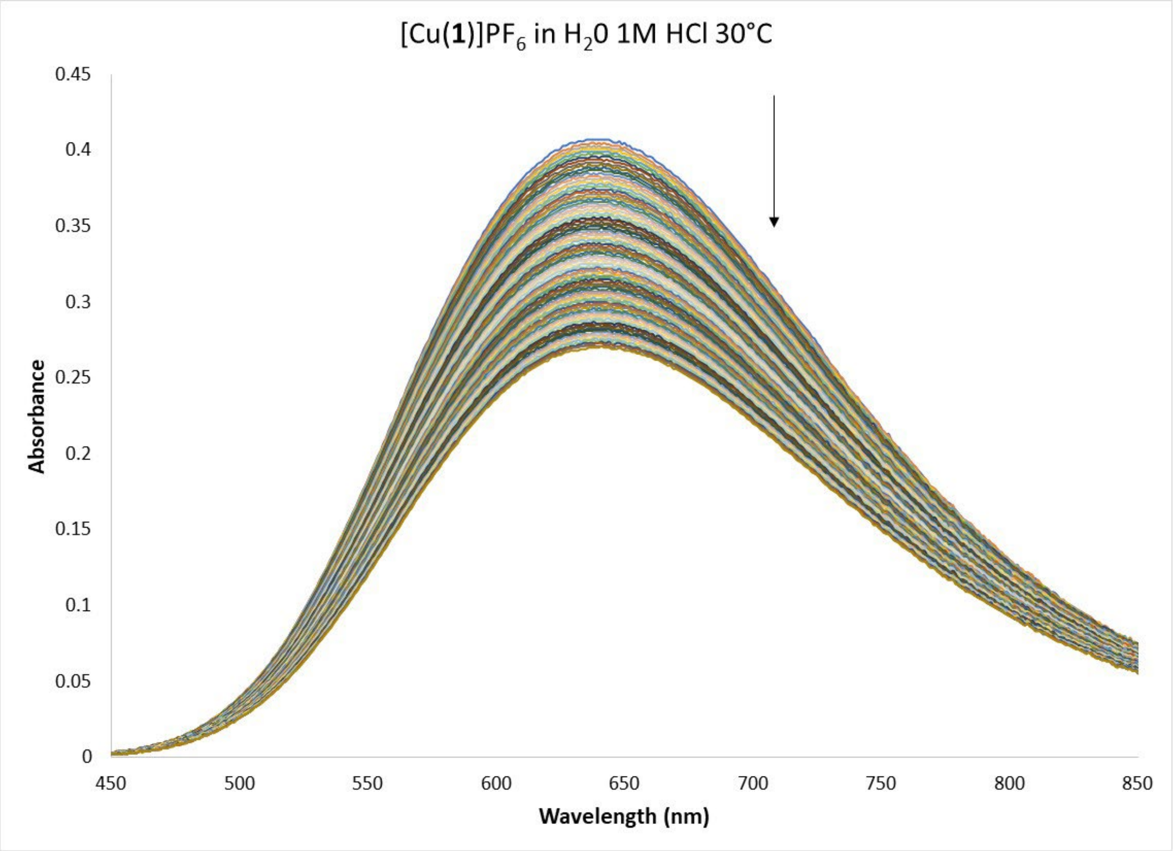

Figure S21. UV-Vis Spectrum of [Cu(1)]PF_6 in H_20 5M HCl 30°C at 640nm over time.

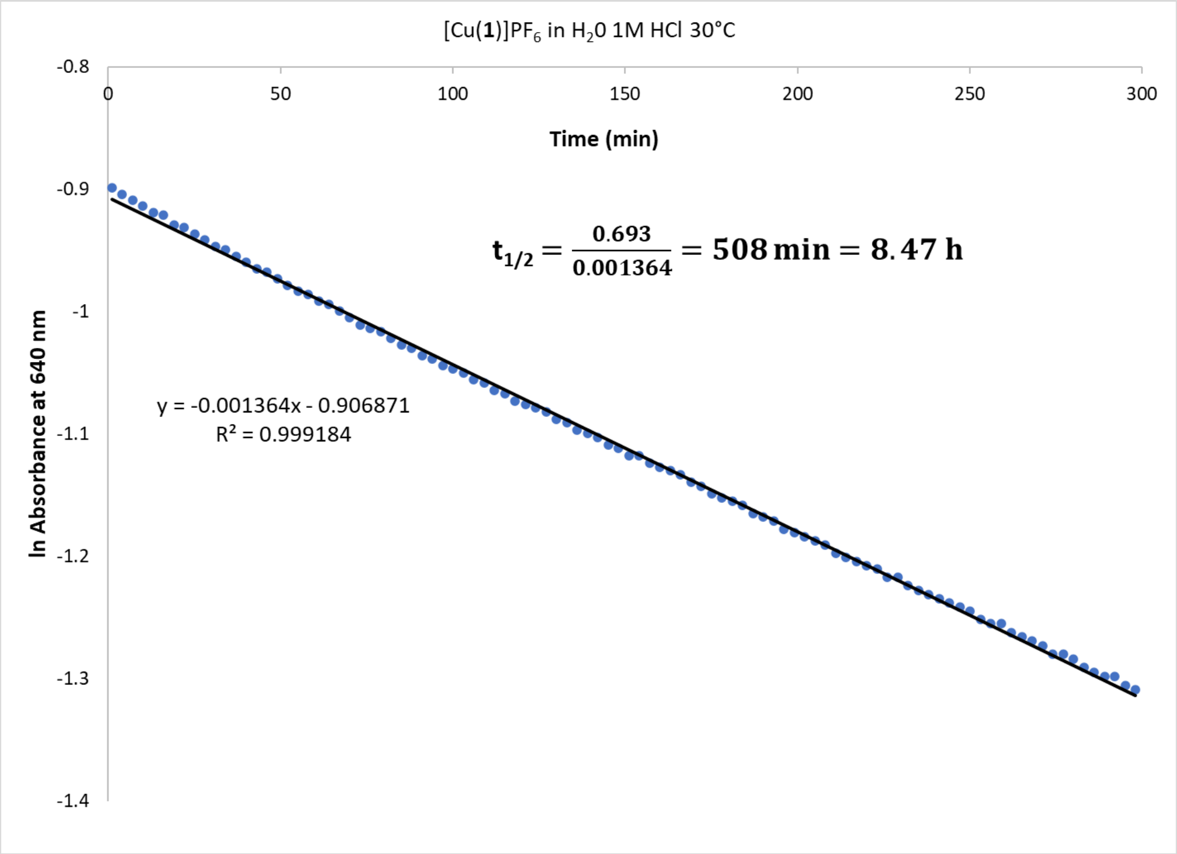

Figure S22. Pseudo-first order kinetic plot of decomposition data to determine the half-life.
